# PfMSA180 is a novel *Plasmodium falciparum* vaccine antigen that interacts with human erythrocyte integrin associated protein (CD47)

**DOI:** 10.1038/s41598-019-42366-9

**Published:** 2019-04-11

**Authors:** Hikaru Nagaoka, Chisa Sasaoka, Takaaki Yuguchi, Bernard N. Kanoi, Daisuke Ito, Masayuki Morita, Rachanee Udomsangpetch, Jetsumon Sattabongkot, Tomoko Ishino, Takafumi Tsuboi, Eizo Takashima

**Affiliations:** 1 Division of Malaria Research, Proteo-Science Center, Ehime University, 3 Bunkyo-cho, Matsuyama, Ehime 790-8577 Japan; 2 Division of Medical Zoology, Department of Microbiology and Immunology, Faculty of Medicine, Tottori University, 86 Nishi-cho, Yonago, Tottori 683-8503 Japan; 3 Center for Research and Innovation, Faculty of Medical Technology, Mahidol University, Salaya, Nakhosn Pathom 73170 Thailand; 4 Mahidol Vivax Research Unit, Faculty of Tropical Medicine, Mahidol University, Bangkok, 10400 Thailand; 5 Division of Molecular Parasitology, Proteo-Science Center, Ehime University, Toon, Ehime, 791-0295 Japan

## Abstract

Malaria symptoms and pathology are initiated by invasion of host erythrocytes by *Plasmodium* merozoites in a complex process that involves interactions between parasite and host erythrocyte proteins. Erythrocyte invasion presents attractive targets for malaria vaccine and drug development. Recently it was observed that antibodies against PfMSA180 (PF3D7_1014100) are associated with protection from symptomatic malaria, suggesting that this protein is a target of naturally acquired protective antibodies. Here we characterize PfMSA180, a ~170 kDa merozoite surface antigen that is potentially involved in erythrocyte invasion. PfMSA180 synthesized by the wheat germ cell-free system was used to raise antibodies in rabbits. Growth inhibition assays revealed that parasite invasion is inhibited by antibodies to the PfMSA180 C-terminal region, which contains an erythrocyte-binding domain. Surface plasmon resonance analysis showed that PfMSA180 specifically interacts with human erythrocyte integrin associated protein (CD47), suggesting that PfMSA180 plays a role during merozoite invasion of erythrocytes. Polymorphism analysis revealed that *pfmsa180* is highly conserved among field isolates. We show that naturally acquired PfMSA180-specific antibodies responses are associated with protective immunity in a malaria-exposed Thai population. In sum, the data presented here supports further evaluation of the conserved erythrocyte-binding C-terminal region of PfMSA180 as an asexual blood-stage malaria vaccine candidate.

## Introduction

*Plasmodium falciparum* malaria is a major cause of death in young children and pregnant women in developing and underdeveloped countries^1^. Despite numerous intervention strategies that have substantially reduced the burden of malaria, the fight against the disease has been complicated by the emergence of drug resistant parasites and insecticide-resistant mosquitoes^2,3^. Development of an effective vaccine is therefore considered a critical global agenda towards significant malaria control and eventual elimination^4^.

Malaria symptoms and pathology are the result of invasion of host erythrocytes by *Plasmodium* merozoites in a complex series of well-orchestrated molecular events involving interactions of parasite and host erythrocyte proteins^5,6^. This key stage presents an attractive target for vaccine and drug development^7,8^. Unfortunately, few identified antigens have progressed to clinical development as vaccine components or have then demonstrated robust efficacy in African fields trials^9–11^. Major hurdles in vaccine development include the induction of strain-specific immune responses, antigenic variation and allelic diversity of candidate antigens, and a lack of a clear understanding of the mode of action of candidate vaccines^11–14^.

Application of the annotation information of the *P. falciparum* genome is expediting the discovery of new vaccine candidate antigens^15,16^, such as the recent discovery of the vaccine candidate antigens *P. falciparum* Cysteine-Rich Protective Antigen (PfCyRPA), PfRH5, PfRIPR, CelTOS, and P27^17–22^. Continued efforts will help to describe the biology of invasion, as well as contribute to the repertoire of antigens that are key targets of potent invasion-inhibitory antibodies.

Recently a screen for potential targets of naturally acquired protective immunity in malaria indicated that antibodies against *P. falciparum* PF3D7_1014100 are associated with protection from symptomatic malaria, suggesting that this protein is a target of protective antibodies^23^. In this study we describe the functional characterization of PF3D7_1014100 (named merozoite surface antigen 180, PfMSA180) as a *P. falciparum* merozoite surface protein that is likely involved in erythrocyte invasion. We used the wheat germ cell-free system (WGCFS) to express and evaluate recombinant PfMSA180 protein. Like other invasion related proteins^7,8^, PfMSA180 is highly expressed in schizonts; as a ~170 kDa protein that localizes to the surface of mature merozoites. We demonstrate that PfMSA180 directly interacts with the human erythrocyte protein CD47 (integrin associated protein - IAP). Furthermore, antibodies against PfMSA180 inhibit merozoite invasion *in vitro*. Taken together, the data presented here shows that PfMSA180 warrants evaluation as a candidate malaria vaccine.

## Results

### PfMSA180 exists as a 170-kDa molecule in parasites

**Figure 1:**
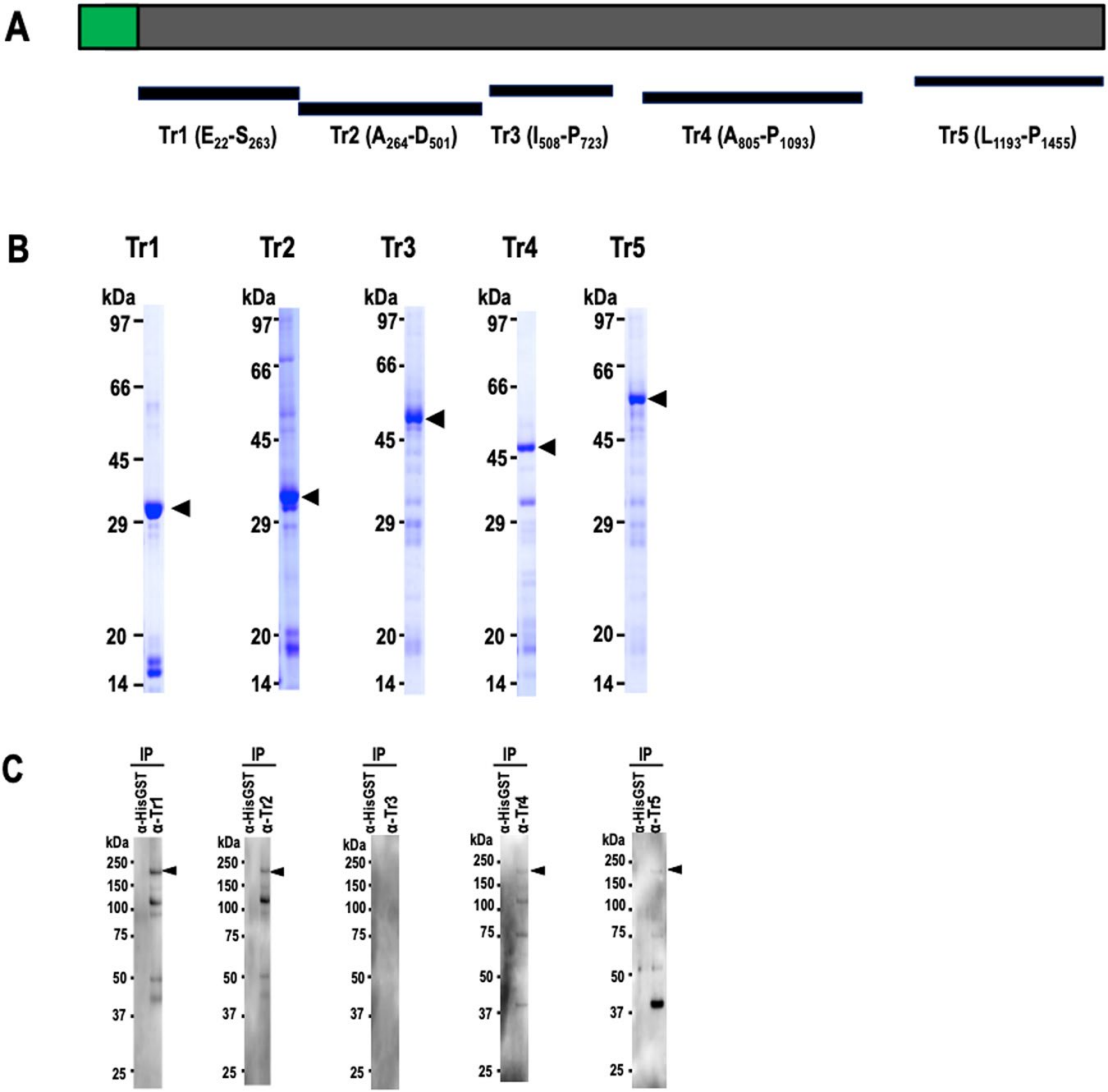
PfMSA180 (PF3D7_1014100) exists as a 170 kDa protein in parasites. (**A**) Schematic representation of full-length PfMSA180 and recombinant protein fragments (Tr 1–5) PfMSA180 consists of 1455 aa with a calculated molecular mass (MW) of 173.3 kDa. The protein has a predicted signal peptide (SP; 1 to 22 aa; shown in green). Recombinant PfMSA180 truncates were expressed as N-terminal GST-tagged proteins by the wheat germ cell-free system (WGCFS). Tr1, residues E_22_-S_263_ (expected MW 29 kDa); Tr2, A_264_-D_501_ (expected MW 27.9 kDa); Tr3, I_508_-P_723_ (expected MW 52.8 kDa, including the GST tag); Tr4, A_805_-P_1093_ (expected MW 34 0.4 kDa); and Tr5, L_1193_-P_1455_ (expected MW 58.5 kDa including the GST tag). (**B**) Recombinant PfMSA180 truncates are shown stained with Coomassie brilliant blue (CBB) following purification on a glutathione-Sepharose 4B column and resolution by 12.5% SDS-PAGE under reducing conditions. Arrowheads indicate molecular masses predicted from amino acid sequences of the corresponding recombinant PfMSA180 truncates (original data is included in the Supplementary Data File 1). (**C**) Reactivity of rabbit anti-PfMSA180 antibodies to native PfMSA180. Schizont-rich parasite pellets were solubilized with NP40 and PfMSA180 immunoprecipitated with the indicated mouse antibodies. Mouse anti-HisGST antibody was used as a negative control. Immunoprecipitated full length PfMSA180 was detected using rabbit antibody to each of the truncates; indicated using an arrowhead. The additional bands observed in the membrane fraction at 120, 80, and 45 kDa, were likely products of protein SUB1-mediated proteolysis of full-length protein. Antibodies to truncate 3 did not immunoprecipitate protein (original data is included in the Supplementary Data File 1).

To characterize the expression and function of PfMSA180, firstly, N-terminal glutathione S-transferase (GST) tagged truncates of recombinant PfMSA180 (Fig. 1A) were synthesized using the wheat germ cell-free system (WGCFS) (CellFree Sciences, Matsuyama, Japan**)**^24^. Repeated attempts to express full-length PfMSA180 were unsuccessful and thus all studies were performed with truncates synthesized as soluble proteins. The truncates (Tr) Tr1, residues E_22_-S_263_; Tr2, A_264_-D_501_; Tr3, I_508_-P_723_; Tr4, A_805_-P_1093_; and Tr5, L_1193_-P_1455_ were affinity purified using a glutathione-Sepharose 4B column (GE Healthcare, Camarillo, CA). The captured proteins were eluted by either on-column cleavage with AcTEV protease (Thermo Fisher Scientific, Waltham, MA) (Tr1, 2 and 4), targeting a tobacco etch virus (TEV) protease recognition site located between the GST tag and the recombinant protein, or with 20 mM glutathione (Tr3 and 5). The purified proteins were resolved on a 12.5% SDS-polyacrylamide gel and stained with Coomassie brilliant blue R-250 (Nacalai Tesque Inc, Kyoto, Japan) as shown in Fig. 1B. All truncates were used to immunize and raise antibodies in mice and rabbits.

We evaluated whether rabbit anti-PfMSA180 antibodies could detect *P. falciparum* 3D7 parasite native PfMSA180. Schizont-rich parasites were solubilized by NP40 (Nacalai Tesque Inc, Kyoto, Japan) followed by protein immunoprecipitation using mouse anti-PfMSA180 antibodies. In Western blot analysis PfMSA180 was detected as a ~170 kDa band (173 kDa predicted from the amino acid sequence) by rabbit antisera to all truncates (Tr1, 2, 4, 5) except Tr 3 fragment (Fig. 1C). Additional 120, 80, and 45 kDa bands observed in the membrane fraction were likely products of PfSUB1-mediated proteolysis of full-length protein^25^ (Fig. S1). These findings validate the reactivity of the raised antibodies as well as confirm native expression of PfMSA180 by parasites.

### PfMSA180 is expressed in the schizont and localizes on the merozoite surface

**Figure 2:**
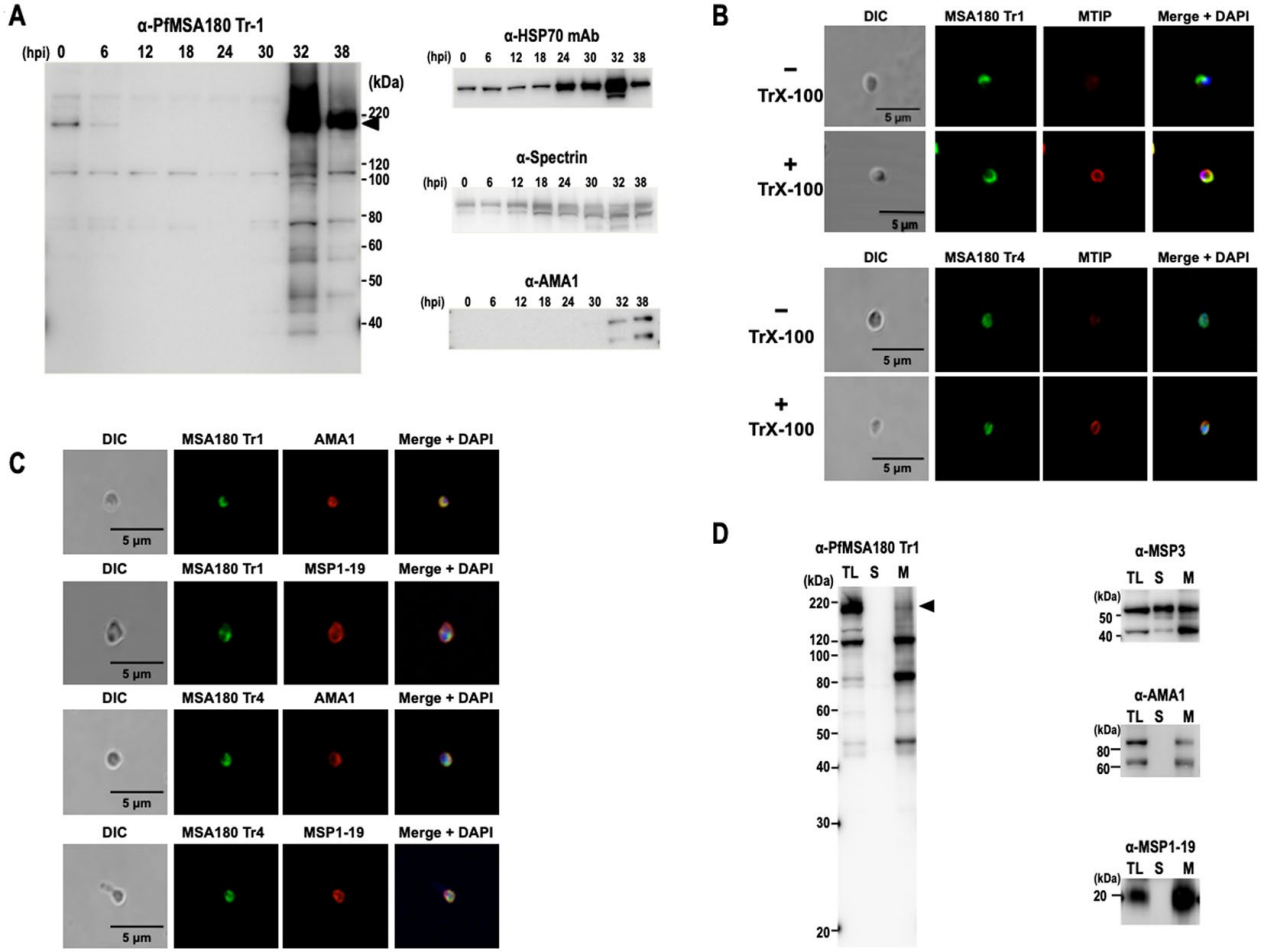
PfMSA180 is expressed in schizonts and localizes on the merozoite surface. (**A**) A time course immunoblot analysis of 3D7 parasite extracts shows that PfMSA180 is synthesized late in the intraerythrocytic cycle. The protein extracts prepared from Percoll-sorbitol-synchronized parasites were electrophoresed on SDS-12.5% PAGE and probed with rabbit polyclonal anti-PfMSA180 Tr1 antibodies. Hours post-invasion (hpi) are indicated for each lane. Anti-PfHSP70 monoclonal antibody was used as a quantitative parasite protein marker, anti-human spectrin α I rabbit antibody (Santa Cruz Biotechnology, Dallas, TX) indicating the number of loaded erythrocytes^49^, and anti-AMA1 antibody was used as a mature schizont stage marker (original data is included in the Supplementary Data File 1). (**B**) IFA analysis of PfMSA180 with MTIP (myosin A tail domain interacting protein). Free merozoites were processed with (+) or without (−) permeabilization using 0.1% Triton X-100 (TrX-100). The merozoites were stained with anti-PfMSA180-Tr1 antibody (upper panels, green color) or anti-PfMSA180-Tr4 (lower panels, green color) and co-stained with anti-MTIP antibody (red color). The leftmost panels show pictures of differential interference contrast (DIC). The rightmost panels show merged pictures with DAPI, showing localization of parasite’s nucleus. (**C**) IFA analysis of PfMSA180 with AMA1 and MSP1-19. Free merozoites were permeabilized with 0.1% Triton X-100. The merozoites were stained with anti-PfMSA180-Tr1 antibody (upper panels, green color) or anti-PfMSA180-Tr4 (lower panels, green color) and co-stained with anti-AMA1 antibody or anti-MSP1-19 antibody (red color). The leftmost panels show pictures of differential interference contrast (DIC). The rightmost panels show merged pictures with DAPI, showing localization of parasite’s nucleus. (**D**) Fractionation of PfMSA180 due to solubility. Schizont-rich 3D7 parasites were disrupted by sonication and fractionated with ultracentrifugation. The resulting soluble and membrane fractions were applied to SDS-PAGE. Immunoblotting analysis with anti-Tr1 antibody detected PfMSA180 in the membrane fraction. MSP3, a secreted protein was detected in both membrane and soluble fractions. AMA1, an integral membrane protein, and MSP1-19, a peripheral GPI-anchored membrane protein, were detected in the membrane fraction. TL: total schizont-rich parasite lysate; S: soluble fractions; M: membrane fraction. Original data is included in the Supplementary Data File 1.

To assess the stage-specific protein expression of PfMSA180 during the *P. falciparum* intraerythrocytic developmental cycle, we conducted a 6-hr interval time-course immunoblot analysis of synchronized 3D7 parasites using PfMSA180-Tr1-specific antibodies. A discrete band at ~170 kDa was detected in immature and mature schizont stages (32 and 38 hours post invasion; hpi) as well as in early ring stages (0 to 6 hpi), but not in trophozoite stages (12 to 30 hpi) (Fig. 2A), consistent with transcriptome analysis^26^. As a control, monoclonal antibodies to the house-keeping protein HSP70 showed constitutive expression with increase in late schizonts. Human spectrin was detected in all stages while parasite AMA1 was detected only in the immature and mature schizont stages (Fig. 2A). These control experiments indicated comparable quantities of loaded infected erythrocytes and normal parasite growth.

We subsequently sought to determine the sub-cellular localization of native PfMSA180 in free merozoites using immunofluorescence assays (IFA) (Fig. 2B). To confirm surface localization of PfMSA180, we conducted IFA analysis with or without Triton X-100 permeabilization. Myosin A tail domain interacting protein (MTIP), a protein that localizes to the inner membrane complex of *Plasmodium* merozoites, was co-stained as a marker. While permeabilized merozoites showed strong uniform peripheral distribution of MTIP, unpermeabilized merozoites showed weak background peripheral distribution (Fig. 2B). In contrast, PfMSA180 was unevenly localized around both permeabilized and unpermeabilized merozoites (Fig. 2B), suggesting that PfMSA180 localizes to the merozoite surface, consistent with our previous report of the *P. vivax* ortholog, PvMSA180^27^. Co-staining with markers directed against the merozoite apical region (AMA1) and surface (MSP1-19) revealed a significant overlap with both proteins, with AMA1 having the greatest overlap (Fig. 2C). Thus, the data suggests that PfMSA180 localizes to the apical region surface of free merozoites.

PfMSA180 has an N-terminal signal peptide but lacks a predicted transmembrane domain. Since PfMSA180 is translocated to the apical surface of invasive merozoites, we sought to assess if PfMSA180 is a membrane-associated protein. To this end, we performed fractionation experiments with lysates of synchronized schizont-rich parasites. Consistent with the IFA data, PfMSA180 was detected in the membrane fraction indicating membrane localization (Fig. 2D). The PfMSA180 band (170 kDa) diminished after fractionation. Secreted protein MSP3 was detected in both the membrane and soluble fractions, as described^28^. AMA1, an integral membrane protein^29^, and MSP1-19, a peripheral GPI-anchored membrane protein^30^, were both detected in the membrane fractions (Fig. 2D).

### Antibodies to PfMSA180 inhibit merozoite invasion *in vitro* and PfMSA180 is naturally immunogenic in a malaria exposed population

**Figure 3:**
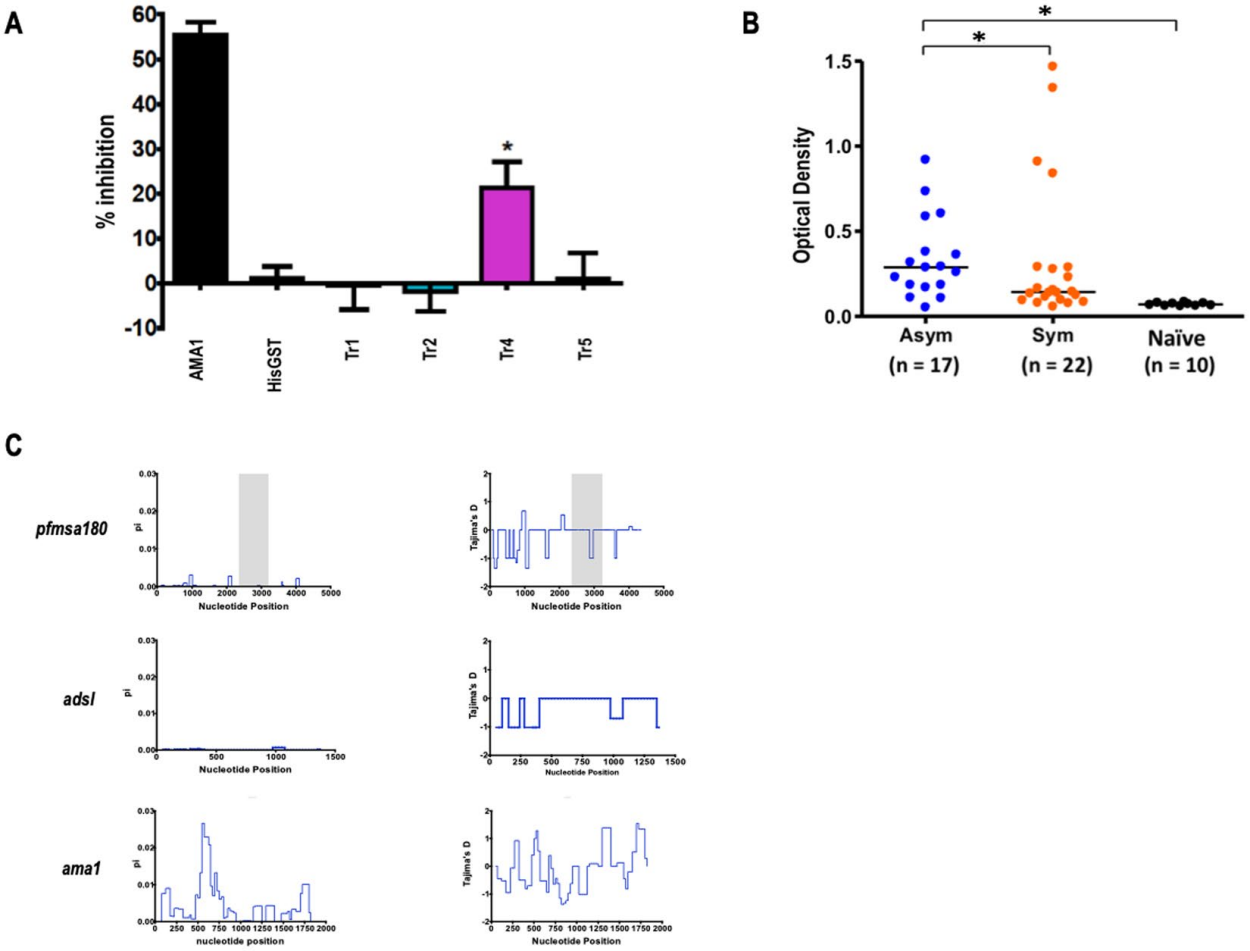
PfMSA180 is immunogenic in malaria exposed population and antibodies to PfMSA180-Tr4 inhibit merozoite invasion. (**A**) Growth inhibition assay (GIA) activity was determined for antibodies against PfMSA180 on *in vitro* cultured *P. falciparum* 3D7 isolate parasites. Rabbit antibody to AMA1 was used as a positive control with anti-HisGST antibodies as a negative control. Error bars represent standard error of the mean. An asterisk indicates statistical significance (Kruskal-Wallis test; P < 0.05) when compared with antibodies to HisGST. (**B**) Antibodies to PfMSA180-Tr4 in individuals exposed to malaria were determined by ELISA. The individuals were categorized as symptomatic (Sym), asymptomatic (Asy), or non-exposed malaria naïve (Naïve). The number (n) of samples analyzed is shown in parentheses. Average optical density (OD) values of two independent measurements are shown as dot plots. Horizontal bar represents the median OD values in each group. *P* values were calculated by Kruskal-Wallis test followed by Dunn’s multiple-comparison test. *Represent statistical significance at *P* < 0.01. (**C**) Sliding window analysis showing nucleotide diversity (π) values and Tajima’s D statistic in *pfmsa180*, suggesting no balancing selection. The gray box represents Tr4, PfMSA180 amino acid residues 805 to 1093. The highly conserved housekeeping gene, *adsl*, and highly polymorphic protein, *ama1*, were analyzed as controls.

Having observed that PfMSA180 is a merozoite apical surface protein, we sought to determine if the protein has a role in erythrocyte invasion. Specifically, we tested the potency of rabbit polyclonal antibodies recognizing PfMSA180 truncates to block parasite invasion *in vitro* over one cycle of parasite replication. At a final total IgG concentration of 20 mg/ml, PfMSA180-Tr4 showed significant inhibition of invasion of 21.3% ± 5.0% (mean ± SEM), compared to the negative control, antibodies to HisGST (P < 0.05; Fig. 3A). As a positive control, antibodies to AMA1 showed GIA activity of 55.4% ± 2.4% (mean ± SEM). Antibodies recognizing the other truncates showed no GIA activity. These results suggest that Tr4 is a critical PfMSA180 region regarding erythrocyte invasion.

Since antibodies to PfMSA180-Tr4 inhibited merozoite invasion *in vitro* (Fig. 3A), we investigated whether humans naturally exposed to *P. falciparum* infection acquire protective anti-PfMSA180-Tr4 antibodies. We assessed antibodies in human sera obtained from *P. falciparum*-infected symptomatic and asymptomatic adults in Thailand. Significantly higher antibodies levels against PfMSA180-Tr4 were observed in individuals who had asymptomatic malaria compared to those who had symptomatic malaria (*P* < 0.01, Kruskal-Wallis test; Fig. 3B). Sera from healthy Thai adults with no history of malaria exposure served as malaria naïve controls. These results suggest that PfMSA180 is naturally immunogenic during *P. falciparum* infection and that the antigen is a potential target of protective antibodies in falciparum malaria.

We further calculated nucleotide diversity (π) and Tajima’s D, indicative of diversifying selection, within the *pfmsa180* gene using data of approximately 200 field isolates from different sites in west and east Africa as deposited in PlasmoDB (http://plasmodb.org/). Both metrics did not show any high peaks suggesting the absence of balancing selection within the Tr4 region (Fig. 3C, gray area). The lack of balancing selection could indicate high conservation of the gene across isolates. A highly conserved housekeeping gene, *adsl*, and the polymorphic protein, AMA1, were analyzed as controls. There are 67 non-synonymous alleles in *pfmsa180*, with 26 localized within the Tr4 region (289 aa). Multiple sequence analyses with the 3D7 reference sequence, African isolates and available Asian sequences showed high degrees of gene conservation (Supplemental Data File 2). In summary, PfMSA180 presents a potentially conserved target of protective antibodies in malaria.

### PfMSA180 interacts with CD47, a host erythrocyte surface protein

**Figure 4:**
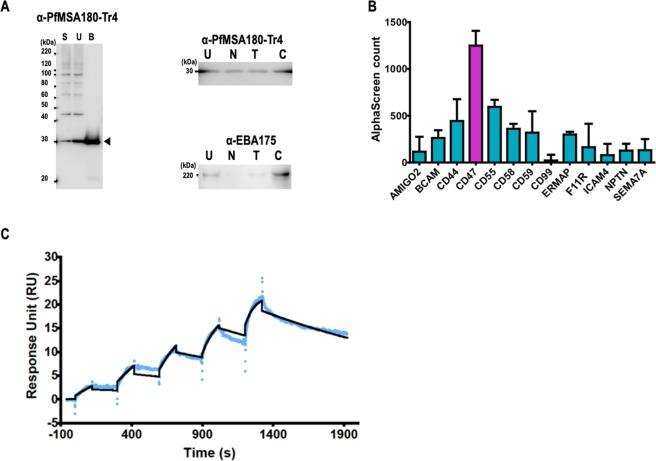
PfMSA180 interacts with CD47, a human erythrocyte surface protein. (**A**) Erythrocyte binding activity of native PfMSA180 protein compared to EBA175. Left panel: S, parasite culture supernatant; U, erythrocyte unbound fraction; B, untreated erythrocyte bound fraction. Arrowhead indicates the bound fragment of PfMSA180. Right panels: U, unbound fraction to the untreated erythrocytes; N, neuraminidase-treated erythrocytes; T, trypsin-treated erythrocytes; C, chymotrypsin-treated erythrocytes. Original data is included in the Supplementary Data File 1. (**B**) AlphaScreen reactivity profile of recombinant PfMSA180-Tr4 to 13 GST-fused recombinant proteins. Biotinylated recombinant PfMSA180-Tr4 was mixed with each of 13 GST-fused erythrocyte surface proteins and incubated for 1 h at 26 °C to form a protein-protein complex. Subsequently, a suspension of streptavidin-coated donor-beads and anti-GST acceptor-beads mixture in reaction buffer was added. The mixture was incubated for 12 h at 26 °C in dark. This allowed the donor- and acceptor-beads to optimally bind to biotin and GST, respectively. Upon illumination of this complex, a luminescence signal at 620 nm was detected by the EnVision plate reader (PerkinElmer) and the result was expressed as AlphaScreen counts. HisGST was used to set the background and was subtracted from each sample signal. Each bar represents the average AlphaScreen counts in quintuplicate with error bars representing SE of the mean. CD47 had the highest mean signal. (**C**) Sensorgram of SPR single-cycle kinetic analysis. Recombinant CD47 was immobilized on a CM5 chip and used as the ligand while recombinant PfMSA180-Tr4 was used as analyte. The blue curve represents the data-generated sensorgram while the black curve indicates line of fit used to calculate kinetics parameters. All assays were performed at an increasing protein concentration of 0.125, 0.25, 0.5, 1, and 2 µM at 120 s contact time and 180 s dissociation time. The dissociation time in the last cycle was extended to 580 s.

Having observed that PfMSA180 is a conserved apical surface protein that induces antibodies with GIA activity, we sought to understand if the protein interacts with erythrocyte surface proteins. Merozoite proteins involved in erythrocyte invasion are known to be shed into the culture medium (culture supernatant) during the invasion process^31,32^, and thus cell culture supernatant can be used as a source of soluble *P. falciparum* parasite proteins. To determine the erythrocyte binding activity of native PfMSA180, erythrocyte binding assays (EBAs) were performed by incubating untreated and enzyme (neuraminidase, trypsin, and chymotrypsin) treated human erythrocytes with parasite culture supernatants as reported^21,33^. In immunoblots of parasite culture supernatant probed with anti-PfMSA180-Tr4 antibody, we observed a 30 kDa band of native processed PfMSA180 that bound untreated as well as neuraminidase, trypsin, and chymotrypsin treated erythrocytes (Fig. 4A). This suggests that the erythrocyte binding phenotype of PfMSA180 is sialic acid independent (neuraminidase resistant) as well as trypsin and chymotrypsin resistant. The erythrocyte binding activity of native EBA175 was also analyzed from the same culture supernatant as a control for the enzymatic treatments of the human erythrocytes. We observed that the binding pattern of EBA175 was in agreement with reported data that EBA175 binds sialic acid residues of erythrocyte glycophorin A in a neuraminidase and trypsin sensitive manner^33^ (Fig. 4A).

To identify a potential receptor for PfMSA180-Tr4 on the surface of host erythrocytes, we used a systematic screening approach by first compiling a library of 13 proteins that are abundantly expressed on the erythrocyte surface^34^ (Table S1) and synthesizing them as N-terminal GST and C-terminal His-tagged recombinant proteins using WGCFS. We applied the AlphaScreen protein-protein interaction assay^35–37^ to screen for potential binding receptors against PfMSA180-Tr4 (Fig. S2A). Integrin associated protein (IAP or CD47) had the highest and distinct signal among the 13 erythrocyte proteins, suggesting its interaction with PfMSA180 (Fig. 4B). Compared to PfMSA180-Tr1, Tr4 showed stronger reactivity with CD47 (Fig. S2B).

To validate the interaction between PfMSA180-Tr4 and CD47, we analyzed the strength of interaction between the two recombinant proteins using surface plasmon resonance (SPR). We established that recombinant PfMSA180-Tr4 at increasing concentration of 0.125, 0.25, 0.5, 1, and 2 µM, directly interacts 1:1 with recombinant CD47 with an equilibrium binding constant (*K*_D_) value of 2.06 × 10^−9^ M (Fig. 4C). HisGST, CD55 -which ranked second in the AlphaScreen experiment (Fig. 4B), and PfMSA180-Tr1 served as negative controls and showed no interaction with CD47 (Fig. S3). Put together, the data here suggest that CD47 is a putative erythrocyte receptor for the C-terminal region of native PfMSA180.

## Discussion

Invasion of erythrocytes by *Plasmodium* parasites is a complex process involving multiple parasite and host proteins^6,38^. Several molecular interactions during invasion have been evaluated, although complete understanding of this complex biological process remains unknown. Identification and characterization of erythrocyte invasion proteins will not only further clarify the molecular basis of erythrocyte invasion but will also increase our chances of developing effective interventions against malaria.

Recently, while screening for targets of protective immunity in malaria, we observed lower antibody responses against PfMSA180 (PF3D7_1014100) in individuals with symptomatic malaria than those with asymptomatic infection, suggesting that the protein is a target of naturally acquired protective immunity^23^. PfMSA180 possesses an N-terminal signal peptide and is predicted to be a secreted protein. In addition, PfMSA180 sequence is conserved across different *Plasmodium* species suggesting it has an important role in parasite development^27^. Indeed, a *P. vivax* orthologue of this protein was recently described as a vaccine candidate^27^. In our present study we sought to investigate the potential role of PfMSA180 in erythrocyte invasion. Our data clearly revealed that PfMSA180 possesses propitious features that make it a target for vaccine development. Firstly, genetic analysis of African isolates showed that *pfmsa180* is relatively conserved among parasite populations in malaria endemic regions in Africa compared to leading blood-stage vaccine antigens like AMA1. This observation consolidated the suggestion that PfMSA180 has a critical and conserved functional role in parasite development. Secondly, antibodies against PfMSA180-Tr4 showed growth inhibition activity *in vitro*, and this may be because PfMSA180 plays a role in anchoring merozoites to the host erythrocyte surface. This is consistent with recent piggyBac transposon mutagenesis study showing that *pfmsa180* is an essential gene^39^.

In the present study, we have demonstrated that PfMSA180 is localized on the surface of the invasive merozoite, despite lacking a membrane anchoring domain. It is plausible that PfMSA180 might be interacting with other parasite proteins that escort and anchor it on the merozoite surface. Elucidation of the mechanism of its association to the membrane remains a question for further investigation.

**Figure 5:**
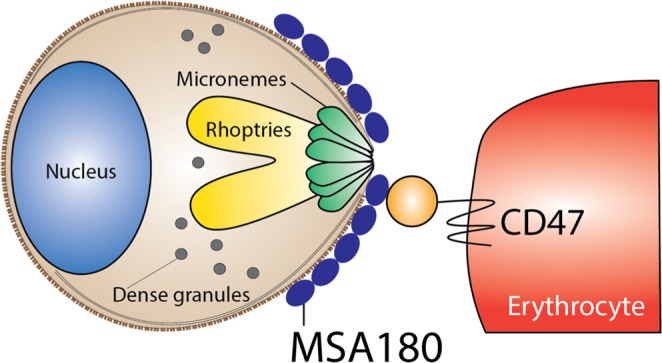
Schematic representation showing the interaction of PfMSA180 and CD47 during erythrocyte invasion. PfMSA180 localizes at the merozoite apical end and binds to CD47 on the surface of erythrocytes. This interaction is important for merozoite invasion and could be the target of antibodies against PfMSA180-Tr 4.

Like the merozoite surface invasion ligands MSP1, MSP3, and MSP7, PfMSA180 may be sequentially cleaved to produce mature functional fragments^25,40^. A 30 kDa fragment of native PfMSA180 released into culture medium binds untreated as well as neuraminidase, trypsin, and chymotrypsin treated erythrocytes (Fig. 4A); suggesting that the erythrocyte binding phenotype of PfMSA180 is sialic acid independent (neuraminidase resistant) as well as trypsin and chymotrypsin resistant. Processed PfMSA180 could therefore act as an adhesin molecule that mediates attachment of the merozoite to the erythrocyte surface via interaction with CD47 (Fig. 5).

CD47 is a protein “marker of self” that is expressed on cell membranes in humans and mice^41,42^. CD47 expression on erythrocytes is an inhibitory signal for phagocytosis mediated through its interaction with signal regulatory protein alpha (SIRPα) on the surface of splenic macrophages^42^. Nonlethal *P. yoelii* 17XNL parasites preferentially infect young RBCs expressing high levels of CD47 and consequently escape from splenic clearance^43^. Similarly, disruption of CD47 engagement to its native ligand SIRPα resulted in enhanced phagocytosis of *P. falciparum* infected erythrocytes^44^. *P. falciparum* merozoites may exploit the PfMSA180-CD47 interaction to predominantly invade erythrocytes expressing CD47 and thereby take advantage of negative signaling of CD47 to avoid early elimination by phagocytosis. Further investigation might test this hypothesis.

In conclusion, application of reverse vaccinology strategies approaches has resulted in the identification of blood-stage vaccine candidates such as PfRH5 and PfCyRPA that are currently under clinical evaluation^17–22^. In this study, our major objective was to identify and characterize novel merozoite antigens essential for erythrocyte invasion that induce protective antibodies. Consequently, the data presented here shows that the Tr4 region of PfMSA180 is highly conserved across isolates, directly interacts with human erythrocyte protein CD47, and induces antibodies that inhibit merozoite invasion *in vitro*. PfMSA180-Tr4 is therefore recommended for evaluation as an ideal candidate component of a multi-stage or multi-antigen malaria vaccine^45^.

## Materials and Methods

### Production of recombinant proteins and antisera

Antibodies to PfMSA180 were prepared as described^46^. Sequence encompassing amino acids in Tr1 to Tr5 were amplified from *P. falciparum* 3D7 genomic DNA by PCR by using sense primers with XhoI sites and antisense primers with NotI restriction sites (Table S1). The amplified fragments were then restricted and ligated into pEU-E0-GST-TEV, a wheat germ cell-free expression vector to fuse GST-tag and TEV recognition sites at the N-terminal of the recombinant proteins (CellFree Sciences**)**^46^. All antisera against the recombinant proteins were commercially purchased from Kitayama Labes Co., Ltd. (Ina, Japan). We used additional rabbit polyclonal antisera: anti-AMA1 (PF3D7_1133400), Q_25_-K_546_; anti-MSP3 (PF3D7_1035400), E_27_-H_354_; anti-MSP1_19 (PF3D7_0930300), N_1607_-S_1699_; anti-MTIP (PF3D7_1246400), M_1_-Q_204_; and anti-EBA175 (PF3D7_0731500), Q_761_-G_1298_, that were generated as described^46^.

Expression constructs of erythrocyte surface proteins were purchased from Mammalian Gene Collection (Funakoshi, Tokyo, Japan) and were expressed with WGCFS as N-terminal GST-fusion protein with a C-terminus His-tag (Table S1). Namely, genes encoding AMIGO2, M_1_-N_397_; BCAM, E_32-_A_555_; CD44, Q_21-_E_606_; CD47, K_24_-S_139_; CD55, D_35_-T_381_; CD58, S_30_-R_215_; CD59, L_26_-P_128_; CD99, D_27_-G_125_; ERMAP, H_30_-S_154_; F11 receptor, H_32_-V_238_; ICAM4, A_23_-G_272_; NPTN, Q_29_-L_221_; and SEMA7A, Q_45_-H_666_. The recombinant proteins were purified using a glutathione-Sepharose 4B column (GE Healthcare, Camarillo, CA) as described^46^.

### Parasite culture and Western blot analysis

The *P. falciparum* 3D7 strain was maintained in continuous culture of 2% hematocrit erythrocytes (blood group O+) from the Japanese Red Cross Society^47^. To obtain parasite pellets, mature schizonts were harvested by 70%/40% Percoll-sorbitol centrifugation. The pellets were treated with tetanolysin (Biological Laboratories, Inc., Campbell, CA) to remove hemoglobin without loss of parasite proteins, and washed with phosphate-buffered saline (PBS) containing Complete protease inhibitor (Roche, Mannheim, Germany). The pellets were stored at −80 °C until use.

For Western blot analysis, the purified, schizont-rich parasite pellets were lysed in an appropriate amount of reducing SDS-PAGE sample buffer and then incubated at 98 °C for 3 min. The lysate was centrifuged at 10,000 × g for 10 min at room temperature (RT), and supernatants were subjected to electrophoresis in a 12.5% polyacrylamide gel (ATTO, Tokyo, Japan). Proteins were blotted to a 0.2-μm polyvinylidene difluoride membrane (Hybond LFP; GE Healthcare) with a semidry blotting system (ATTO). The membranes were blocked with PBS-MT (PBS containing 5% [wt/vol] nonfat milk and 0.1% [vol/vol] Tween 20). Membrane strip were then immunostained with each antiserum at RT for 1 h, followed by horseradish peroxidase-conjugated secondary-antibody (GE Healthcare) probing, and visualized with Immobilon Western chemiluminescent horseradish peroxidase substrate (Millipore, Billerica, MA) on a LAS 4000 Mini luminescent-image analyzer (GE Healthcare). The relative molecular masses of the proteins were estimated with reference to Precision Plus protein standards (Bio-Rad, Hercules, CA). Immunoprecipitation assays were conducted as described^46^. All blots derived from the same experiment and were processed in parallel. Original data is included in the Supplementary Data File 1.

### Free-merozoite purification

Free merozoites were purified as reported^46^. Briefly, parasites were synchronized using sorbitol treatment and heparin (Mochida Pharmaceutical Co., Tokyo, Japan). Late-stage parasites (32 to 36 hpi) were purified (>95% purity) by magnetic separation with a MACS magnetic separator (Miltenyi Biotec, Cambridge, MA), incubated with 10 μM E64 (Sigma-Aldrich Corporation, St. Louis, MO) for 6 to 8 h, and pelleted. The schizonts were then resuspended in incomplete culture medium and filtered through a 1.2-μm Acrodisc 32-mm syringe filter (Pall Corporation, Port Washington, NY) to isolate free merozoites.

### Immunofluorescence assays (IFA)

Free merozoites isolated as described above were fixed at RT for 30 min with 4% paraformaldehyde - 0.0075% glutaraldehyde in PBS^46^. The merozoites were processed with or without permeabilization with 0.1% Triton X-100 in PBS at RT for 10 min, and blocked with 3% bovine serum albumin (BSA) in PBS at 37 °C for 30 min.

For IFA, the free merozoites were stained with primary antibodies diluted at the following concentrations in blocking solution at 37 °C for 1 h: rabbit anti-PfMSA180-Tr1 antibody, 1:500; rabbit anti- PfMSA180-Tr4, 1:500; mouse anti-MTIP antibody, 1:100; mouse anti-AMA1 antibody, 1:100; and mouse anti-MSP1-19 antibody, 1:100. Secondary antibodies, Alexa Fluor 488-conjugated goat anti-rabbit IgG and Alexa Fluor 568-conjugated goat anti-mouse IgG (Invitrogen), were used at a 1:1000 dilution in blocking solution at 37 °C for 30 min. DAPI (4′,6-diamidino-2-phenylindole) at 2 μg/ml was added to the secondary-antibody solution to stain the nuclei. The samples were then applied to polyethyleneimine-coated coverslips. Slides were mounted in ProLong Gold Antifade reagent (Invitrogen) and viewed under a 63× oil immersion lens. High-resolution image capture and processing were performed with confocal scanning laser microscopes (LSM710 and LSM700; Carl Zeiss MicroImaging, Thornwood, NY). Images were processed by Image J (NIH).

### Fractionation of PfMSA180 expressed in the parasite

Schizont-rich parasites (~10^8^) were disrupted by sonication (10 sec pulse, 30 sec rest, repeated 10 times) in PBS supplemented with Complete proteinase inhibitor (Roche). Undisrupted cells were removed by low-speed centrifuge. The cell lysates were then fractionated by ultra-centrifugation at 60,000 × g for 1 h at 4 °C. The resulting supernatants were defined as soluble fractions, and the pellets were defined as membrane fractions. The fractions were applied to SDS-PAGE. Samples in each lane corresponded to 10^6^ cells.

### Growth inhibition assay (GIA)

Growth inhibition assays (GIA) were performed as described^46^. Briefly, total rabbit IgGs to PfMSA180, AMA1, and HisGST were purified from respective rabbit antisera using HiTrap protein G-Sepharose columns (GE Healthcare) according to the manufacturer’s protocol. As a further step, the antibodies were buffer exchanged into incomplete culture medium, concentrated with Amicon Ultra-15 centrifugal filter units (Millipore), and filter sterilized with an Ultrafree-MC GV 0.22 μm centrifugal filter (Millipore). To remove nonspecific anti-erythrocyte antibodies, the antibodies were preabsorbed with 25 μl of packed human O+ erythrocytes per purified IgG from 1 ml of antiserum on a rotating wheel for 1 hr at RT. Concentrations of all rabbit antibodies were adjusted using incomplete culture medium to a final stock concentration of 40 mg/ml.

The inhibitory activity of rabbit antibodies on merozoite invasion was tested over one cycle of parasite replication, and parasitemia was determined by flow cytometry as described^46^. Briefly, twenty microliters of a late trophozoite-to-schizont stage -infected erythrocyte (pRBC) suspension (0.3% parasitemia and 2% hematocrit), 20 μl of IgGs, and 20 μl of 2× culture medium were seeded per well on half-area flat-bottom 96-well cell culture microplates (Corning, Corning, NY) and gently mixed. For a control, 20 μl of culture medium was added to the pRBC suspension. Cultures were incubated at 37 °C in humidified airtight boxes, gassed with 90% N_2_, 5% O_2_, and 5% CO_2_. After 25 h of incubation, the pRBC were pelleted by brief centrifugation (1,300 × g for 5 min) and washed once in 100 μl PBS. The cells were then incubated with 50 μl of diluted (1:1,000 in PBS) SYBR green I (Invitrogen) for 10 min at RT and washed once in PBS. Parasitemia was measured by flow cytometry with a FACSCanto II (BD Biosciences, San Jose, CA) by the acquisition of 50,000 events per sample. Data were analyzed with FlowJo 9.1 software (Tree Star, Ashley, OR) by first gating for intact erythrocytes by side scatter and forward scatter parameters and subsequently determining the proportion of SYBR green I positive cells. Samples were tested in triplicate in each experiment, and three independent experiments were performed.

### Enzyme-linked immunosorbent assay (ELISA)

Ninety-six-well ELISA plates were coated with 50 µl of purified PfMSA180-Tr4 protein diluted to 1 µg/ml in coating buffer (20 mM boric acid, pH 8.9) and incubated at 4 °C overnight. The plates were washed with phosphate-buffered saline with 0.1% (v/v) Tween-20 (PBS-T) and then blocked with 2 mg/ml of gelatin in coating buffer for 1 h at room temperature. Human serum samples were diluted to 1:600 in PBS-T, added to antigen-coated wells in duplicate, and incubated for 1 h at RT. After washing the plates were incubated with 1:3,000-diluted HRP-conjugated rabbit anti-human IgG (Dako Cytomation, Glostrup, Denmark) in PBS-T for 1 h at room temperature. The plates were again washed with PBS-T followed by incubation for 20 min at RT with 0.5 mg/ml azino-bis-3-ethylbenthiazoline-6-sulfonic acid (Wako Pure Chemical, Osaka, Japan) diluted in citrate buffer (0.1 M citric acid, pH 4.1). The reaction was stopped with 0.1 M citric acid, and optical densities (ODs) were determined at 415 nm using a precision microplate reader (Molecular Devices, Sunnyvale, CA). Antibody levels were measured in two independent assays.

### Erythrocyte binding assay (EBA)

EBA and enzymatic treatment of RBC were performed as described^21^. In brief, 90 μl of ×20 concentrated culture supernatant of 3D7 parasite was incubated with 10 μl of untreated and enzyme-treated human erythrocytes on a rotating wheel for 60 min at RT. Erythrocyte binding was subsequently evaluated by Western blotting^21^.

### Protein-protein interactions by AlphaScreen

Interaction between PfMSA180 and 13 erythrocyte surface proteins was quantified by AlphaScreen as reported with slight modification^35,36^ (Fig. S2A). Briefly, reactions were carried out in 20 μl of reaction volumes per well in 384-well OptiPlate microtiter plates (PerkinElmer). Affinity-purified PfMSA180-Tr4 recombinant protein was biotinylated using a Biotin Labeling Kit-NH_2_ (Dojindo Molecular Technologies, Kumamoto, Japan) according to the manufacturer’s instructions. Then 5 μl of 10 nM biotinylated protein was mixed with 5 μl of 10 nM of each erythrocyte surface protein in reaction buffer (100 mM Tris-HCL [pH 8.0], 0.01% [v/v] Tween-20 and 0.1 mg/ml [w/v] bovine serum albumin) and incubated for 1 h at 26 °C to form a protein-protein complex. Subsequently, a 10 μl suspension of streptavidin-coated donor-bead and anti-GST acceptor-bead (PerkinElmer) 1:1 (v/v) mixture in the reaction buffer was added to a final concentration of 15 μg/ml of both beads. The mixture was incubated at 26 °C for 12 h in the dark to allow the donor- and acceptor-beads to optimally bind to biotin and GST, respectively. Upon illumination of this complex, a luminescence signal at 620 nm was detected using an EnVision plate reader (PerkinElmer) and the result was expressed as AlphaScreen counts. HisGST was used to set the background and was subtracted from each sample signal.

### Surface plasmon resonance (SPR)

SPR experiments were performed using a Biacore X100 instrument (GE Healthcare) according to the manufacturer’s instructions. Biacore X100 evaluation software was used for single-cycle or affinity binding analyses. Sensor CM5, amine coupling reagents, and buffers were purchased from GE Healthcare. Fresh HBS-EP+ (10 mM HEPES, pH 7.4, 150 mM NaCl, 3 mM EDTA, 0.05% (v/v) surfactant P20) was used as running buffer for all SPR experiments. Blank flow cells were used to subtract buffer effects on sensorgrams. After subtraction of the contribution of bulk refractive index, and nonspecific interactions with the CM5 chip surface, individual association (*k*_a_) and dissociation (*k*_d_) rate constants were obtained by global fitting of data. Measurement conditions were optimized so that the contribution of mass transport to the observed constants was negligible.

### Polymorphism analysis

Nucleotide sequences were downloaded from PlasmoDB (www.plasmodb.org). The sequences were aligned and analyzed using ClustalW, implemented in Mega 7^48^ and DnaSP version 6.11.

### Statistical analysis

The Kruskal-Wallis test was used to test the significance of differences in ELISA values between groups followed by the Bonferroni correction test. All analyses were performed using GraphPad Prism (GraphPad Software, San Diego, CA).

### Serum samples collection and ethical approval

Human serum samples were from a published archive from asymptomatic *P. falciparum* carriers, adults with uncomplicated symptomatic *P. falciparum* malaria, and malaria naïve individuals from Thailand as described^23^. All participant provided written informed consent. The study was approved by the Faculty of Medicine, Ramathibodi Hospital, Mahidol University (ID: 09-46-10), Ethics Committee of the Thai Ministry of Public Health and the Institutional Review Board of the Walter Reed Army Institute of Research (WRAIR 802). Protocol to use the samples in Japan was approved by Institutional Review Boards of Ehime University Hospital^23^.

All methods were performed in accordance with the relevant guidelines and regulations.

## Supplementary information

Supplementary Table 1 and Figure 1-3

Supplementary data file 1

Supplementary data file 2

## Data Availability

The datasets generated and analyzed during the current study are available from the corresponding author on reasonable request.

## References

[1] WHO. World Malaria Report 2016. 186 (World Health Organization, Geneva, Switzerland, Geneva, Switzerland, 2016).

[2] Hsieh FL (2016). The structural basis for CD36 binding by the malaria parasite. Nat Commun.

[3] N’Guessan R, Corbel V, Akogbéto M, Rowland M (2007). Reduced efficacy of insecticide-treated nets and indoor residual spraying for malaria control in pyrethroid resistance area, Benin. Emerg Infect Dis.

[4] Hoffman SL, Vekemans J, Richie TL, Duffy PE (2015). The march toward malaria vaccines. Vaccine.

[5] Dvorak JA, Miller LH, Whitehouse WC, Shiroishi T (1975). Invasion of erythrocytes by malaria merozoites. Science.

[6] Cowman AF, Tonkin CJ, Tham WH, Duraisingh MT (2017). The Molecular Basis of Erythrocyte Invasion by Malaria Parasites. Cell Host Microbe.

[7] Cowman AF, Berry D, Baum J (2012). The cellular and molecular basis for malaria parasite invasion of the human red blood cell. J Cell Biol.

[8] Beeson JG (2016). Merozoite surface proteins in red blood cell invasion, immunity and vaccines against malaria. FEMS Microbiol Rev.

[9] Sirima SB (2016). A phase 2b randomized, controlled trial of the efficacy of the GMZ2 malaria vaccine in African children. Vaccine.

[10] Sagara I (2009). A randomized controlled phase 2 trial of the blood stage AMA1-C1/Alhydrogel malaria vaccine in children in Mali. Vaccine.

[11] Ogutu BR (2009). Blood stage malaria vaccine eliciting high antigen-specific antibody concentrations confers no protection to young children in Western Kenya. PLoS One.

[12] Riley EM, Stewart VA (2013). Immune mechanisms in malaria: new insights in vaccine development. Nat Med.

[13] Takala SL (2009). Extreme polymorphism in a vaccine antigen and risk of clinical malaria: implications for vaccine development. Sci Transl Med.

[14] Neafsey DE (2015). Genetic Diversity and Protective Efficacy of the RTS,S/AS01 Malaria Vaccine. N Engl J Med.

[15] Conway DJ (2015). Paths to a malaria vaccine illuminated by parasite genomics. Trends Genet.

[16] Proietti C, Doolan DL (2014). The case for a rational genome-based vaccine against malaria. Front Microbiol.

[17] Dreyer AM (2012). Passive immunoprotection of Plasmodium falciparum-infected mice designates the CyRPA as candidate malaria vaccine antigen. J Immunol.

[18] Baum J (2009). Reticulocyte-binding protein homologue 5 - an essential adhesin involved in invasion of human erythrocytes by Plasmodium falciparum. Int J Parasitol.

[19] Chen L (2011). An EGF-like protein forms a complex with PfRh5 and is required for invasion of human erythrocytes by Plasmodium falciparum. PLoS Pathog.

[20] Bergmann-Leitner ES (2010). Immunization with pre-erythrocytic antigen CelTOS from Plasmodium falciparum elicits cross-species protection against heterologous challenge with Plasmodium berghei. PLoS One.

[21] Arumugam TU (2011). Discovery of GAMA, a Plasmodium falciparum merozoite micronemal protein, as a novel blood-stage vaccine candidate antigen. Infect Immun.

[22] Olugbile S (2011). Malaria vaccine candidate: design of a multivalent subunit α-helical coiled coil poly-epitope. Vaccine.

[23] Sakamoto H (2018). Identification of target proteins of clinical immunity to Plasmodium falciparum in a region of low malaria transmission. Parasitol. Int..

[24] Tsuboi T, Takeo S, Arumugam TU, Otsuki H, Torii M (2010). The wheat germ cell-free protein synthesis system: a key tool for novel malaria vaccine candidate discovery. Acta Trop.

[25] Koussis K (2009). A multifunctional serine protease primes the malaria parasite for red blood cell invasion. EMBO J.

[26] Bunnik EM (2013). Polysome profiling reveals translational control of gene expression in the human malaria parasite Plasmodium falciparum. Genome Biol.

[27] Muh F (2017). Identification of a novel merozoite surface antigen of Plasmodium vivax, PvMSA180. Malar J.

[28] Kadekoppala M, Ogun SA, Howell S, Gunaratne RS, Holder AA (2010). Systematic genetic analysis of the Plasmodium falciparum MSP7-like family reveals differences in protein expression, location, and importance in asexual growth of the blood-stage parasite. Eukaryot Cell.

[29] Howell SA, Withers-Martinez C, Kocken CH, Thomas AW, Blackman MJ (2001). Proteolytic processing and primary structure of Plasmodium falciparum apical membrane antigen-1. J Biol Chem.

[30] Reddy KS (2015). Multiprotein complex between the GPI-anchored CyRPA with PfRH5 and PfRipr is crucial for Plasmodium falciparum erythrocyte invasion. Proc Natl Acad Sci USA.

[31] Sahar T (2011). Plasmodium falciparum reticulocyte binding-like homologue protein 2 (PfRH2) is a key adhesive molecule involved in erythrocyte invasion. PLoS One.

[32] Reddy KS (2014). Bacterially expressed full-length recombinant Plasmodium falciparum RH5 protein binds erythrocytes and elicits potent strain-transcending parasite-neutralizing antibodies. Infect Immun.

[33] Sim BK, Chitnis CE, Wasniowska K, Hadley TJ, Miller LH (1994). Receptor and ligand domains for invasion of erythrocytes by Plasmodium falciparum. Science.

[34] Bartholdson SJ, Crosnier C, Bustamante LY, Rayner JC, Wright GJ (2013). Identifying novel Plasmodium falciparum erythrocyte invasion receptors using systematic extracellular protein interaction screens. Cell Microbiol.

[35] Matsuoka K, Komori H, Nose M, Endo Y, Sawasaki T (2010). Simple screening method for autoantigen proteins using the N-terminal biotinylated protein library produced by wheat cell-free synthesis. J Proteome Res.

[36] Kanoi BN (2017). Antibody profiles to wheat germ cell-free system synthesized Plasmodium falciparum proteins correlate with protection from symptomatic malaria in Uganda. Vaccine.

[37] Morita M (2017). Immunoscreening of Plasmodium falciparum proteins expressed in a wheat germ cell-free system reveals a novel malaria vaccine candidate. Sci Rep.

[38] Paul AS, Egan ES, Duraisingh MT (2015). Host-parasite interactions that guide red blood cell invasion by malaria parasites. Curr Opin Hematol.

[39] Zhang, M. *et al*. Uncovering the essential genes of the human malaria parasite. *Science* **360**, 10.1126/science.aap7847 (2018). 10.1126/science.aap7847 PMC6360947 29724925

[40] Silmon de Monerri NC (2011). Global identification of multiple substrates for Plasmodium falciparum SUB1, an essential malarial processing protease. Infect Immun.

[41] Lindberg FP, Gresham HD, Schwarz E, Brown EJ (1993). Molecular cloning of integrin-associated protein: an immunoglobulin family member with multiple membrane-spanning domains implicated in alpha v beta 3-dependent ligand binding. J Cell Biol.

[42] Oldenborg PA (2000). Role of CD47 as a marker of self on red blood cells. Science.

[43] Banerjee R, Khandelwal S, Kozakai Y, Sahu B, Kumar S (2015). CD47 regulates the phagocytic clearance and replication of the Plasmodium yoelii malaria parasite. Proc Natl Acad Sci USA.

[44] Ayi K (2016). CD47-SIRPα Interactions Regulate Macrophage Uptake of Plasmodium falciparum-Infected Erythrocytes and Clearance of Malaria *In Vivo*. Infect Immun.

[45] Tsuboi T, Takashima E (2015). Antibody titre as a surrogate of protection of the first malaria subunit vaccine, RTS,S/AS01. Lancet Infect Dis.

[46] Ito D (2013). RALP1 is a rhoptry neck erythrocyte-binding protein of Plasmodium falciparum merozoites and a potential blood-stage vaccine candidate antigen. Infect Immun.

[47] Trager W, Jensen JB (1976). Human malaria parasites in continuous culture. Science.

[48] Kumar S, Stecher G, Tamura K (2016). MEGA7: Molecular Evolutionary Genetics Analysis Version 7.0 for Bigger Datasets. Mol Biol Evol.

[49] Ito D (2011). Plasmodial ortholog of Toxoplasma gondii rhoptry neck protein 3 is localized to the rhoptry body. Parasitol Int.

