# Supplementary material for: PfMSA180 is a novel Plasmodium falciparum vaccine antigen that interacts with human erythrocyte integrin associated protein (CD47)
Source: Sci Rep. 2019 Apr 11;9:5923. doi: 10.1038/s41598-019-42366-9 (PMC6459815; doi:10.1038/s41598-019-42366-9)
Supplement: Supplementary file 1 — Supplementary Table 1 and Figure 1-3 [file 41598_2019_42366_MOESM1_ESM.pdf]

## Supplementary Table and Figures

# PfMSA180 is a novel *Plasmodium falciparum* vaccine antigen that interacts with human erythrocyte integrin associated protein (CD47)

Hikaru Nagaoka[1], Chisa Sasaoka[1], Takaaki Yuguchi[1], Bernard N. Kanoi[1], Daisuke Ito[2], Masayuki Morita[1], Rachanee Udomsangpetch[3], Jetsumon Sattabongkot[4], Tomoko Ishino[5], Takafumi Tsuboi[1], Eizo Takashima[1,*]

[1] Division of Malaria Research, Proteo-Science Center, Ehime University, 3 Bunkyo-cho, Matsuyama, Ehime 790-8577, Japan;
[2] Division of Medical Zoology, Department of Microbiology and Immunology, Faculty of Medicine, Tottori University, 86 Nishi-cho, Yonago, Tottori 683-8503, Japan;
[3] Center for Research and Innovation, Faculty of Medical Technology, Mahidol University, Salaya, Nakhosn Pathom 73170, Thailand;
[4] Mahidol Vivax Research Unit, Faculty of Tropical Medicine, Mahidol University, Bangkok 10400, Thailand;
[5] Division of Molecular Parasitology, Proteo-Science Center, Ehime University, Toon Ehime 791-0295, Japan

* Corresponding author
Address: Division of Malaria Research, Proteo-Science Center, Ehime University, Japan, 3 Bunkyo-cho, Matsuyama, Ehime 790-8577, Japan. Tel.: (+81) 89 927 9939. E-mail address: takashima.eizo.mz@ehime-u.ac.jp

# Table S1: List of primers used in this study

| Protein | Primer sequence | Target amino acid range |
|---|---|---|
| PfMAS170Tr1-F | CTCGAGGAAAATGTTAATAATAAAAACTGTAATGAG | |
| PfMAS170Tr1-R | GCGGCCGCCTATGATACATACACATTATCCTCTTCAG | E22 – S263 |
| PfMAS170Tr2-F | CTCGAGGCTACAAAAGGAAATCAAAAAGAAGAAACTG | |
| PfMAS170Tr2-R | GCGGCCGCTACATCAATTACGTCTACTTTTGTTTGTA | A264 – D501 |
| PfMAS170Tr3-F | CTCGAGATTGCAAATACTATTTATGTAAATGTTGG | |
| PfMAS170Tr3-R | GCGGCCGCTATGGGAAATATCTAGAATTATGATAATATGC | I508 – P723 |
| PfMAS170Tr4-F | CTCGAGGCAAAAAATTTTTATAACATATCTAATGAG | |
| PfMAS170Tr4-R | GCGGCCGCTATGGATATTGATTATTATTTGTTTGTTCA | A805 – P1093 |
| PfMAS170Tr5-F | CTCGAGCTCTTTGATACTGATAAAAAAAGAAATTAT | |
| PfMAS170Tr5-R | GCGGCCGCTACTATGGATTTCTAAAATCTAGTGCATC | L1193 – P1455 |
| AMIGO2_F | GGGCGGATATCTCGAATGTCGTTACGTGTACACAC | |
| AMIGO2_R | AAAACTAGTGCGGCCCTAATGGTGATGGTGATGGTGGTTAAATGCCTCATGAGCAT | M1 - N397 |
| BCAM_F | GGGCGGATATCTCGAGAGGTGCGCTTGTCTGTA | |
| BCAM_R | AAAACTAGTGCGGCCCTAATGGTGATGGTGATGGTGGCCACGGCCATGACGGCC | E32 – A555 |
| CD44_F | GGGCGGATATCTCGACAGATCGATTTGAATATAACC | |
| CD44_R | AAAACTAGTGCGGCCCTAATGGTGATGGTGATGGTGTTCTGGAATTTGGGGTGTC | Q21 – E606 |
| CD47_F | GGGCGGATATCTCGAAAAACAAAATCTGTAGAATTCAC | |
| CD47_R | AAAACTAGTGCGGCCCTAATGGTGATGGTGATGGTGTGGAGAAAACCATGAAACAAC | K24 – S139 |
| CD55_F | GGGCGGATATCTCGAGACTGTGGCCTTCCCCCA | |
| CD55_R | AAAACTAGTGCGGCCCTAATGGTGATGGTGATGGTGAGTCAGCAAGCCCATGGT | D35 – T381 |
| CD58_F | GGGCGGATATCTCGATCCCAACAAATATATGGTGTT | |
| CD58_R | AAAACTAGTGCGGCCCTAATGGTGATGGTGATGGTGTCTGTGTCTTGAATGACCG | S30 – R215 |
| CD59_F | GGGCGGATATCTCGACTGCAGTGCTACAACTGTC | |
| CD59_R | AAAACTAGTGCGGCCCTAATGGTGATGGTGATGGTGGGGATGAAGGCTCCAGGC | L26 – P128 |
| CD99_F | GGGCGGATATCTCGAGATTTATCTGATGCCCTTCC | |
| CD99_R | AAAACTAGTGCGGCCCTAATGGTGATGGTGATGGTGGCCTGGGGCGTCGGCCTC | D27 – G125 |
| ERMAP_F | GGGCGGATATCTCGACACGCAGGGGATGCCGGC | |
| ERMAP_R | AAAACTAGTGCGGCCCTAATGGTGATGGTGATGGTGTGAGGGGGAGAGACTCCC | H30 – S154 |
| F11-Receptor_F | GGGCGGATATCTCGACACTCTTCTGAACCTGAAG | |
| F11-Receptor_R | AAAACTAGTGCGGCCCTAATGGTGATGGTGATGGTGGACCCCCACATTCCGCTC | H32 – V238 |
| ICAM4_F | GGGCGGATATCTCGAGCGCTGGGACGCCGGACT | |
| ICAM4_R | AAAACTAGTGCGGCCCTAATGGTGATGGTGATGGTGGCCACCATGTATGGCCAT | A23 – G272 |
| NPTN_F | GGGCGGATATCTCGACAGAACGAGCCAAGGATTG | |
| NPTN_R | AAAACTAGTGCGGCCCTAATGGTGATGGTGATGGTGCAGGTGGCTCCGCACCCT | Q29 – L221 |
| SEMA7A_F | GGGCGGATATCTCGACAGGGCCACCTAAGGAGC | |
| SEMA7A_R | AAAACTAGTGCGGCCCTAATGGTGATGGTGATGGTGGTGGACCAGCAAGCCAAG | Q45 – H666 |

# Fig S1

a

MNRIFYFCLFTILFWLSLVSGENVNNKNCNEKNRKAILLALLKNSLVDNKDYNNSEELKYALEHIQNSELYPKDSKKFDKFIDEFFSYYNIHVNFTDEEKRILHISGVFKEFY
VDVDNLNKDEMKEYFKKNYEKGLSLINLIVHSNLIIQQFDHDIIDKKKVHEQNTNTNKTLEYISDNLNDLINFKNIHLNNNSTGDFIIKLYTNYVNYINPYQTNPLPNTPHYYE
HKNFHTKEHYIYDEEIVNPNMDNINTHTEEDNVYVSATKGNQKEETEKKENHENNNAINPKYMNYETYYKKIFNAIFEQIDKLNKTLFEIKNKNNSETNENISESNSGNPEL
NNENSYSVKLSSSSPNSTNKESLIFPYTYYNPYYMFRLTNNFKENDEGLKNENNINNNEDNQNDNMNIVLGKIHNILKDFNINENIMTNKMSAPLIMTIILNFFKKYMAENK
FNLPMSSEVENKINKSNNKALLQQSNKDTPIHKKKEIRNKKIQTKVDVIDEKTKKKIANTIYVNVGQSGINGFFNFFDFREKSIDSNIFDLLHVMEDMKIFDIFQTIIFIQKFTE
NVCASYCMNITDVLELSHYDMIFYDKMVFHFSKDGMMIKTDKKYLYNLKEFENILNLLNINANTIALNCTCKFYVDVNYTYSEQYKMHLKGYLHKMNEFDYINNFSASYLL
NQLIIFQDKFNYIKMNGKLPIDDPKNIYNMNNVHDTAYYHNSRYFPTKDMPSLEDNFYEHLKYPDINTIHIYYNASPVKLNEVNDLKTIIIDEIKSKIFYINSYRVGDQFFPTYS
NLGKDDHDLEHSAKNFYNISNENGDNTFNNNNNNMDNKKRMYNYNKHKDNDSRYTDNSNKNRDNSNKNRDNYNRNKDKNNTNRDNYNRYKDNNYYYNNSDNNN
YNERKRYIRKKTYNKLSYFNLPSLKSIYNNKIKGNSEEFSFDNELPEQTESFPLNKPQDHEAFYNLKKHHTNVYEPNDEEKQNEQKLKDQIKITSDILYKDIEENKNTEDVL
LIETITINNGTTSNTIENNKDSNKEAENSNTEQNDNNNNDNNNNINNNNNNNDNKEEDMNENNNNSKVTGDSVENINEQTNNNQYPNTEYNTIQRSINAKYLIFFFKNLH
VWKTDLFCQNINYMNNYLNSIQYNKTLTFDINYDTNAVTIYFTDNITYTVKVNLEYLVFLLEKISLITFVEDLCSLFDTDKKRNYKNLTEFLENTERINTFVRNHMMLSNEQFI
NKNKYAKELAEISTSNLFYPKKDIILRSTPYNNIILDEKDIYQTIFIYMDDMLTEKMVNDTWITPYAFVVYSKSKKDMQGNNIKIEQNKNITKYSRSAIDKYVHYEYKRISENLN
RFFMESNSNAPQFNENYKEYTIIIYNDNPSMPNIVLTTTINVFNVSFLQSIMEMLLNIKANQQFFSYKGKFIPINAFITLENKINYIFFNYIPLENYVNNGDALDFRNP

b

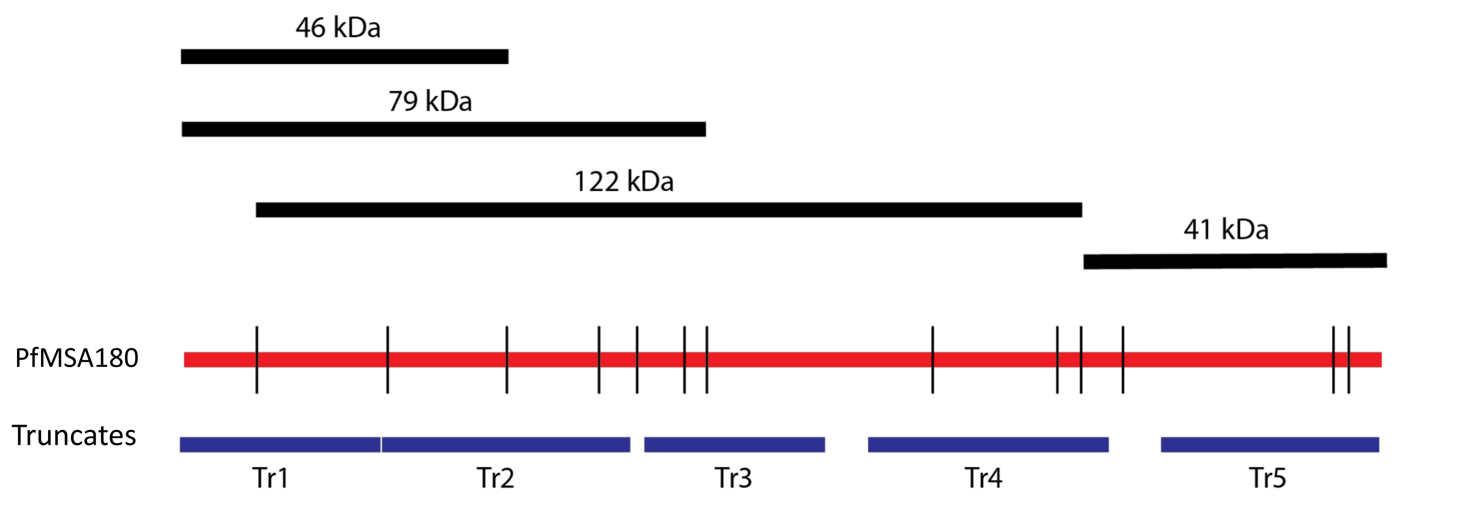


**Figure S1:** SUB1 recognition sites (Ile/Leu/Val/Thr-Xaa-Gly/Ala-Paa (not Leu) ; Xaa) identified by previous study [1] were used for prediction of PfSUB1 sites in PfMSA180 amino acid sequence. a) The amino acid sequence of PfMSA180. The predicted PfSUB1 sites are highlighted. b) Schematic presentation of the predicted PfSUB1 cleavage sites in PfMSA180 (vertical lines). Blue lines present the position of the truncates indicated. Black horizontal lines indicate predicted products due to the partial digestion of PfMSA180.


1. Withers-Martinez, Chrislaine, et al. "Plasmodium subtilisin-like protease 1 (SUB1): insights into the active-site structure, specificity and function of a pan-malaria drug target." *International journal for parasitology* 42.6 (2012): 597-612.

# Fig S2 A

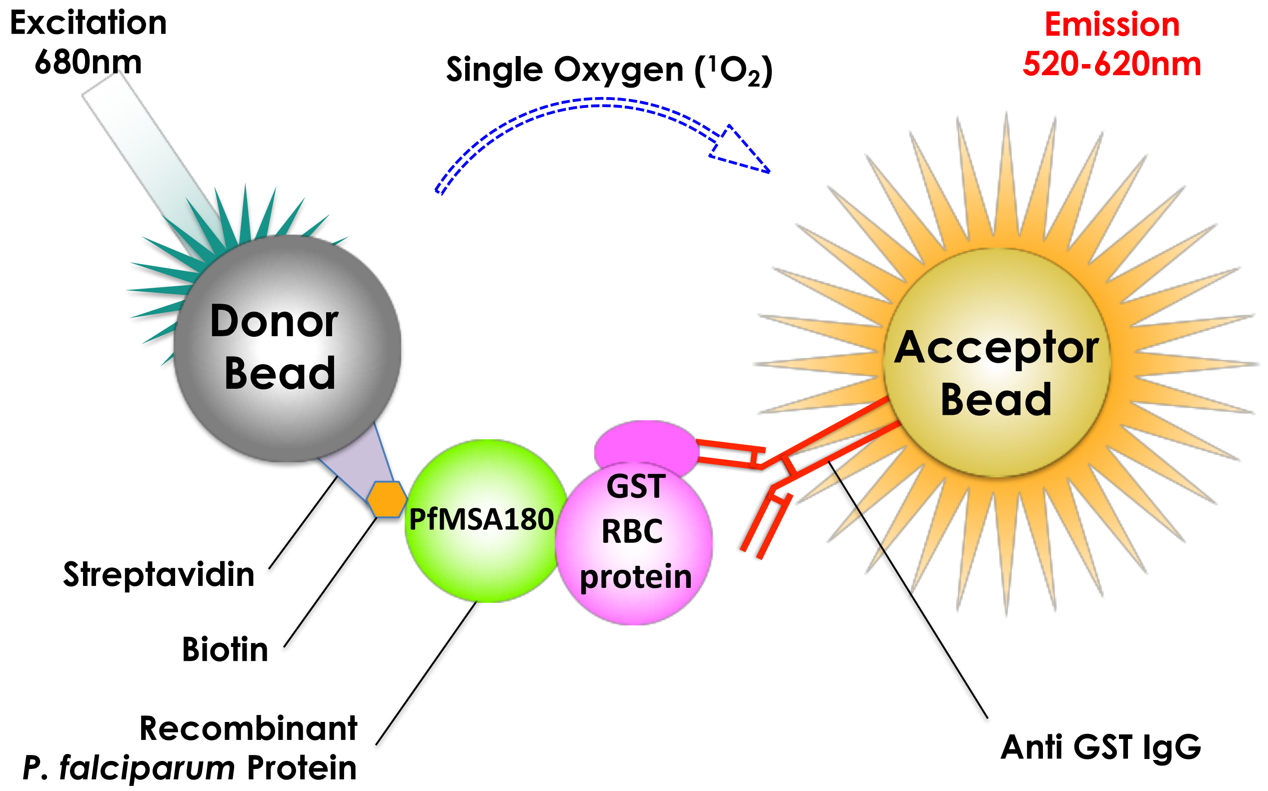


**Figure S2A: Schematic representation of AlphaScreen protein-protein interaction assay.**
GST tagged erythrocyte proteins were incubated with mono-biotinylated recombinant PfMSA180 After incubation, the streptavidin-conjugated donor beads and protein G conjugated acceptor beads – with a pre-attached anti-GST antibody were added to the mixture. If ligand-receptor complex is formed, it brings the beads into close proximity. Thus, excitation of the donor beads causes release of singlet oxygen molecules that trigger energy transfer and subsequent luminescent light emission at 520-620 nm from the acceptor beads. This reaction occurs only when the distance between donor and acceptor beads is within 200 nm.

# Fig S2B

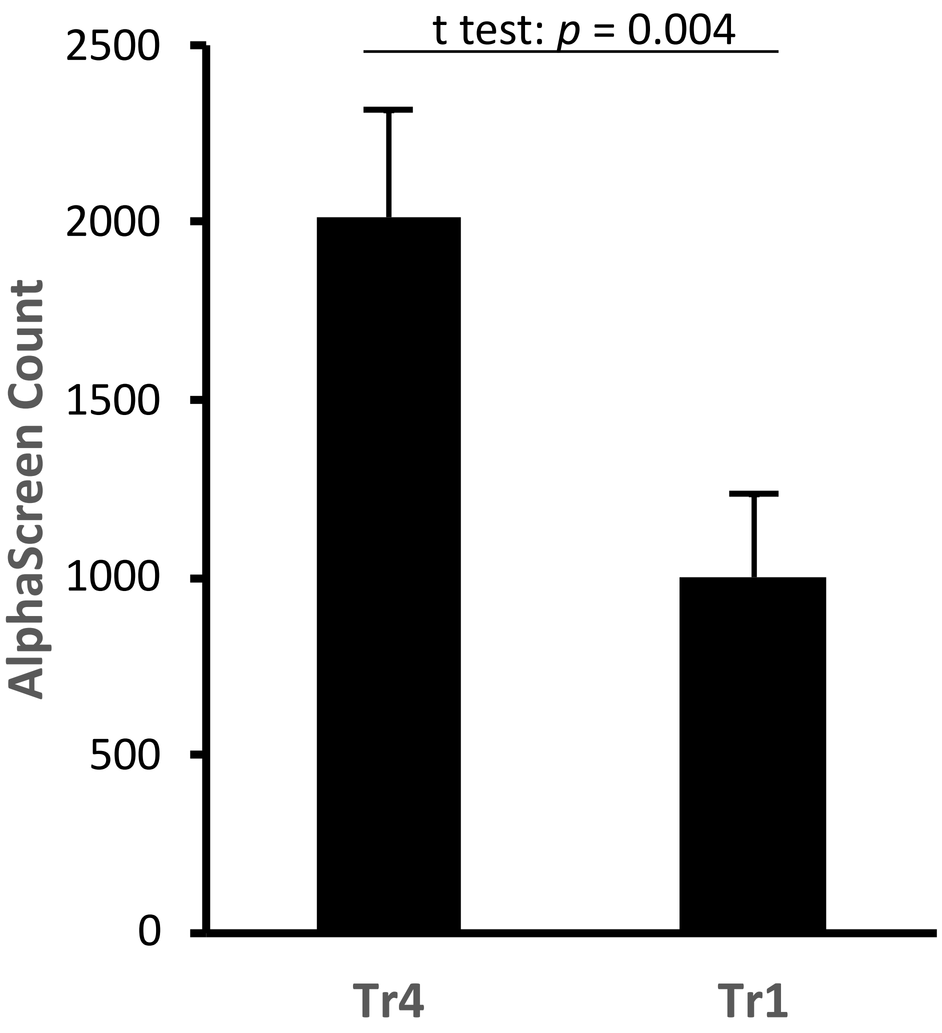


**Figure S2B:** AlphaScreen reactivity of recombinant PfMSA180-Tr4 and Tr1 to GST-fuse CD47. The AlphaScreen count was significantly higher for Tr4 compared to Tr1 (t test, $p$ = 0.004). Data represents mean of a single assay performed in triplicate. Error bars represent standard error of the mean.

# Fig S3

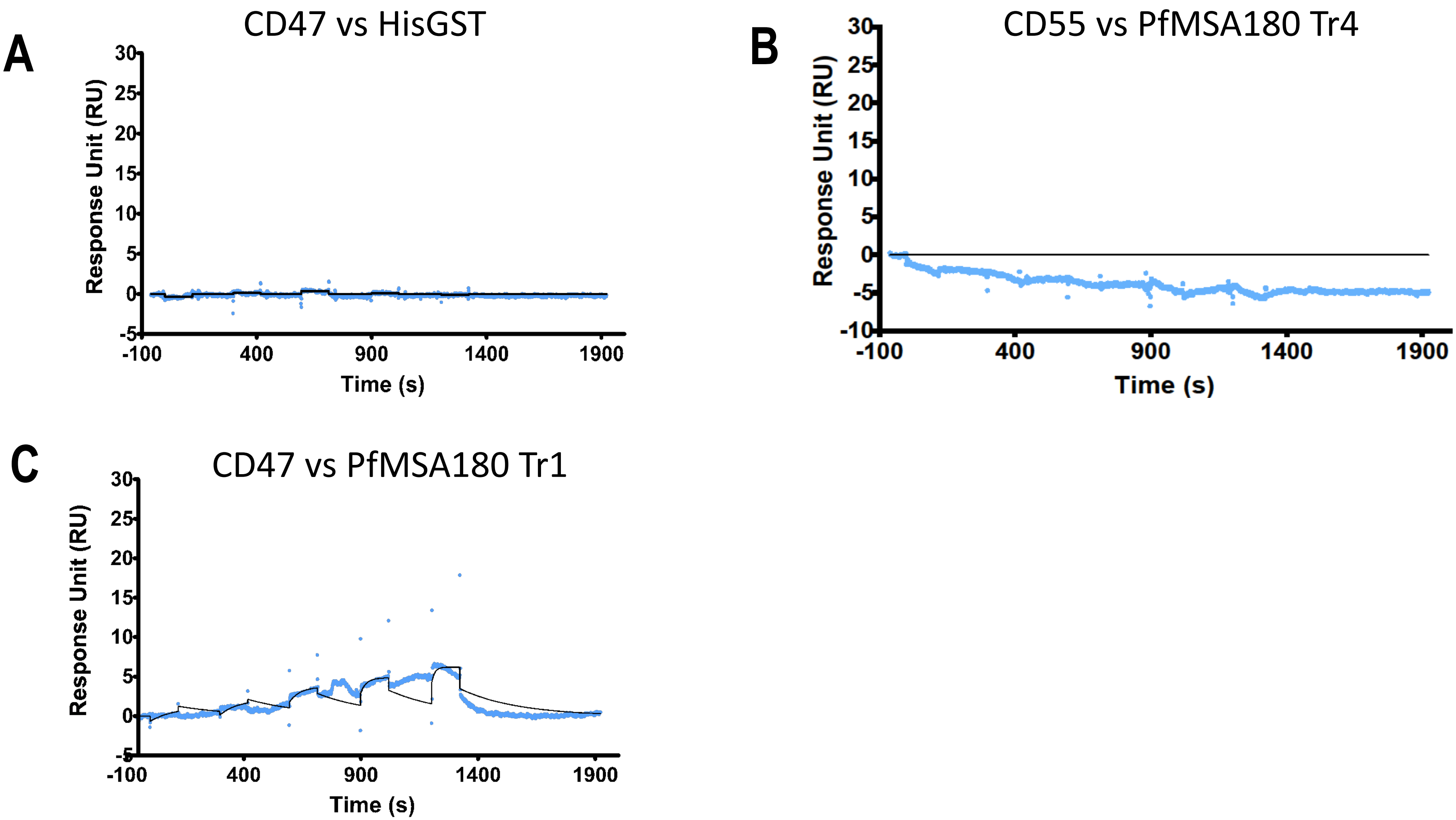


**Figure S3:** Sensorgram of SPR single-cycle kinetic analysis. Recombinant PfMSA180-Tr4 was immobilized on CM5 chip and used as the ligand while recombinant CD47 was used as analyte. Blue curve represents the actual data-generated sensorgram while black curve indicates line of fit used to calculate kinetics parameters. All assays were performed at an increasing protein concentration of 0.125, 0.25, 0.5, 1 and 2 µM at 120 s contact time and 180 s dissociation time. The dissociation time in the last cycle was extended to 580 s. A. CD47 (ligand) vs HisGST (analyte) B. CD55 (ligand) vs PfMSA180 Tr4 C. CD47 (ligand) vs PfMSA180 Tr1.
