# Supplementary material for: PfMSA180 is a novel Plasmodium falciparum vaccine antigen that interacts with human erythrocyte integrin associated protein (CD47)
Source: Sci Rep. 2019 Apr 11;9:5923. doi: 10.1038/s41598-019-42366-9 (PMC6459815; doi:10.1038/s41598-019-42366-9)
Supplement: Supplementary file 2 — Supplementary data file 1 [file 41598_2019_42366_MOESM2_ESM.pdf]

SUPPLEMENTARY DATA FILE 1

PfMSA180 is a novel *Plasmodium falciparum* vaccine antigen that interacts with human erythrocyte integrin associated protein (CD47)

Hikaru Nagaoka[1], Chisa Sasaoka[1], Takaaki Yuguchi[1], Bernard N. Kanoi[1], Daisuke Ito[2], Masayuki Morita[1], Rachanee Udomsangpetch[3], Jetsumon Sattabongkot[4], Tomoko Ishino[5], Takafumi Tsuboi[1], Eizo Takashima[1,*]

[1] Division of Malaria Research, Proteo-Science Center, Ehime University, 3 Bunkyo-cho, Matsuyama, Ehime 790-8577, Japan;
[2] Division of Medical Zoology, Department of Microbiology and Immunology, Faculty of Medicine, Tottori University, 86 Nishi-cho, Yonago, Tottori 683-8503, Japan;
[3] Center for Research and Innovation, Faculty of Medical Technology, Mahidol University, Salaya, Nakhosn Pathom 73170, Thailand;
[4] Mahidol Vivax Research Unit, Faculty of Tropical Medicine, Mahidol University, Bangkok 10400, Thailand;
[5] Division of Molecular Parasitology, Proteo-Science Center, Ehime University, Toon Ehime 791-0295, Japan

* Corresponding author
Address: Division of Malaria Research, Proteo-Science Center, Ehime University, Japan, 3 Bunkyo-cho, Matsuyama, Ehime 790-8577, Japan. Tel.: (+81) 89 927 9939. E-mail address: takashima.eizo.mz@ehime-u.ac.jp

Fig 1

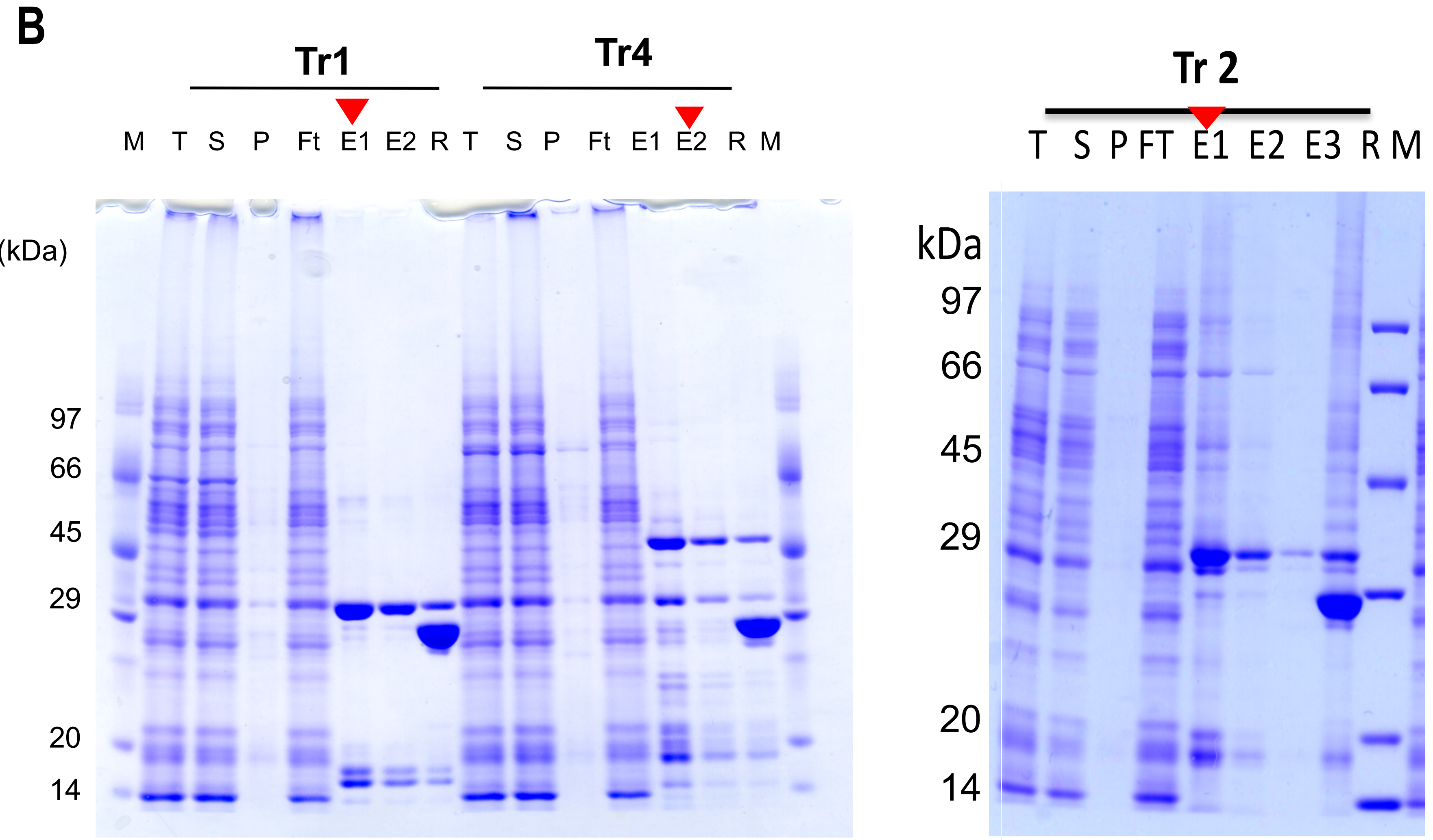

M: marker
T: total translation mix
S: supernatant
P: pellet
E1: elution 1
E2: Elution 2
Ft: flow through
R: resin

▼ Presented in the main manuscript

Fig 1

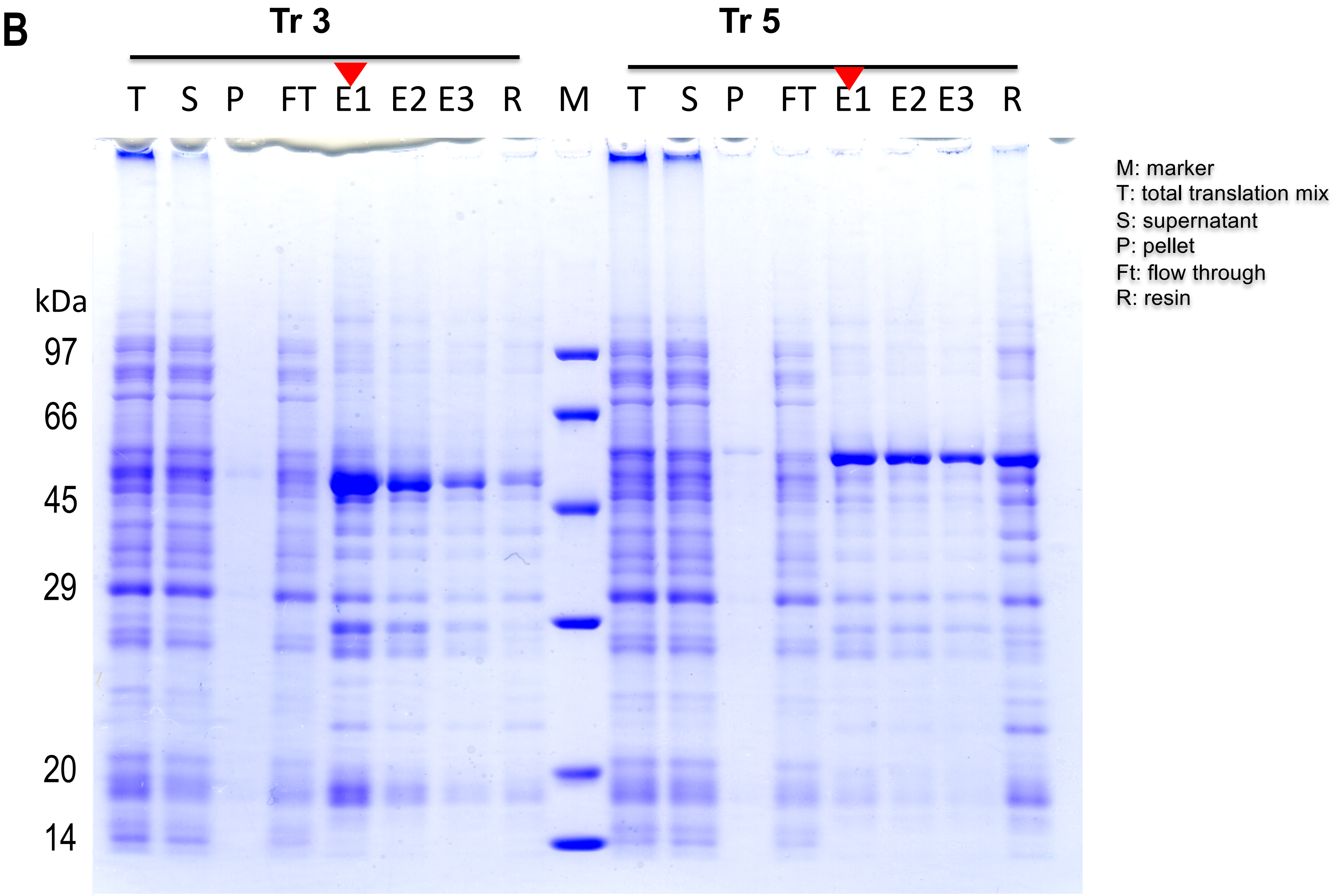

▼ Presented in the main manuscript

Fig 1

C

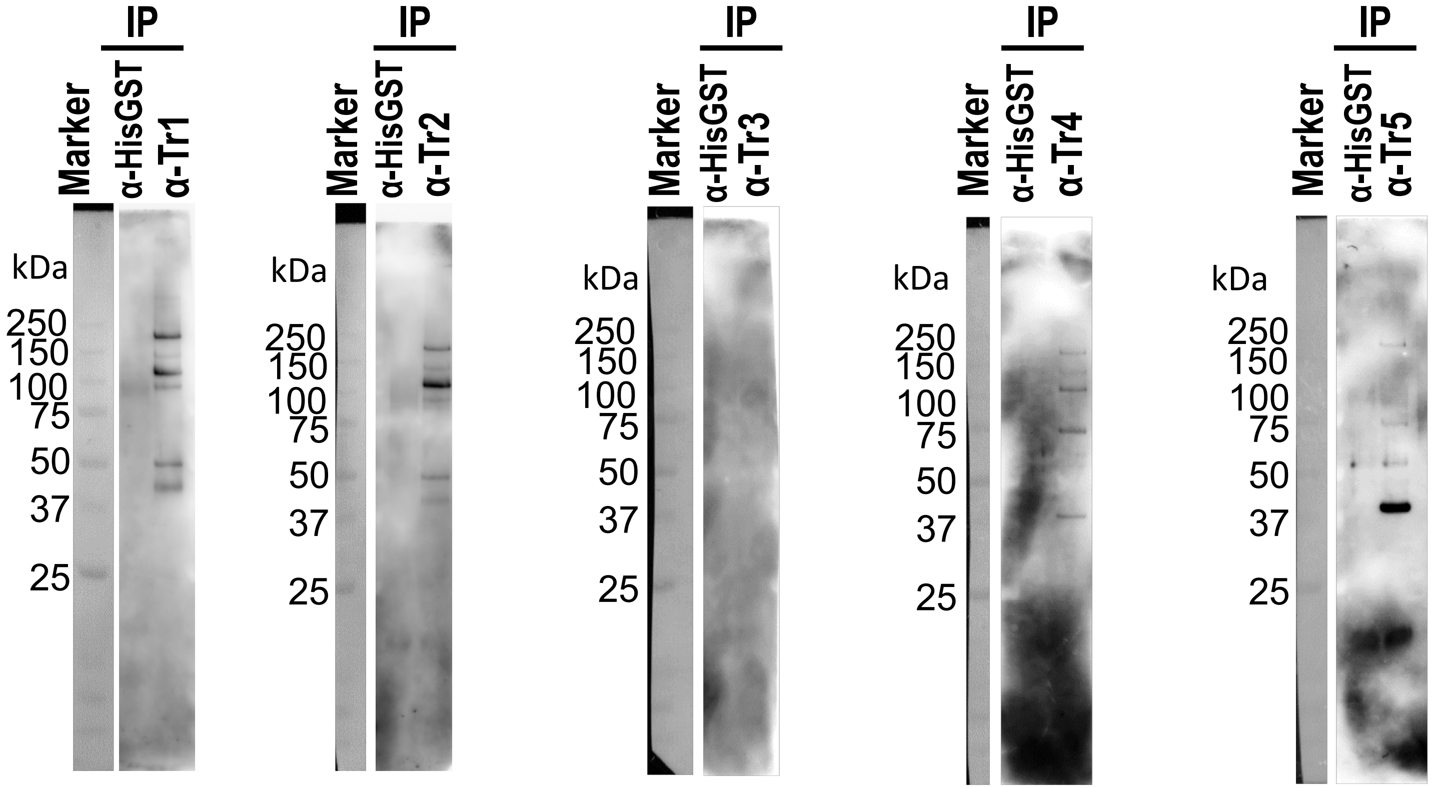
IP
Marker
α-HisGST
α-Tr1
α-Tr2
α-Tr3
α-Tr4
α-Tr5
kDa
250
150
100
75
50
37
25

Fig 2

A

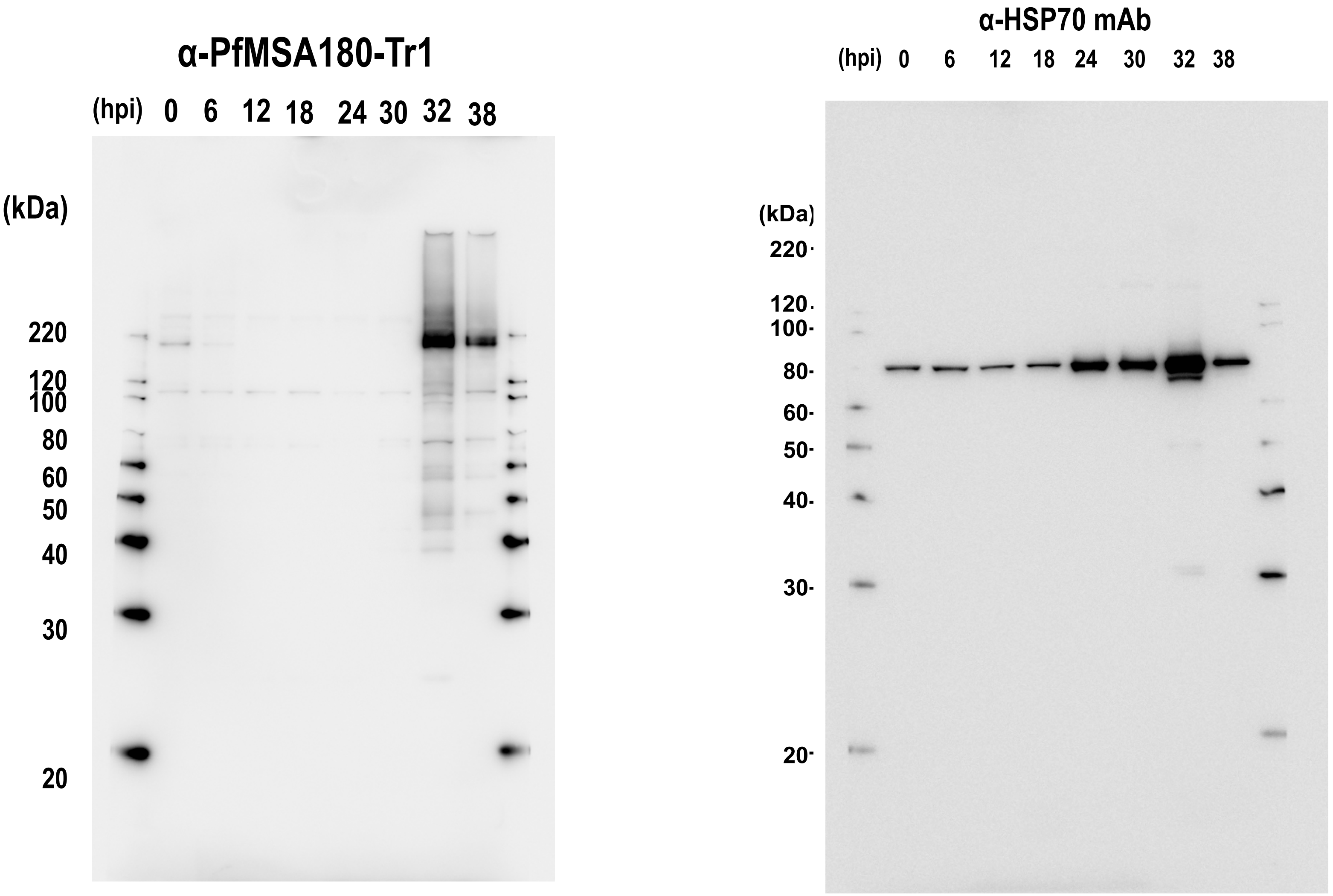
α-PfMSA180-Tr1
(hpi) 0 6 12 18 24 30 32 38
(kDa)
220
120
100
80
60
50
40
30
20
α-HSP70 mAb
(hpi) 0 6 12 18 24 30 32 38
(kDa)
220
120
100
80
60
50
40
30
20

Fig 2

A

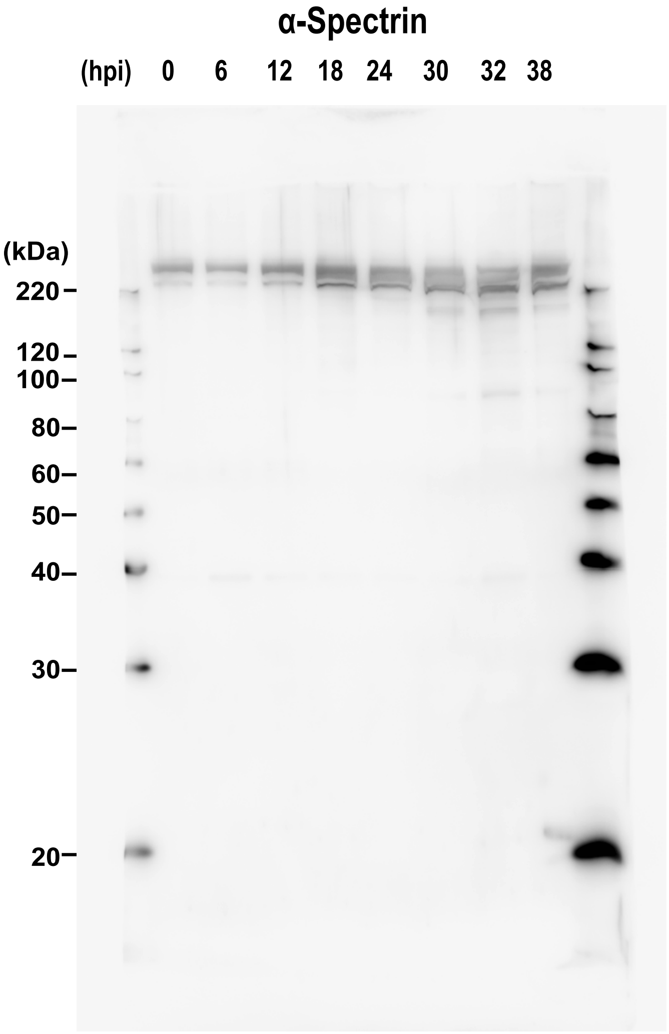
α-Spectrin
(hpi) 0 6 12 18 24 30 32 38
(kDa)
220
120
100
80
60
50
40
30
20

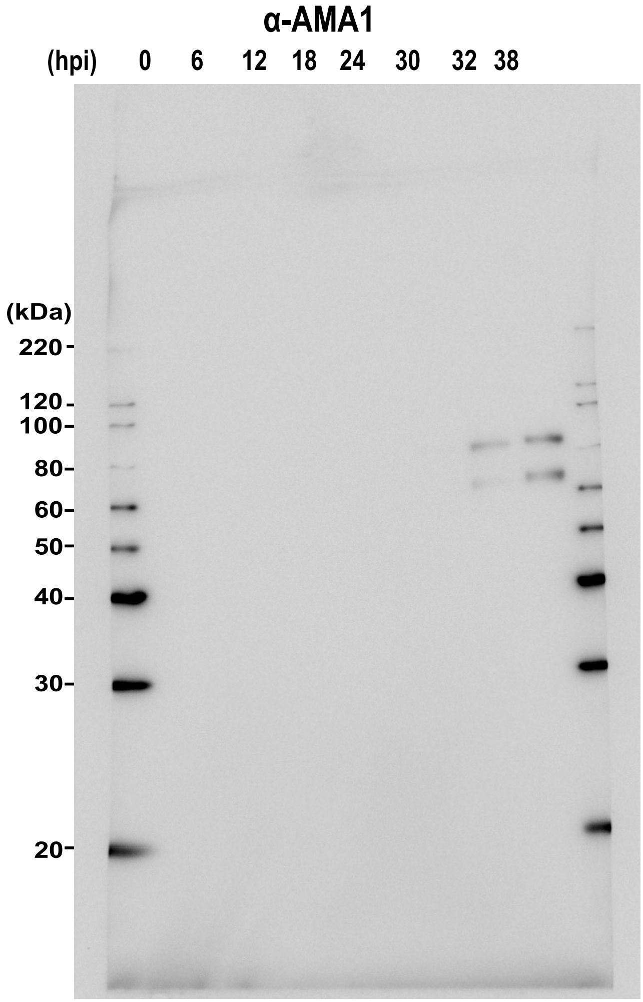
α-AMA1
(hpi) 0 6 12 18 24 30 32 38
(kDa)
220
120
100
80
60
50
40
30
20

Fig 2

D

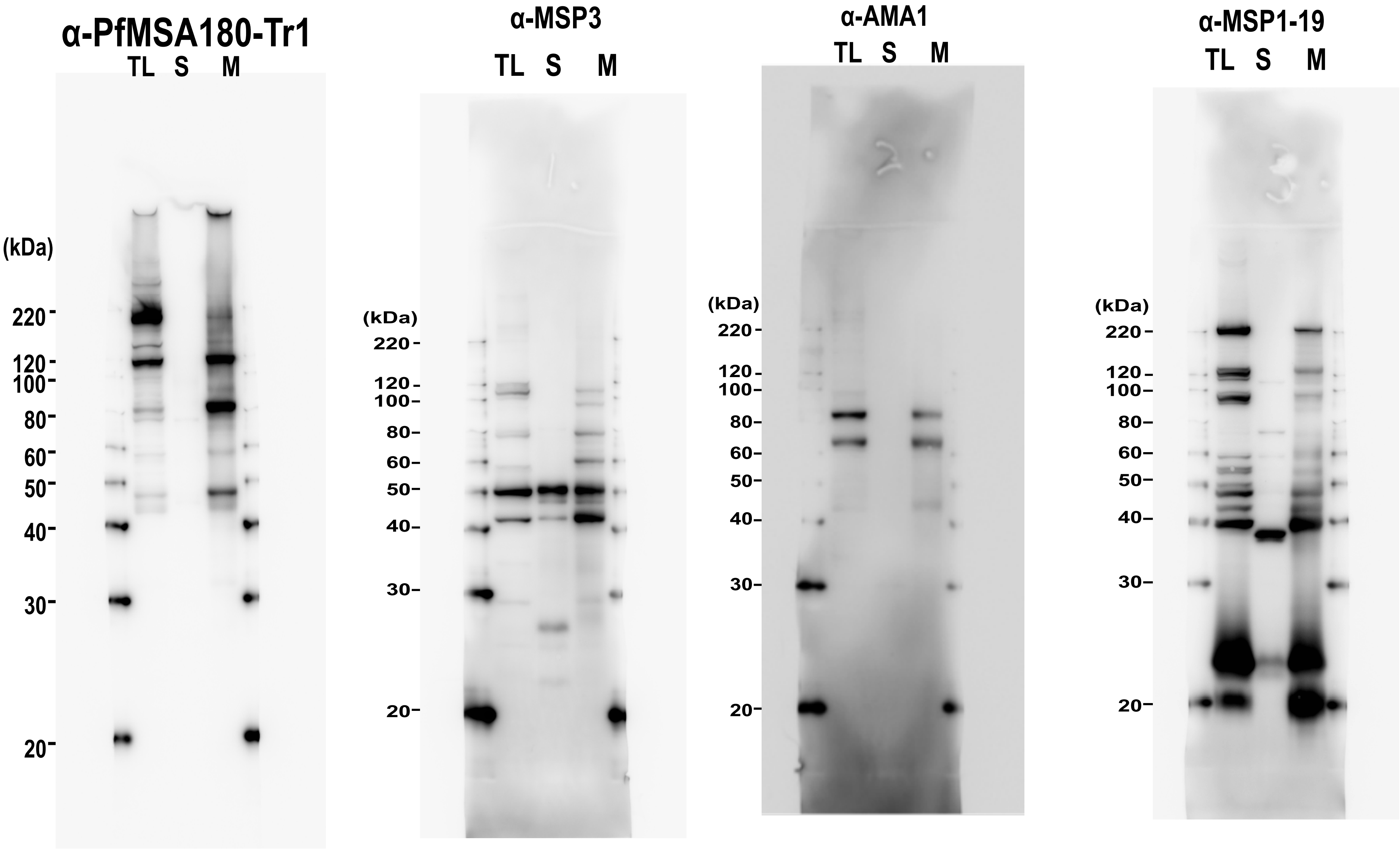
α-PfMSA180-Tr1
TL S M
(kDa)
220
120
100
80
60
50
40
30
20
α-MSP3
TL S M
(kDa)
220
120
100
80
60
50
40
30
20
α-AMA1
TL S M
(kDa)
220
120
100
80
60
50
40
30
20
α-MSP1-19
TL S M
(kDa)
220
120
100
80
60
50
40
30
20

Fig 4

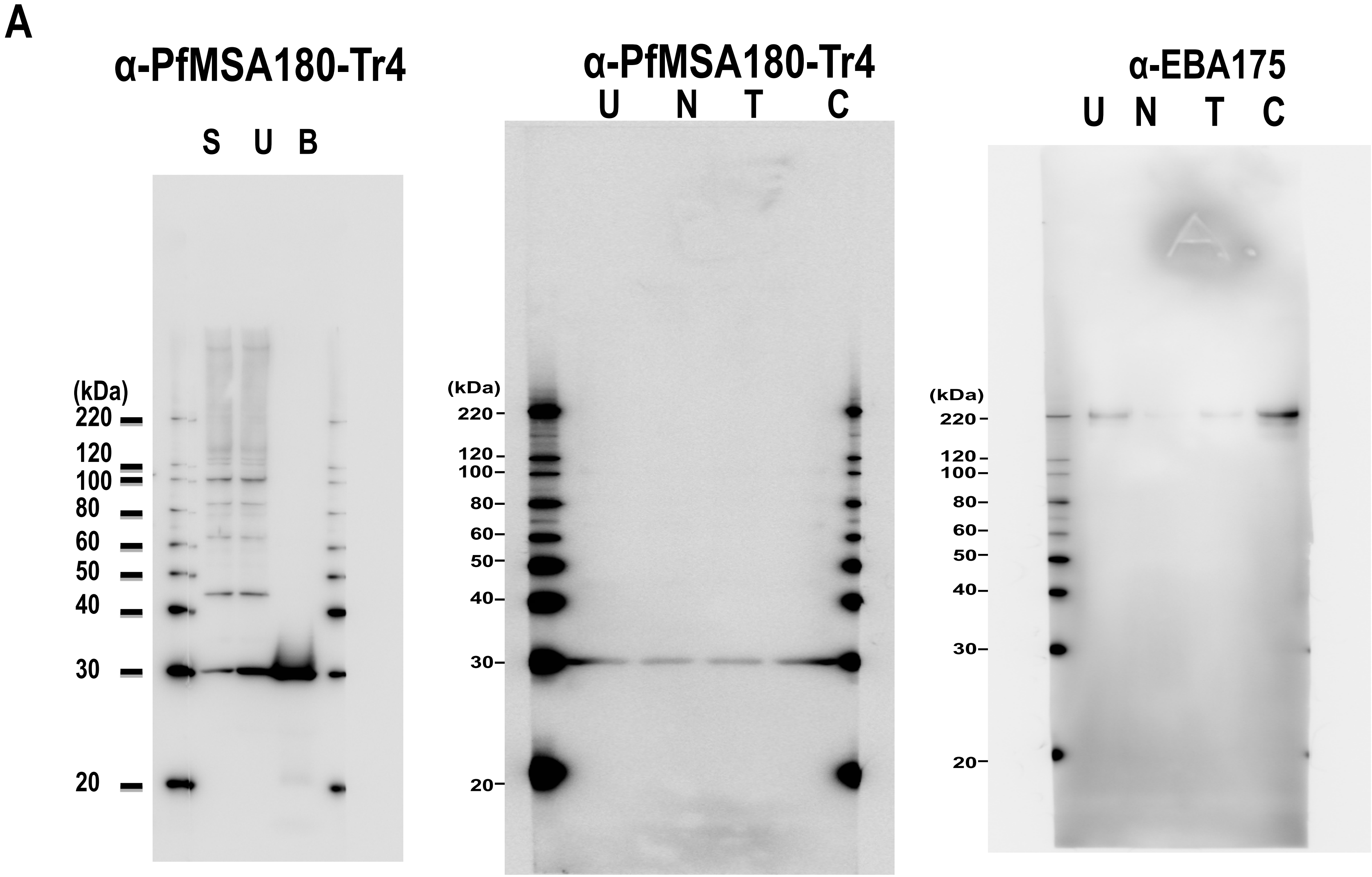
A
α-PfMSA180-Tr4
S U B
(kDa)
220
120
100
80
60
50
40
30
20
α-PfMSA180-Tr4
U N T C
(kDa)
220
120
100
80
60
50
40
30
20
α-EBA175
U N T C
(kDa)
220
120
100
80
60
50
40
30
20
