# Supplementary material for: PfMSA180 is a novel Plasmodium falciparum vaccine antigen that interacts with human erythrocyte integrin associated protein (CD47)
Source: Sci Rep. 2019 Apr 11;9:5923. doi: 10.1038/s41598-019-42366-9 (PMC6459815; doi:10.1038/s41598-019-42366-9)
Supplement: Supplementary file 3 — Supplementary data file 2 [file 41598_2019_42366_MOESM3_ESM.pdf]

SUPPLEMENTARY DATA FILE 2

PfMSA180 is a novel *Plasmodium falciparum* vaccine antigen that interacts with human erythrocyte integrin associated protein (CD47)

Hikaru Nagaoka[1], Chisa Sasaoka[1], Takaaki Yuguchi[1], Bernard N. Kanoi[1], Daisuke Ito[2], Masayuki Morita[1], Rachanee Udomsangpetch[3], Jetsumon Sattabongkot[4], Tomoko Ishino[5], Takafumi Tsuboi[1], Eizo Takashima[1,*]

[1] Division of Malaria Research, Proteo-Science Center, Ehime University, 3 Bunkyo-cho, Matsuyama, Ehime 790-8577, Japan;
[2] Division of Medical Zoology, Department of Microbiology and Immunology, Faculty of Medicine, Tottori University, 86 Nishi-cho, Yonago, Tottori 683-8503, Japan;
[3] Center for Research and Innovation, Faculty of Medical Technology, Mahidol University, Salaya, Nakhosn Pathom 73170, Thailand;
[4] Mahidol Vivax Research Unit, Faculty of Tropical Medicine, Mahidol University, Bangkok 10400, Thailand;
[5] Division of Molecular Parasitology, Proteo-Science Center, Ehime University, Toon Ehime 791-0295, Japan

* Corresponding author
Address: Division of Malaria Research, Proteo-Science Center, Ehime University, Japan, 3 Bunkyo-cho, Matsuyama, Ehime 790-8577, Japan. Tel.: (+81) 89 927 9939. E-mail address: takashima.eizo.mz@ehime-u.ac.jp

+ Majority

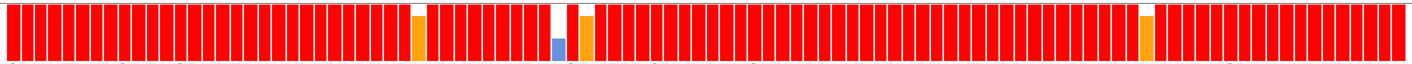

Sequence	Alignment (positions 1–100)	Length
Majority	SAKNFYNISNENGDNTFNNNNNNMDNKKRMYNYNKHKDNNSRYTDNSNKNRDNSNKNRDNYNRNKDKNNTNRDNYNRYKDNNYYYNNSDNNNYNERKRYI	
1.Pf3D7_10_v3\	SAKNFYNISNENGDNTFNNNNNNMDNKKRMYNYNKHKDNDSRYTDNSNKNRDNSNKNRDNYNRNKDKNNTNRDNYNRYKDNNYYYNNSDNNNYNERKRYI	100
1.AA_CAMBONDIA_KH01\	SAKNFYNISNENGDNTFNNNNNNMDNKKRKYNYNKHKDNNSIYTDNSNKNRDNSNKNRDNYNRNKDKNNTNRDNYNRYKDNNYYYNNSDNNNYNERKRYI	100
1.AA_LAOS_Dd2-1\	SAKNFYNISNENGDNTFNNNNNNMDNKKRKYNYNKHKDNNSRYTDNSNKNRDNSNKNRDNYNRNKDKNNTNRDNYNRYKDNKYYYNNSDNNNYNERKRYI	100
1.AA_LAOS_Dd2-2\	SAKNFYNISNENGDNTFNNNNNNMDNKKRKYNYNKHKDNNSRYTDNSNKNRDNSNKNRDNYNRNKDKNNTNRDNYNRYKDNKYYYNNSDNNNYNERKRYI	100
1.AA_THAI_DD2_WTSI\	SAKNFYNISNENGDNTFNNNNNNMDNKKRKYNYNKHKDNNSRYTDNSNKNRDNSNKNRDNYNRNKDKNNTNRDNYNRYKDNKYYYNNSDNNNYNERKRYI	100
1.AA_THAI_T9_94\	SAKNFYNISNENGDNTFNNNNNNMDNKKRKYNYNKHKDNNSRYTDNSNKNRDNSNKNRDNYNRNKDKNNTNRDNYNRYKDNKYYYNNSDNNNYNERKRYI	100
303.1\	SAKNFYNISNENGDNTFNNNNNNMDNKKRMYNYNKHKDNDSRYTDNSNKNRDNSNKNRDNYNRNKDKNNTNRDNYNRYKDNNYYYNNSDNNNYNERKRYI	100
309.1\	SAKNFYNISNENGDNTFNNNNNNMDNKKRMYNYNKHKDNDSRYTDNSNKNRDNSNKNRDNYNRNKDKNNTNRDNYNRYKDNNYYYNNSDNNNYNERKRYI	100
318.1\	SAKNFYNISNENGDNTFNNNNNNMDNKKRMYNYNKHKDNNSRYTDNSNKNRDNSNKNRDNYNRNKDKNNTNRDNYNRYKDNNYYYNNSDNNNYNERKRYI	100
326.1\	SAKNFYNISNENGDNTFNNNNNNMDNKKRMYNYNKHKDNDSRYTDNSNKNRDNSNKNRDNYNRNKDKNNTNRDNYNRYKDNNYYYNNSDNNNYNERKRYI	100
327.1\	SAKNFYNISNENGDNTFNNNNNNMDNKKRMYNYNKHKDNDSRYTDNSNKNRDNSNKNRDNYNRNKDKNNTNRDNYNRYKDNNYYYNNSDNNNYNERKRYI	100
365.1\	SAKNFYNISNENGDNTFNNNNNNMDNKKRMYNYNKHKDNDSRYTDNSNKNRDNSNKNRDNYNRNKDKNNTNRDNYNRYKDNNYYYNNSDNNNYNERKRYI	100
366.1\	SAKNFYNISNENGDNTFNNNNNNMDNKKRMYNYNKHKDNNSRYTDNSNKNRDNSNKNRDNYNRNKDKNNTNRDNYNRYKDNNYYYNNSDNNNYNERKRYI	100
377.1\	SAKNFYNISNENGDNTFNNNNNNMDNKKRMYNYNKHKDNDSRYTDNSNKNRDNSNKNRDNYNRNKDKNNTNRDNYNRYKDNNYYYNNSDNNNYNERKRYI	100
383.1\	SAKNFYNISNENGDNTFNNNNNNMDNKKRMYNYNKHKDNDSRYTDNSNKNRDNSNKNRDNYNRNKDKNNTNRDNYNRYKDNNYYYNNSDNNNYNERKRYI	100
397.1\	SAKNFYNISNENGDNTFNNNNNNMDNKKRMYNYNKHKDNNSRYTDNSNKNRDNSNKNRDNYNRNKDKNNTNRDNYNRYKDNNYYYNNSDNNNYNERKRYI	100
398.1\	SAKNFYNISNENGDNTFNNNNNNMDNKKRMYNYNKHKDNNSRYTDNSNKNRDNSNKNRDNYNRNKDKNNTNRDNYNRYKDNNYYYNNSDNNNYNERKRYI	100
3D7_WTSI\	SAKNFYNISNENGDNTFNNNNNNMDNKKRMYNYNKHKDNDSRYTDNSNKNRDNSNKNRDNYNRNKDKNNTNRDNYNRYKDNNYYYNNSDNNNYNERKRYI	100
58.1\	SAKNFYNISNENGDNTFNNNNNNMDNKKRMYNYNKHKDNNSRYTDNSNKNRDNSNKNRDNYNRNKDKNNTNRDNYNRYKDNNYYYNNSDNNNYNERKRYI	100
707A\	SAKNFYNISNENGDNTFNNNNNNMDNKKRKYNYNKHKDNNSRYTDNSNKNRDNSNKNRDNYNRNKDKNNTNRDNYNRYKDNKYYYNNSDNNNYNERKRYI	100
7G8_2\	SAKNFYNISNENGDNTFNNNNNNMDNKKRKYNYNKHKDNNSRYTDNSNKNRDNSNKNRDNYNRNKDKNNTNRDNYNRYKDNNYYYNNSDNNNYNERKRYI	100
7G8\	SAKNFYNISNENGDNTFNNNNNNMDNKKRKYNYNKHKDNNSRYTDNSNKNRDNSNKNRDNYNRNKDKNNTNRDNYNRYKDNNYYYNNSDNNNYNERKRYI	100
BM_0008\	SAKNFYNISNENGDNTFNNNNNNMDNKKRMYNYNKHKDNNSRYTDNSNKNRDNSNKNRDNYNRNKDKNNTNRDNYNRYKDNNYYYNNSDNNNYNERKRYI	100
BM_0009\	SAKNFYNISNENGDNTFNNNNNNMDNKKRMYNYNKHKDNDSRYTDNSNKNRDNSNKNRDNYNRNKDKNNTNRDNYNRYKDNNYYYNNSDNNNYNERKRYI	100
CS2\	SAKNFYNISNENGDNTFNNNNNNMDNKKRKYNYNKHKDNNSRYTDNSNKNRDNSNKNRDNYNRNKDKNNTNRDNYNRYKDNKYYYNNSDNNNYNERKRYI	100
G224\	SAKNFYNISNENGDNTFNNNNNNMDNKKRKYNYNKHKDNNSRYTDNSNKNRDNSNKNRDNYNRNKDKNNTNRDNYNRYKDNNYYYNNSDNNNYNERKRYI	100
GA01\	SAKNFYNISNENGDNTFNNNNNNMDNKKRMYNYNKHKDNNSRYTDNSNKNRDNSNKNRDNYNRNKDKNNTNRDNYNRYKDNNYYYNNSDNNNYNERKRYI	100
GB4_WTSI\	SAKNFYNISNENGDNTFNNNNNNMDNKKRMYNYNKHKDNNSRYTDNSNKNRDNSNKNRDNYNRNKDKNNTNRDNYNRYKDNNYYYNNSDNNNYNERKRYI	100
GB4\	SAKNFYNISNENGDNTFNNNNNNMDNKKRMYNYNKHKDNNSRYTDNSNKNRDNSNKNRDNYNRNKDKNNTNRDNYNRYKDNNYYYNNSDNNNYNERKRYI	100
GN01\	SAKNFYNISNENGDNTFNNNNNNMDNKKRMYNYNKHKDNNSRYTDNSNKNRDNSNKNRDNYNRNKDKNNTNRDNYNRYKDNNYYYNNSDNNNYNERKRYI	100
H209\	SAKNFYNISNENGDNTFNNNNNNMDNKKRKYNYNKHKDNNSRYTDNSNKNRDNSNKNRDNYNRNKDKNNTNRDNYNRYKDNNYYYNNSDNNNYNERKRYI	100
HB3\	SAKNFYNISNENGDNTFNNNNNNMDNKKRKYNYNKHKDNNSRYTDNSNKNRDNSNKNRDNYNRNKDKNNTNRDNYNRYKDNNYYYNNSDNNNYNERKRYI	100
IT_WTSI\	SAKNFYNISNENGDNTFNNNNNNMDNKKRKYNYNKHKDNNSRYTDNSNKNRDNSNKNRDNYNRNKDKNNTNRDNYNRYKDNKYYYNNSDNNNYNERKRYI	100
KE01\	SAKNFYNISNENGDNTFNNNNNNMDNKKREYNYNKHKDNNSRYTDNSNKNRDNSNKNRDNYNRNKDKNNTNRDNYNRYKDNNYYYNNSDNNNYNERKRYI	100
M219-D\	SAKNFYNISNENGDNTFNNNNNNMDNKKRKYNYNKHKDNNSRYTDNSNKNRDNSNKNRDNYNRNKDKNNTNRDNYNRYKDNNYYYNNSDNNNYNERKRYI	100
M271-D\	SAKNFYNISNENGDNTFNNNNNNMDNKKRKYNYNKHKDNNSRYTDNSNKNRDNSNKNRDNYNRNKDKNNTNRDNYNRYKDNNYYYNNSDNNNYNERKRYI	100
M312-D\	SAKNFYNISNENGDNTFNNNNNNMDNKKRKYNYNKHKDNNSRYTDNSNKNRDNSNKNRDNYNRNKDKNNTNRDNYNRYKDNNYYYNNSDNNNYNERKRYI	100
ML01\	SAKNFYNISNENGDNTFNNNNNNMDNKKRMYNYNKHKDNNSRYTDNSNKNRDNSNKNRDNYNRNKDKNNTNRDNYNRYKDNNYYYNNSDNNNYNERKRYI	100
N011-A\	SAKNFYNISNENGDNTFNNNNNNMDNKKRKYNYNKHKDNNSRYTDNSNKNRDNSNKNRDNYNRNKDKNNTNRDNYNRYKDNNYYYNNSDNNNYNERKRYI	100
N023-A\	SAKNFYNISNENGDNTFNNNNNNMDNKKRKYNYNKHKDNNSRYTDNSNKNRDNSNKNRDNYNRNKDKNNTNRDNYNRYKDNNYYYNNSDNNNYNERKRYI	100
N071-I\	SAKNFYNISNENGDNTFNNNNNNMDNKKRKYNYNKHKDNNSRYTDNSNKNRDNSNKNRDNYNRNKDKNNTNRDNYNRYKDNNYYYNNSDNNNYNERKRYI	100
N164-A\	SAKNFYNISNENGDNTFNNNNNNMDNKKRKYNYNKHKDNNSRYTDNSNKNRDNSNKNRDNYNRNKDKNNTNRDNYNRYKDNNYYYNNSDNNNYNERKRYI	100
N497-C\	SAKNFYNISNENGDNTFNNNNNNMDNKKRKYNYNKHKDNNSRYTDNSNKNRDNSNKNRDNYNRNKDKNNTNRDNYNRYKDNNYYYNNSDNNNYNERKRYI	100
N579-A\	SAKNFYNISNENGDNTFNNNNNNMDNKKRKYNYNKHKDNNSRYTDNSNKNRDNSNKNRDNYNRNKDKNNTNRDNYNRYKDNNYYYNNSDNNNYNERKRYI	100
O079-B\	SAKNFYNISNENGDNTFNNNNNNMDNKKRKYNYNKHKDNNSRYTDNSNKNRDNSNKNRDNYNRNKDKNNTNRDNYNRYKDNNYYYNNSDNNNYNERKRYI	100
O141-A\	SAKNFYNISNENGDNTFNNNNNNMDNKKRKYNYNKHKDNNSRYTDNSNKNRDNSNKNRDNYNRNKDKNNTNRDNYNRYKDNNYYYNNSDNNNYNERKRYI	100
O222-A\	SAKNFYNISNENGDNTFNNNNNNMDNKKRKYNYNKHKDNNSRYTDNSNKNRDNSNKNRDNYNRNKDKNNTNRDNYNRYKDNNYYYNNSDNNNYNERKRYI	100
O306-A\	SAKNFYNISNENGDNTFNNNNNNMDNKKRKYNYNKHKDNNSRYTDNSNKNRDNSNKNRDNYNRNKDKNNTNRDNYNRYKDNNYYYNNSDNNNYNERKRYI	100
O314\	SAKNFYNISNENGDNTFNNNNNNMDNKKRKYNYNKHKDNNSRYTDNSNKNRDNSNKNRDNYNRNKDKNNTNRDNYNRYKDNNYYYNNSDNNNYNERKRYI	100
P164-C\	SAKNFYNISNENGDNTFNNNNNNMDNKKRKYNYNKHKDNNSRYTDNSNKNRDNSNKNRDNYNRNKDKNNTNRDNYNRYKDNNYYYNNSDNNNYNERKRYI	100
P167-B\	SAKNFYNISNENGDNTFNNNNNNMDNKKRKYNYNKHKDNNSRYTDNSNKNRDNSNKNRDNYNRNKDKNNTNRDNYNRYKDNNYYYNNSDNNNYNERKRYI	100
P196J3-C\	SAKNFYNISNENGDNTFNNNNNNMDNKKRKYNYNKHKDNNSRYTDNSNKNRDNSNKNRDNYNRNKDKNNTNRDNYNRYKDNNYYYNNSDNNNYNERKRYI	100
P237-C\	SAKNFYNISNENGDNTFNNNNNNMDNKKRKYNYNKHKDNNSRYTDNSNKNRDNSNKNRDNYNRNKDKNNTNRDNYNRYKDNNYYYNNSDNNNYNERKRYI	100
P241-D\	SAKNFYNISNENGDNTFNNNNNNMDNKKRKYNYNKHKDNNSRYTDNSNKNRDNSNKNRDNYNRNKDKNNTNRDNYNRYKDNNYYYNNSDNNNYNERKRYI	100
PS097\	SAKNFYNISNENGDNTFNNNNNNMDNKKRMYNYNKHKDNDSRYTDNSNKNRDNSNKNRDNYNRNKDKNNTNRDNYNRYKDNNYYYNNSDNNNYNERKRYI	100
PS103\	SAKNFYNISNENGDNTFNNNNNNMDNKKRMYNYNKHKDNNSRYTDNSNKNRDNSNKNRDNYNRNKDKNNTNRDNYNRYKDNNYYYNNSDNNNYNERKRYI	100
PS122_G11\	SAKNFYNISNENGDNTFNNNNNNMDNKKRMYNYNKHKDNNSRYTDNSNKNRDNSNKNRDNYNRNKDKNNTNRDNYNRYKDNNYYYNNSDNNNYNERKRYI	100
PS149\	SAKNFYNISNENGDNTFNNNNNNMDNKKRMYNYNKHKDNNSRYTDNSNKNRDNSNKNRDNYNRNKDKNNTNRDNYNRYKDNNYYYNNSDNNNYNERKRYI	100
PS170\	SAKNFYNISNENGDNTFNNNNNNMDNKKRMYNYNKHKDNDSRYTDNSNKNRDNSNKNRDNYNRNKDKNNTNRDNYNRYKDNNYYYNNSDNNNYNERKRYI	100
PS183\	SAKNFYNISNENGDNTFNNNNNNMDNKKRMYNYNKHKDNNSRYTDNSNKNRDNSNKNRDNYNRNKDKNNTNRDNYNRYKDNNYYYNNSDNNNYNERKRYI	100
PS186\	SAKNFYNISNENGDNTFNNNNNNMDNKKRMYNYNKHKDNNSRYTDNSNKNRDNSNKNRDNYNRNKDKNNTNRDNYNRYKDNNYYYNNSDNNNYNERKRYI	100
PS189\	SAKNFYNISNENGDNTFNNNNNNMDNKKRMYNYNKHKDNNSRYTDNSNKNRDNSNKNRDNYNRNKDKNNTNRDNYNRYKDNNYYYNNSDNNNYNERKRYI	100
PS206_E11\	SAKNFYNISNENGDNTFNNNNNNMDNKKRMYNYNKHKDNNSRYTDNSNKNRDNSNKNRDNYNRNKDKNNTNRDNYNRYKDNNYYYNNSDNNNYNERKRYI	100
PS250\	SAKNFYNISNENGDNTFNNNNNNMDNKKRMYNYNKHKDNNSRYTDNSNKNRDNSNKNRDNYNRNKDKNNTNRDNYNRYKDNNYYYNNSDNNNYNERKRYI	100
RV_3600\	SAKNFYNISNENGDNTFNNNNNNMDNKKRMYNYNKHKDNDSRYTDNSNKNRDNSNKNRDNYNRNKDKNNTNRDNYNRYKDNNYYYNNSDNNNYNERKRYI	100
RV_3606\	SAKNFYNISNENGDNTFNNNNNNMDNKKRMYNYNKHKDNDSRYTDNSNKNRDNSNKNRDNYNRNKDKNNTNRDNYNRYKDNNYYYNNSDNNNYNERKRYI	100
RV_3610\	SAKNFYNISNENGDNTFNNNNNNMDNKKRMYNYNKHKDNDSRYTDNSNKNRDNSNKNRDNYNRNKDKNNTNRDNYNRYKDNNYYYNNSDNNNYNERKRYI	100
RV_3611\	SAKNFYNISNENGDNTFNNNNNNMDNKKRMYNYNKHKDNDSRYTDNSNKNRDNSNKNRDNYNRNKDKNNTNRDNYNRYKDNNYYYNNSDNNNYNERKRYI	100
RV_3614\	SAKNFYNISNENGDNTFNNNNNNMDNKKRMYNYNKHKDNDSRYTDNSNKNRDNSNKNRDNYNRNKDKNNTNRDNYNRYKDNNYYYNNSDNNNYNERKRYI	100
RV_3630\	SAKNFYNISNENGDNTFNNNNNNMDNKKRMYNYNKHKDNNSRYTDNSNKNRDNSNKNRDNYNRNKDKNNTNRDNYNRYKDNNYYYNNSDNNNYNERKRYI	100

Asian isolates

RV_3635\	SAKNFYNISNENGDNTFNNNNNNMDNKKRMYNYNKHKDNDSRYTDNSNKNRDNSNKNRDNYNRNKDKNNTNRDNYNRYKDNNYYYNNSDNNNYNERKRYI	100
RV_3637\	SAKNFYNISNENGDNTFNNNNNNMDNKKRMYNYNKHKDNDSRYTDNSNKNRDNSNKNRDNYNRNKDKNNTNRDNYNRYKDNNYYYNNSDNNNYNERKRYI	100
RV_3642\	SAKNFYNISNENGDNTFNNNNNNMDNKKRMYNYNKHKDNNSRYTDNSNKNRDNSNKNRDNYNRNKDKNNTNRDNYNRYKDNNYYYNNSDNNNYNERKRYI	100
RV_3650\	SAKNFYNISNENGDNTFNNNNNNMDNKKRMYNYNKHKDNDSRYTDNSNKNRDNSNKNRDNYNRNKDKNNTNRDNYNRYKDNNYYYNNSDNNNYNERKRYI	100
RV_3655\	SAKNFYNISNENGDNTFNNNNNNMDNKKRMYNYNKHKDNDSRYTDNSNKNRDNSNKNRDNYNRNKDKNNTNRDNYNRYKDNNYYYNNSDNNNYNERKRYI	100
RV_3671\	SAKNFYNISNENGDNTFNNNNNNMDNKKRMYNYNKHKDNNSRYTDNSNKNRDNSNKNRDNYNRNKDKNNTNRDNYNRYKDNNYYYNNSDNNNYNERKRYI	100
RV_3672\	SAKNFYNISNENGDNTFNNNNNNMDNKKRMYNYNKHKDNDSRYTDNSNKNRDNSNKNRDNYNRNKDKNNTNRDNYNRYKDNNYYYNNSDNNNYNERKRYI	100
RV_3673\	SAKNFYNISNENGDNTFNNNNNNMDNKKRMYNYNKHKDNDSRYTDNSNKNRDNSNKNRDNYNRNKDKNNTNRDNYNRYKDNNYYYNNSDNNNYNERKRYI	100
RV_3675\	SAKNFYNISNENGDNTFNNNNNNMDNKKRMYNYNKHKDNDSRYTDNSNKNRDNSNKNRDNYNRNKDKNNTNRDNYNRYKDNNYYYNNSDNNNYNERKRYI	100
RV_3687\	SAKNFYNISNENGDNTFNNNNNNMDNKKRMYNYNKHKDNNSRYTDNSNKNRDNSNKNRDNYNRNKDKNNTNRDNYNRYKDNNYYYNNSDNNNYNERKRYI	100
RV_3695\	SAKNFYNISNENGDNTFNNNNNNMDNKKRMYNYNKHKDNNSRYTDNSNKNRDNSNKNRDNYNRNKDKNNTNRDNYNRYKDNNYYYNNSDNNNYNERKRYI	100
RV_3696\	SAKNFYNISNENGDNTFNNNNNNMDNKKRMYNYNKHKDNDSRYTDNSNKNRDNSNKNRDNYNRNKDKNNTNRDNYNRYKDNNYYYNNSDNNNYNERKRYI	100
RV_3701\	SAKNFYNISNENGDNTFNNNNNNMDNKKRMYNYNKHKDNDSRYTDNSNKNRDNSNKNRDNYNRNKDKNNTNRDNYNRYKDNNYYYNNSDNNNYNERKRYI	100
RV_3702\	SAKNFYNISNENGDNTFNNNNNNMDNKKRMYNYNKHKDNDSRYTDNSNKNRDNSNKNRDNYNRNKDKNNTNRDNYNRYKDNNYYYNNSDNNNYNERKRYI	100
RV_3703\	SAKNFYNISNENGDNTFNNNNNNMDNKKRMYNYNKHKDNDSRYTDNSNKNRDNSNKNRDNYNRNKDKNNTNRDNYNRYKDNNYYYNNSDNNNYNERKRYI	100
RV_3708\	SAKNFYNISNENGDNTFNNNNNNMDNKKRMYNYNKHKDNNSRYTDNSNKNRDNSNKNRDNYNRNKDKNNTNRDNYNRYKDNNYYYNNSDNNNYNERKRYI	100
RV_3714\	SAKNFYNISNENGDNTFNNNNNNMDNKKRMYNYNKHKDNNSRYTDNSNKNRDNSNKNRDNYNRNKDKNNTNRDNYNRYKDNNYYYNNSDNNNYNERKRYI	100
RV_3717\	SAKNFYNISNENGDNTFNNNNNNMDNKKRMYNYNKHKDNDSRYTDNSNKNRDNSNKNRDNYNRNKDKNNTNRDNYNRYKDNNYYYNNSDNNNYNERKRYI	100
RV_3729\	SAKNFYNISNENGDNTFNNNNNNMDNKKRMYNYNKHKDNNSRYTDNSNKNRDNSNKNRDNYNRNKDKNNTNRDNYNRYKDNNYYYNNSDNNNYNERKRYI	100
RV_3730\	SAKNFYNISNENGDNTFNNNNNNMDNKKRMYNYNKHKDNNSRYTDNSNKNRDNSNKNRDNYNRNKDKNNTNRDNYNRYKDNNYYYNNSDNNNYNERKRYI	100
RV_3731\	SAKNFYNISNENGDNTFNNNNNNMDNKKRMYNYNKHKDNDSRYTDNSNKNRDNSNKNRDNYNRNKDKNNTNRDNYNRYKDNNYYYNNSDNNNYNERKRYI	100
RV_3735\	SAKNFYNISNENGDNTFNNNNNNMDNKKRMYNYNKHKDNNSRYTDNSNKNRDNSNKNRDNYNRNKDKNNTNRDNYNRYKDNNYYYNNSDNNNYNERKRYI	100
RV_3736\	SAKNFYNISNENGDNTFNNNNNNMDNKKRMYNYNKHKDNNSRYTDNSNKNRDNSNKNRDNYNRNKDKNNTNRDNYNRYKDNNYYYNNSDNNNYNERKRYI	100
RV_3737\	SAKNFYNISNENGDNTFNNNNNNMDNKKRMYNYNKHKDNDSRYTDNSNKNRDNSNKNRDNYNRNKDKNNTNRDNYNRYKDNNYYYNNSDNNNYNERKRYI	100
RV_3739\	SAKNFYNISNENGDNTFNNNNNNMDNKKRMYNYNKHKDNDSRYTDNSNKNRDNSNKNRDNYNRNKDKNNTNRDNYNRYKDNNYYYNNSDNNNYNERKRYI	100
RV_3740\	SAKNFYNISNENGDNTFNNNNNNMDNKKRMYNYNKHKDNDSRYTDNSNKNRDNSNKNRDNYNRNKDKNNTNRDNYNRYKDNNYYYNNSDNNNYNERKRYI	100
RV_3741\	SAKNFYNISNENGDNTFNNNNNNMDNKKRMYNYNKHKDNDSRYTDNSNKNRDNSNKNRDNYNRNKDKNNTNRDNYNRYKDNNYYYNNSDNNNYNERKRYI	100
RV_3764\	SAKNFYNISNENGDNTFNNNNNNMDNKKRMYNYNKHKDNDSRYTDNSNKNRDNSNKNRDNYNRNKDKNNTNRDNYNRYKDNNYYYNNSDNNNYNERKRYI	100
RV_3766\	SAKNFYNISNENGDNTFNNNNNNMDNKKRMYNYNKHKDNDSRYTDNSNKNRDNSNKNRDNYNRNKDKNNTNRDNYNRYKDNNYYYNNSDNNNYNERKRYI	100
RV_3769\	SAKNFYNISNENGDNTFNNNNNNMDNKKRMYNYNKHKDNNSRYTDNSNKNRDNSNKNRDNYNRNKDKNNTNRDNYNRYKDNNYYYNNSDNNNYNERKRYI	100
SantaLucia_Salvadorl\	SAKNFYNISNENGDNTFNNNNNNMDNKKRMYNYNKHKDNDSRYTDNSNKNRDNSNKNRDNYNRNKDKNNTNRDNYNRYKDNNYYYNNSDNNNYNERKRYI	100
SD01\	SAKNFYNISNENGDNTFNNNNNNMDNKKRKYNYNKHKDNDSRYTDNSNKNRDNSNKNRDNYNRNKDKNNTNRDNYNRYKDNNYYYNNSDNNNYNERKRYI	100
SenT001.08\	SAKNFYNISNENGDNTFNNNNNNMDNKKRMYNYNKHKDNDSRYTDNSNKNRDNSNKNRDNYNRNKDKNNTNRDNYNRYKDNNYYYNNSDNNNYNERKRYI	100
SenT002.07\	SAKNFYNISNENGDNTFNNNNNNMDNKKRMYNYNKHKDNDSRYTDNSNKNRDNSNKNRDNYNRNKDKNNTNRDNYNRYKDNNYYYNNSDNNNYNERKRYI	100
SenT002.09\	SAKNFYNISNENGDNTFNNNNNNMDNKKRMYNYNKHKDNNSRYTDNSNKNRDNSNKNRDNYNRNKDKNNTNRDNYNRYKDNNYYYNNSDNNNYNERKRYI	100
SenT015.09\	SAKNFYNISNENGDNTFNNNNNNMDNKKRMYNYNKHKDNDSRYTDNSNKNRDNSNKNRDNYNRNKDKNNTNRDNYNRYKDNNYYYNNSDNNNYNERKRYI	100
SenT018.09\	SAKNFYNISNENGDNTFNNNNNNMDNKKRMYNYNKHKDNDSRYTDNSNKNRDNSNKNRDNYNRNKDKNNTNRDNYNRYKDNNYYYNNSDNNNYNERKRYI	100
SenT021.09\	SAKNFYNISNENGDNTFNNNNNNMDNKKRMYNYNKHKDNDSRYTDNSNKNRDNSNKNRDNYNRNKDKNNTNRDNYNRYKDNNYYYNNSDNNNYNERKRYI	100
SenT022.09\	SAKNFYNISNENGDNTFNNNNNNMDNKKRMYNYNKHKDNNSRYTDNSNKNRDNSNKNRDNYNRNKDKNNTNRDNYNRYKDNNYYYNNSDNNNYNERKRYI	100
SenT024.08\	SAKNFYNISNENGDNTFNNNNNNMDNKKRMYNYNKHKDNDSRYTDNSNKNRDNSNKNRDNYNRNKDKNNTNRDNYNRYKDNNYYYNNSDNNNYNERKRYI	100
SenT029.09\	SAKNFYNISNENGDNTFNNNNNNMDNKKRMYNYNKHKDNDSRYTDNSNKNRDNSNKNRDNYNRNKDKNNTNRDNYNRYKDNNYYYNNSDNNNYNERKRYI	100
SenT032.09\	SAKNFYNISNENGDNTFNNNNNNMDNKKRMYNYNKHKDNNSRYTDNSNKNRDNSNKNRDNYNRNKDKNNTNRDNYNRYKDNNYYYNNSDNNNYNERKRYI	100
SenT033.08\	SAKNFYNISNENGDNTFNNNNNNMDNKKRMYNYNKHKDNNSRYTDNSNKNRDNSNKNRDNYNRNKDKNNTNRDNYNRYKDNNYYYNNSDNNNYNERKRYI	100
SenT033.09\	SAKNFYNISNENGDNTFNNNNNNMDNKKRKYNYNKHKDNNSRYTDNSNKNRDNSNKNRDNYNRNKDKNNTNRDNYNRYKDNNYYYNNSDNNNYNERKRYI	100
SenT042.09\	SAKNFYNISNENGDNTFNNNNNNMDNKKRMYNYNKHKDNNSRYTDNSNKNRDNSNKNRDNYNRNKDKNNTNRDNYNRYKDNNYYYNNSDNNNYNERKRYI	100
SenT044.10\	SAKNFYNISNENGDNTFNNNNNNMDNKKRMYNYNKHKDNNSRYTDNSNKNRDNSNKNRDNYNRNKDKNNTNRDNYNRYKDNNYYYNNSDNNNYNERKRYI	100
SenT046.10\	SAKNFYNISNENGDNTFNNNNNNMDNKKRMYNYNKHKDNNSRYTDNSNKNRDNSNKNRDNYNRNKDKNNTNRDNYNRYKDNNYYYNNSDNNNYNERKRYI	100
SenT047.09\	SAKNFYNISNENGDNTFNNNNNNMDNKKRMYNYNKHKDNDSRYTDNSNKNRDNSNKNRDNYNRNKDKNNTNRDNYNRYKDNNYYYNNSDNNNYNERKRYI	100
SenT061.09\	SAKNFYNISNENGDNTFNNNNNNMDNKKRMYNYNKHKDNDSRYTDNSNKNRDNSNKNRDNYNRNKDKNNTNRDNYNRYKDNNYYYNNSDNNNYNERKRYI	100
SenT063.07\	SAKNFYNISNENGDNTFNNNNNNMDNKKRMYNYNKHKDNNSRYTDNSNKNRDNSNKNRDNYNRNKDKNNTNRDNYNRYKDNNYYYNNSDNNNYNERKRYI	100
SenT066.08\	SAKNFYNISNENGDNTFNNNNNNMDNKKRMYNYNKHKDNDSRYTDNSNKNRDNSNKNRDNYNRNKDKNNTNRDNYNRYKDNNYYYNNSDNNNYNERKRYI	100
SenT067.09\	SAKNFYNISNENGDNTFNNNNNNMDNKKRMYNYNKHKDNNSRYTDNSNKNRDNSNKNRDNYNRNKDKNNTNRDNYNRYKDNNYYYNNSDNNNYNERKRYI	100
SenT075.10\	SAKNFYNISNENGDNTFNNNNNNMDNKKRMYNYNKHKDNNSRYTDNSNKNRDNSNKNRDNYNRNKDKNNTNRDNYNRYKDNNYYYNNSDNNNYNERKRYI	100
SenT077.08\	SAKNFYNISNENGDNTFNNNNNNMDNKKRMYNYNKHKDNDSRYTDNSNKNRDNSNKNRDNYNRNKDKNNTNRDNYNRYKDNNYYYNNSDNNNYNERKRYI	100
SenT087.08\	SAKNFYNISNENGDNTFNNNNNNMDNKKRMYNYNKHKDNDSRYTDNSNKNRDNSNKNRDNYNRNKDKNNTNRDNYNRYKDNNYYYNNSDNNNYNERKRYI	100
SenT090.08\	SAKNFYNISNENGDNTFNNNNNNMDNKKRMYNYNKHKDNDSRYTDNSNKNRDNSNKNRDNYNRNKDKNNTNRDNYNRYKDNNYYYNNSDNNNYNERKRYI	100
SenT090.09\	SAKNFYNISNENGDNTFNNNNNNMDNKKRMYNYNKHKDNNSRYTDNSNKNRDNSNKNRDNYNRNKDKNNTNRDNYNRYKDNNYYYNNSDNNNYNERKRYI	100
SenT093.09\	SAKNFYNISNENGDNTFNNNNNNMDNKKRMYNYNKHKDNNSRYTDNSNKNRDNSNKNRDNYNRNKDKNNTNRDNYNRYKDNNYYYNNSDNNNYNERKRYI	100
SenT094.09\	SAKNFYNISNENGDNTFNNNNNNMDNKKRMYNYNKHKDNNSRYTDNSNKNRDNSNKNRDNYNRNKDKNNTNRDNYNRYKDNNYYYNNSDNNNYNERKRYI	100
SenT097.09\	SAKNFYNISNENGDNTFNNNNNNMDNKKRMYNYNKHKDNDSRYTDNSNKNRDNSNKNRDNYNRNKDKNNTNRDNYNRYKDNNYYYNNSDNNNYNERKRYI	100
SenT101.09\	SAKNFYNISNENGDNTFNNNNNNMDNKKRMYNYNKHKDNNSRYTDNSNKNRDNSNKNRDNYNRNKDKNNTNRDNYNRYKDNNYYYNNSDNNNYNERKRYI	100
SenT102.08\	SAKNFYNISNENGDNTFNNNNNNMDNKKRMYNYNKHKDNNSRYTDNSNKNRDNSNKNRDNYNRNKDKNNTNRDNYNRYKDNNYYYNNSDNNNYNERKRYI	100
SenT106.08\	SAKNFYNISNENGDNTFNNNNNNMDNKKRMYNYNKHKDNDSRYTDNSNKNRDNSNKNRDNYNRNKDKNNTNRDNYNRYKDNNYYYNNSDNNNYNERKRYI	100
SenT113.09\	SAKNFYNISNENGDNTFNNNNNNMDNKKRMYNYNKHKDNNSRYTDNSNKNRDNSNKNRDNYNRNKDKNNTNRDNYNRYKDNNYYYNNSDNNNYNERKRYI	100
SenT119.09\	SAKNFYNISNENGDNTFNNNNNNMDNKKRMYNYNKHKDNNSRYTDNSNKNRDNSNKNRDNYNRNKDKNNTNRDNYNRYKDNNYYYNNSDNNNYNERKRYI	100
SenT123.09\	SAKNFYNISNENGDNTFNNNNNNMDNKKRMYNYNKHKDNDSRYTDNSNKNRDNSNKNRDNYNRNKDKNNTNRDNYNRYKDNNYYYNNSDNNNYNERKRYI	100
SenT127.09\	SAKNFYNISNENGDNTFNNNNNNMDNKKRMYNYNKHKDNNSRYTDNSNKNRDNSNKNRDNYNRNKDKNNTNRDNYNRYKDNNYYYNNSDNNNYNERKRYI	100
SenT128.08\	SAKNFYNISNENGDNTFNNNNNNMDNKKRMYNYNKHKDNNSRYTDNSNKNRDNSNKNRDNYNRNKDKNNTNRDNYNRYKDNNYYYNNSDNNNYNERKRYI	100
SenT135.09\	SAKNFYNISNENGDNTFNNNNNNMDNKKRMYNYNKHKDNDSRYTDNSNKNRDNSNKNRDNYNRNKDKNNTNRDNYNRYKDNNYYYNNSDNNNYNERKRYI	100
SenT137.08\	SAKNFYNISNENGDNTFNNNNNNMDNKKRMYNYNKHKDNDSRYTDNSNKNRDNSNKNRDNYNRNKDKNNTNRDNYNRYKDNNYYYNNSDNNNYNERKRYI	100
SenT137.09\	SAKNFYNISNENGDNTFNNNNNNMDNKKRMYNYNKHKDNDSRYTDNSNKNRDNSNKNRDNYNRNKDKNNTNRDNYNRYKDNNYYYNNSDNNNYNERKRYI	100
SenT139.08\	SAKNFYNISNENGDNTFNNNNNNMDNKKRMYNYNKHKDNDSRYTDNSNKNRDNSNKNRDNYNRNKDKNNTNRDNYNRYKDNNYYYNNSDNNNYNERKRYI	100
SenT140.08\	SAKNFYNISNENGDNTFNNNNNNMDNKKRMYNYNKHKDNNSRYTDNSNKNRDNSNKNRDNYNRNKDKNNTNRDNYNRYKDNNYYYNNSDNNNYNERKRYI	100
SenT142.09\	SAKNFYNISNENGDNTFNNNNNNMDNKKRMYNYNKHKDNNSRYTDNSNKNRDNSNKNRDNYNRNKDKNNTNRDNYNRYKDNNYYYNNSDNNNYNERKRYI	100
SenT144.08\	SAKNFYNISNENGDNTFNNNNNNMDNKKRMYNYNKHKDNNSRYTDNSNKNRDNSNKNRDNYNRNKDKNNTNRDNYNRYKDNNYYYNNSDNNNYNERKRYI	100
SenT145.08\	SAKNFYNISNENGDNTFNNNNNNMDNKKRMYNYNKHKDNDSRYTDNSNKNRDNSNKNRDNYNRNKDKNNTNRDNYNRYKDNNYYYNNSDNNNYNERKRYI	100

SenT149.09\	SAKNFYNISNENGDNTFNNNNNNMDNKKRMYNYNKHKDNDSRYTDNSNKNRDNSNKNRDNYNRNKDKNNTNRDNYNRYKDNNYYYNNSDNNNYNERKRYI	100
SenT150.09\	SAKNFYNISNENGDNTFNNNNNNMDNKKRMYNYNKHKDNDSRYTDNSNKNRDNSNKNRDNYNRNKDKNNTNRDNYNRYKDNNYYYNNSDNNNYNERKRYI	100
SenT151.09\	SAKNFYNISNENGDNTFNNNNNNMDNKKRMYNYNKHKDNDSRYTDNSNKNRDNSNKNRDNYNRNKDKNNTNRDNYNRYKDNNYYYNNSDNNNYNERKRYI	100
SenT170.08\	SAKNFYNISNENGDNTFNNNNNNMDNKKRMYNYNKHKDNNSRYTDNSNKNRDNSNKNRDNYNRNKDKNNTNRDNYNRYKDNNYYYNNSDNNNYNERKRYI	100
SenT175.08\	SAKNFYNISNENGDNTFNNNNNNMDNKKRMYNYNKHKDNNSRYTDNSNKNRDNSNKNRDNYNRNKDKNNTNRDNYNRYKDNNYYYNNSDNNNYNERKRYI	100
SenT179.08\	SAKNFYNISNENGDNTFNNNNNNMDNKKRMYNYNKHKDNNSRYTDNSNKNRDNSNKNRDNYNRNKDKNNTNRDNYNRYKDNNYYYNNSDNNNYNERKRYI	100
SenT180.08\	SAKNFYNISNENGDNTFNNNNNNMDNKKRMYNYNKHKDNNSRYTDNSNKNRDNSNKNRDNYNRNKDKNNTNRDNYNRYKDNNYYYNNSDNNNYNERKRYI	100
SenT190.08\	SAKNFYNISNENGDNTFNNNNNNMDNKKRMYNYNKHKDNNSRYTDNSNKNRDNSNKNRDNYNRNKDKNNTNRDNYNRYKDNNYYYNNSDNNNYNERKRYI	100
SenT197.08\	SAKNFYNISNENGDNTFNNNNNNMDNKKRMYNYNKHKDNNSRYTDNSNKNRDNSNKNRDNYNRNKDKNNTNRDNYNRYKDNNYYYNNSDNNNYNERKRYI	100
SenT224.08\	SAKNFYNISNENGDNTFNNNNNNMDNKKRMYNYNKHKDNNSRYTDNSNKNRDNSNKNRDNYNRNKDKNNTNRDNYNRYKDNNYYYNNSDNNNYNERKRYI	100
SenT227.08\	SAKNFYNISNENGDNTFNNNNNNMDNKKRMYNYNKHKDNDSRYTDNSNKNRDNSNKNRDNYNRNKDKNNTNRDNYNRYKDNNYYYNNSDNNNYNERKRYI	100
SenT230.08\	SAKNFYNISNENGDNTFNNNNNNMDNKKRMYNYNKHKDNDSRYTDNSNKNRDNSNKNRDNYNRNKDKNNTNRDNYNRYKDNNYYYNNSDNNNYNERKRYI	100
SenT235.08\	SAKNFYNISNENGDNTFNNNNNNMDNKKRMYNYNKHKDNNSRYTDNSNKNRDNSNKNRDNYNRNKDKNNTNRDNYNRYKDNNYYYNNSDNNNYNERKRYI	100
SenT238.08\	SAKNFYNISNENGDNTFNNNNNNMDNKKRMYNYNKHKDNDSRYTDNSNKNRDNSNKNRDNYNRNKDKNNTNRDNYNRYKDNNYYYNNSDNNNYNERKRYI	100
SEV2_H5\	SAKNFYNISNENGDNTFNNNNNNMDNKKRKYNYNKHKDNDSRYTDNSNKNRDNSNKNRDNYNRNKDKNNTNRDNYNRYKDNNYYYNNSDNNNYNERKRYI	100
SN01\	SAKNFYNISNENGDNTFNNNNNNMDNKKRMYNYNKHKDNDSRYTDNSNKNRDNSNKNRDNYNRNKDKNNTNRDNYNRYKDNNYYYNNSDNNNYNERKRYI	100
TG01\	SAKNFYNISNENGDNTFNNNNNNMDNKKRMYNYNKHKDNNSRYTDNSNKNRDNSNKNRDNYNRNKDKNNTNRDNYNRYKDNNYYYNNSDNNNYNERKRYI	100
TRIPS_301\	SAKNFYNISNENGDNTFNNNNNNMDNKKRMYNYNKHKDNDSRYTDNSNKNRDNSNKNRDNYNRNKDKNNTNRDNYNRYKDNNYYYNNSDNNNYNERKRYI	100
TRIPS_303\	SAKNFYNISNENGDNTFNNNNNNMDNKKRMYNYNKHKDNDSRYTDNSNKNRDNSNKNRDNYNRNKDKNNTNRDNYNRYKDNNYYYNNSDNNNYNERKRYI	100
TRIPS_331\	SAKNFYNISNENGDNTFNNNNNNMDNKKRMYNYNKHKDNDSRYTDNSNKNRDNSNKNRDNYNRNKDKNNTNRDNYNRYKDNNYYYNNSDNNNYNERKRYI	100
TRIPS_355\	SAKNFYNISNENGDNTFNNNNNNMDNKKRMYNYNKHKDNDSRYTDNSNKNRDNSNKNRDNYNRNKDKNNTNRDNYNRYKDNNYYYNNSDNNNYNERKRYI	100
TRIPS_364\	SAKNFYNISNENGDNTFNNNNNNMDNKKRMYNYNKHKDNDSRYTDNSNKNRDNSNKNRDNYNRNKDKNNTNRDNYNRYKDNNYYYNNSDNNNYNERKRYI	100
TRIPS_373\	SAKNFYNISNENGDNTFNNNNNNMDNKKRMYNYNKHKDNNSRYTDNSNKNRDNSNKNRDNYNRNKDKNNTNRDNYNRYKDNNYYYNNSDNNNYNERKRYI	100
TRIPS_410\	SAKNFYNISNENGDNTFNNNNNNMDNKKRMYNYNKHKDNNSRYTDNSNKNRDNSNKNRDNYNRNKDKNNTNRDNYNRYKDNNYYYNNSDNNNYNERKRYI	100
TRIPS_433\	SAKNFYNISNENGDNTFNNNNNNMDNKKRMYNYNKHKDNDSRYTDNSNKNRDNSNKNRDNYNRNKDKNNTNRDNYNRYKDNNYYYNNSDNNNYNERKRYI	100
TRIPS_437\	SAKNFYNISNENGDNTFNNNNNNMDNKKRMYNYNKHKDNNSRYTDNSNKNRDNSNKNRDNYNRNKDKNNTNRDNYNRYKDNNYYYNNSDNNNYNERKRYI	100
TRIPS_440\	SAKNFYNISNENGDNTFNNNNNNMDNKKRMYNYNKHKDNDSRYTDNSNKNRDNSNKNRDNYNRNKDKNNTNRDNYNRYKDNNYYYNNSDNNNYNERKRYI	100
TRIPS_456\	SAKNFYNISNENGDNTFNNNNNNMDNKKRMYNYNKHKDNNSRYTDNSNKNRDNSNKNRDNYNRNKDKNNTNRDNYNRYKDNNYYYNNSDNNNYNERKRYI	100
TRIPS_461\	SAKNFYNISNENGDNTFNNNNNNMDNKKRMYNYNKHKDNDSRYTDNSNKNRDNSNKNRDNYNRNKDKNNTNRDNYNRYKDNNYYYNNSDNNNYNERKRYI	100
TRIPS_467\	SAKNFYNISNENGDNTFNNNNNNMDNKKRMYNYNKHKDNNSRYTDNSNKNRDNSNKNRDNYNRNKDKNNTNRDNYNRYKDNNYYYNNSDNNNYNERKRYI	100
TRIPS_470\	SAKNFYNISNENGDNTFNNNNNNMDNKKRMYNYNKHKDNNSRYTDNSNKNRDNSNKNRDNYNRNKDKNNTNRDNYNRYKDNNYYYNNSDNNNYNERKRYI	100
TRIPS_474\	SAKNFYNISNENGDNTFNNNNNNMDNKKRMYNYNKHKDNNSRYTDNSNKNRDNSNKNRDNYNRNKDKNNTNRDNYNRYKDNNYYYNNSDNNNYNERKRYI	100
TRIPS_480\	SAKNFYNISNENGDNTFNNNNNNMDNKKRMYNYNKHKDNDSRYTDNSNKNRDNSNKNRDNYNRNKDKNNTNRDNYNRYKDNNYYYNNSDNNNYNERKRYI	100
TRIPS_482\	SAKNFYNISNENGDNTFNNNNNNMDNKKRMYNYNKHKDNNSRYTDNSNKNRDNSNKNRDNYNRNKDKNNTNRDNYNRYKDNNYYYNNSDNNNYNERKRYI	100
TRIPS_487\	SAKNFYNISNENGDNTFNNNNNNMDNKKRMYNYNKHKDNNSRYTDNSNKNRDNSNKNRDNYNRNKDKNNTNRDNYNRYKDNNYYYNNSDNNNYNERKRYI	100
TRIPS_490\	SAKNFYNISNENGDNTFNNNNNNMDNKKRMYNYNKHKDNNSRYTDNSNKNRDNSNKNRDNYNRNKDKNNTNRDNYNRYKDNNYYYNNSDNNNYNERKRYI	100
TRIPS_499\	SAKNFYNISNENGDNTFNNNNNNMDNKKRMYNYNKHKDNNSRYTDNSNKNRDNSNKNRDNYNRNKDKNNTNRDNYNRYKDNNYYYNNSDNNNYNERKRYI	100
TRIPS_501\	SAKNFYNISNENGDNTFNNNNNNMDNKKRMYNYNKHKDNDSRYTDNSNKNRDNSNKNRDNYNRNKDKNNTNRDNYNRYKDNNYYYNNSDNNNYNERKRYI	100
TRIPS_504\	SAKNFYNISNENGDNTFNNNNNNMDNKKRMYNYNKHKDNNSRYTDNSNKNRDNSNKNRDNYNRNKDKNNTNRDNYNRYKDNNYYYNNSDNNNYNERKRYI	100
TRIPS_700\	SAKNFYNISNENGDNTFNNNNNNMDNKKRMYNYNKHKDNDSRYTDNSNKNRDNSNKNRDNYNRNKDKNNTNRDNYNRYKDNNYYYNNSDNNNYNERKRYI	100
TRIPS_704\	SAKNFYNISNENGDNTFNNNNNNMDNKKRMYNYNKHKDNNSRYTDNSNKNRDNSNKNRDNYNRNKDKNNTNRDNYNRYKDNNYYYNNSDNNNYNERKRYI	100
TRIPS_708\	SAKNFYNISNENGDNTFNNNNNNMDNKKRMYNYNKHKDNNSRYTDNSNKNRDNSNKNRDNYNRNKDKNNTNRDNYNRYKDNNYYYNNSDNNNYNERKRYI	100
TRIPS_759\	SAKNFYNISNENGDNTFNNNNNNMDNKKRMYNYNKHKDNNSRYTDNSNKNRDNSNKNRDNYNRNKDKNNTNRDNYNRYKDNNYYYNNSDNNNYNERKRYI	100

+ Majority

Majority	RKKTYNKLSYFNLPSLKSIYNNKIKGNSEEFSFDNELPEQTESFPLNKPQDHEAFYNLKKHHTNVYEPNDEEKQNEQKLKDQIKITSDILYKDIEENKNT	
	110 120 130 140 150 160 170 180 190 200	
1.Pf3D7_10_v3\	RKKTYNKLSYFNLPSLKSIYNNKIKGNSEEFSFDNELPEQTESFPLNKPQDHEAFYNLKKHHTNVYEPNDEEKQNEQKLKDQIKITSDILYKDIEENKNT	200
1.AA_CAMBONDIA_KH01\	RKKTYNKLSYFNLPSLKSIYNNKIKGNSEEFSFDNELPEQTESFPLNKPQDHEAFYNLKKHHTNVYEPNDEEKQNEQKLKDQIKITSDILYKDIEENKNT	200
1.AA_LAOS_Dd2-1\	RKKTYNKLSYFNLPSLKSIYNNKIKGNSEEFSFDNELPEQTESFPLNKPQDHEAFYNLKKHHTNVYEPNDEEKQNEQKLKDQIKITSDILYKDIEENKNT	200
1.AA_LAOS_Dd2-2\	RKKTYNKLSYFNLPSLKSIYNNKIKGNSEEFSFDNELPEQTESFPLNKPQDHEAFYNLKKHHTNVYEPNDEEKQNEQKLKDQIKITSDILYKDIEENKNT	200
1.AA_THAI_DD2_WTSI\	RKKTYNKLSYFNLPSLKSIYNNKIKGNSEEFSFDNELPEQTESFPLNKPQDHEAFYNLKKHHTNVYEPNDEEKQNEQKLKDQIKITSDILYKDIEENKNT	200
1.AA_THAI_T9_94\	RKKTYNKLSYFNLPSLKSIYNNKIKGNSEEFSFDNELPEQTESFPLNKPQDHEAFYNLKKHHTNVYEPNDEEKQNEQKLKDQIKITSDILYKDIEENKNT	200
303.1\	RKKTYNKLSYFNLPSLKSIYNNKIKGNSEEFSFDNELPEQTESFPLNKPQDHEAFYNLKKHHTNVYEPNDEEKQNEQKLKDQIKITSDILYKDIEENKNT	200
309.1\	RKKTYNKLSYFNLPSLKSIYNNKIKGNSEEFSFDNELPEQTESFPLNKPQDHEAFYNLKKHHTNVYEPNDEEKQNEQKLKDQIKITSDILYKDIEENKNT	200
318.1\	RKKTYNKLSYFNLPSLKSIYNNKIKGNSEEFSFDNELPEQTESFPLNKPQDHEAFYNLKKHHTNVYEPNDEEKQNEQKLKDQIKITSDILYKDIEENKNT	200
326.1\	RKKTYNKLSYFNLPSLKSIYNNKIKGNSEEFSFDNELPEQTESFPLNKPQDHEAFYNLKKHHTNVYEPNDEEKQNEQKLKDQIKITSDILYKDIEENKNT	200
327.1\	RKKTYNKLSYFNLPSLKSIYNNKIKGNSEEFSFDNELPEQTESFPLNKPQDHEAFYNLKKHHTNVYEPNDEEKQNEQKLKDQIKITSDILYKDIEENKNT	200
365.1\	RKKTYNKLSYFNLPSLKSIYNNKIKGNSEEFSFDNELPEQTESFPLNKPQDHEAFYNLKKHHTNVYEPNDEEKQNEQKLKDQIKITSDILYKDIEENKNT	200
366.1\	RKKTYNKLSYFNLPSLKSIYNNKIKGNSEEFSFDNELPEQTESFPLNKPQDHEAFYNLKKHHTNVYEPNDEEKQNEQKLKDQIKITSDILYKDIEENKNT	200
377.1\	RKKTYNKLSYFNLPSLKSIYNNKIKGNSEEFSFDNELPEQTESFPLNKPQDHEAFYNLKKHHTNVYEPNDEEKQNEQKLKDQIKITSDILYKDIEENKNT	200
383.1\	RKKTYNKLSYFNLPSLKSIYNNKIKGNSEEFSFDNELPEQTESFPLNKPQDHEAFYNLKKHHTNVYEPNDEEKQNEQKLKDQIKITSDILYKDIEENKNT	200
397.1\	RKKTYNKLSYFNLPSLKSIYNNKIKGNSEEFSFDNELPEQTESFPLNKPQDHEAFYNLKKHHTNVYEPNDEEKQNEQKLKDQIKITSDILYKDIEENKNT	200
398.1\	RKKTYNKLSYFNLPSLKSIYNNKIKGNSEEFSFDNELPEQTESFPLNKPQDHEAFYNLKKHHTNVYEPNDEEKQNEQKLKDQIKITSDILYKDIEENKNT	200
3D7_WTSI\	RKKTYNKLSYFNLPSLKSIYNNKIKGNSEEFSFDNELPEQTESFPLNKPQDHEAFYNLKKHHTNVYEPNDEEKQNEQKLKDQIKITSDILYKDIEENKNT	200
58.1\	RKKTYNKLSYFNLPSLKSIYNNKIKGNSEEFSFDNELPEQTESFPLNKPQDHEAFYNLKKHHTNVYEPNDEEKQNEQKLKDQIKITSDILYKDIEENKNT	200
707A\	RKKTYNKLSYFNLPSLKSIYNNKIKGNSEEFSFDNELPEQTESFPLNKPQDHEAFYNLKKHHTNVYEPNDEEKQNEQKLKDQIKITSDILYKDIEENKNT	200
7G8_2\	RKKTYNKLSYFNLPSLKSIYNNKIKGNSEEFSFDNELPEQTESFPLNKPQDHEAFYNLKKHHTNVYEPNDEEKQNEQKLKDQIKITSDILYKDIEENKNT	200
7G8\	RKKTYNKLSYFNLPSLKSIYNNKIKGNSEEFSFDNELPEQTESFPLNKPQDHEAFYNLKKHHTNVYEPNDEEKQNEQKLKDQIKITSDILYKDIEENKNT	200
BM_0008\	RKKTYNKLSYFNLPSLKSIYNNKIKGNSEEFSFDNELPEQTESFPLNKPQDHEAFYNLKKHHTNVYEPNDEEKQNEQKLKDQIKITSDILYKDIEENKNT	200
BM_0009\	RKKTYNKLSYFNLPSLKSIYNNKIKGNSEEFSFDNELPEQTESFPLNKPQDHEAFYNLKKHHTNVYEPNDEEKQNEQKLKDQIKITSDILYKDIEENKNT	200
CS2\	RKKTYNKLSYFNLPSLKSIYNNKIKGNSEEFSFDNELPEQTESFPLNKPQDHEAFYNLKKHHTNVYEPNDEEKQNEQKLKDQIKITSDILYKDIEENKNT	200
G224\	RKKTYNKLSYFNLPSLKSIYNNKIKGNSEEFSFDNELPEQTESFPLNKPQDHEAFYNLKKHHTNVYEPNDEEKQNEQKLKDQIKITSDILYKDIEENKNT	200

Asian isolates

GA01\	RKKTYNKLSYFNLPSLKSIYNNKIKGNSEEFSFDNELPEQTESFPLNKPQDHEAFYNLKKHHTNVYEPNDEEKQNEQKLKDQIKITSDILYKDIEENKNT	200
GB4_WTSI\	RKKTYNKLSYFNLPSLKSIYNNKIKGNSEEFSFDNELPEQTESFPLNKPQDHEAFYNLKKHHTNVYEPNDEEKQNEQKLKDQIKITSDILYKDIEENKNT	200
GB4\	RKKTYNKLSYFNLPSLKSIYNNKIKGNSEEFSFDNELPEQTESFPLNKPQDHEAFYNLKKHHTNVYEPNDEEKQNEQKLKDQIKITSDILYKDIEENKNT	200
GN01\	RKKTYNKLSYFNLPSLKSIYNNKIKGNSEEFSFDNELPEQTESFPLNKPQDHEAFYNLKKHHTNVYEPNDEEKQNEQKLKDQIKITSDILYKDIEENKNT	200
H209\	RKKTYNKLSYFNLPSLKSIYNNKIKGNSEEFSFDNELPEQTESFPLNKPQDHEAFYNLKKHHTNVYEPNDEEKQNEQKLKDQIKITSDILYKDIEENKNT	200
HB3\	RKKTYNKLSYFNLPSLKSIYNNKIKGNSEEFSFDNELPEQTESFPLNKPQDHEAFYNLKKHHTNVYEPNDEEKQNEQKLKDQIKITSDILYKDIEENKNT	200
IT_WTSI\	RKKTYNKLSYFNLPSLKSIYNNKIKGNSEEFSFDNELPEQTESFPLNKPQDHEAFYNLKKHHTNVYEPNDEEKQNEQKLKDQIKITSDILYKDIEENKNT	200
KE01\	RKKTYNKLSYFNLPSLKSIYNNKIKGNSEEFSFDNELPEQTESFPLNKPQDHEAFYNLKKHHTNVYEPNDEEKQNEQKLKDQIKITSDILYKDIEENKNT	200
M219-D\	RKKTYNKLSYFNLPSLKSIYNNKIKGNSEEFSFDNELPEQTESFPLNKPQDHEAFYNLKKHHTNVYEPNDEEKQNEQKLKDQIKITSDILYKDIEENKNT	200
M271-D\	RKKTYNKLSYFNLPSLKSIYNNKIKGNSEEFSFDNELPEQTESFPLNKPQDHEAFYNLKKHHTNVYEPNDEEKQNEQKLKDQIKITSDILYKDIEENKNT	200
M312-D\	RKKTYNKLSYFNLPSLKSIYNNKIKGNSEEFSFDNELPEQTESFPLNKPQDHEAFYNLKKHHTNVYEPNDEEKQNEQKLKDQIKITSDILYKDIEENKNT	200
ML01\	RKKTYNKLSYFNLPSLKSIYNNKIKGNSEEFSFDNELPEQTESFPLNKPQDHEAFYNLKKHHTNVYEPNDEEKQNEQKLKDQIKITSDILYKDIEENKNT	200
N011-A\	RKKTYNKLSYFNLPSLKSIYNNKIKGNSEEFSFDNELPEQTESFPLNKPQDHEAFYNLKKHHTNVYEPNDEEKQNEQKLKDQIKITSDILYKDIEENKNT	200
N023-A\	RKKTYNKLSYFNLPSLKSIYNNKIKGNSEEFSFDNELPEQTESFPLNKPQDHEAFYNLKKHHTNVYEPNDEEKQNEQKLKDQIKITSDILYKDIEENKNT	200
N071-I\	RKKTYNKLSYFNLPSLKSIYNNKIKGNSEEFSFDNELPEQTESFPLNKPQDHEAFYNLKKHHTNVYEPNDEEKQNEQKLKDQIKITSDILYKDIEENKNT	200
N164-A\	RKKTYNKLSYFNLPSLKSIYNNKIKGNSEEFSFDNELPEQTESFPLNKPQDHEAFYNLKKHHTNVYEPNDEEKQNEQKLKDQIKITSDILYKDIEENKNT	200
N497-C\	RKKTYNKLSYFNLPSLKSIYNNKIKGNSEEFSFDNELPEQTESFPLNKPQDHEAFYNLKKHHTNVYEPNDEEKQNEQKLKDQIKITSDILYKDIEENKNT	200
N579-A\	RKKTYNKLSYFNLPSLKSIYNNKIKGNSEEFSFDNELPEQTESFPLNKPQDHEAFYNLKKHHTNVYEPNDEEKQNEQKLKDQIKITSDILYKDIEENKNT	200
O079-B\	RKKTYNKLSYFNLPSLKSIYNNKIKGNSEEFSFDNELPEQTESFPLNKPQDHEAFYNLKKHHTNVYEPNDEEKQNEQKLKDQIKITSDILYKDIEENKNT	200
O141-A\	RKKTYNKLSYFNLPSLKSIYNNKIKGNSEEFSFDNELPEQTESFPLNKPQDHEAFYNLKKHHTNVYEPNDEEKQNEQKLKDQIKITSDILYKDIEENKNT	200
O222-A\	RKKTYNKLSYFNLPSLKSIYNNKIKGNSEEFSFDNELPEQTESFPLNKPQDHEAFYNLKKHHTNVYEPNDEEKQNEQKLKDQIKITSDILYKDIEENKNT	200
O306-A\	RKKTYNKLSYFNLPSLKSIYNNKIKGNSEEFSFDNELPEQTESFPLNKPQDHEAFYNLKKHHTNVYEPNDEEKQNEQKLKDQIKITSDILYKDIEENKNT	200
O314\	RKKTYNKLSYFNLPSLKSIYNNKIKGNSEEFSFDNELPEQTESFPLNKPQDHEAFYNLKKHHTNVYEPNDEEKQNEQKLKDQIKITSDILYKDIEENKNT	200
P164-C\	RKKTYNKLSYFNLPSLKSIYNNKIKGNSEEFSFDNELPEQTESFPLNKPQDHEAFYNLKKHHTNVYEPNDEEKQNEQKLKDQIKITSDILYKDIEENKNT	200
P167-B\	RKKTYNKLSYFNLPSLKSIYNNKIKGNSEEFSFDNELPEQTESFPLNKPQDHEAFYNLKKHHTNVYEPNDEEKQNEQKLKDQIKITSDILYKDIEENKNT	200
P196J3-C\	RKKTYNKLSYFNLPSLKSIYNNKIKGNSEEFSFDNELPEQTESFPLNKPQDHEAFYNLKKHHTNVYEPNDEEKQNEQKLKDQIKITSDILYKDIEENKNT	200
P237-C\	RKKTYNKLSYFNLPSLKSIYNNKIKGNSEEFSFDNELPEQTESFPLNKPQDHEAFYNLKKHHTNVYEPNDEEKQNEQKLKDQIKITSDILYKDIEENKNT	200
P241-D\	RKKTYNKLSYFNLPSLKSIYNNKIKGNSEEFSFDNELPEQTESFPLNKPQDHEAFYNLKKHHTNVYEPNDEEKQNEQKLKDQIKITSDILYKDIEENKNT	200
PS097\	RKKTYNKLSYFNLPSLKSIYNNKIKGNSEEFSFDNELPEQTESFPLNKPQDHEAFYNLKKHHTNVYEPNDEEKQNEQKLKDQIKITSDILYKDIEENKNT	200
PS103\	RKKTYNKLSYFNLPSLKSIYNNKIKGNSEEFSFDNELPEQTESFPLNKPQDHEAFYNLKKHHTNVYEPNDEEKQNEQKLKDQIKITSDILYKDIEENKNT	200
PS122_G11\	RKKTYNKLSYFNLPSLKSIYNNKIKGNSEEFSFDNELPEQTESFPLNKPQDHEAFYNLKKHHTNVYEPNDEEKQNEQKLKDQIKITSDILYKDIEENKNT	200
PS149\	RKKTYNKLSYFNLPSLKSIYNNKIKGNSEEFSFDNELPEQTESFPLNKPQDHEAFYNLKKHHTNVYEPNDEEKQNEQKLKDQIKITSDILYKDIEENKNT	200
PS170\	RKKTYNKLSYFNLPSLKSIYNNKIKGNSEEFSFDNELPEQTESFPLNKPQDHEAFYNLKKHHTNVYEPNDEEKQNEQKLKDQIKITSDILYKDIEENKNT	200
PS183\	RKKTYNKLSYFNLPSLKSIYNNKIKGNSEEFSFDNELPEQTESFPLNKPQDHEAFYNLKKHHTNVYEPNDEEKQNEQKLKDQIKITSDILYKDIEENKNT	200
PS186\	RKKTYNKLSYFNLPSLKSIYNNKIKGNSEEFSFDNELPEQTESFPLNKPQDHEAFYNLKKHHTNVYEPNDEEKQNEQKLKDQIKITSDILYKDIEENKNT	200
PS189\	RKKTYNKLSYFNLPSLKSIYNNKIKGNSEEFSFDNELPEQTESFPLNKPQDHEAFYNLKKHHTNVYEPNDEEKQNEQKLKDQIKITSDILYKDIEENKNT	200
PS206_E11\	RKKTYNKLSYFNLPSLKSIYNNKIKGNSEEFSFDNELPEQTESFPLNKPQDHEAFYNLKKHHTNVYEPNDEEKQNEQKLKDQIKITSDILYKDIEENKNT	200
PS250\	RKKTYNKLSYFNLPSLKSIYNNKIKGNSEEFSFDNELPEQTESFPLNKPQDHEAFYNLKKHHTNVYEPNDEEKQNEQKLKDQIKITSDILYKDIEENKNT	200
RV_3600\	RKKTYNKLSYFNLPSLKSIYNNKIKGNSEEFSFDNELPEQTESFPLNKPQDHEAFYNLKKHHTNVYEPNDEEKQNEQKLKDQIKITSDILYKDIEENKNT	200
RV_3606\	RKKTYNKLSYFNLPSLKSIYNNKIKGNSEEFSFDNELPEQTESFPLNKPQDHEAFYNLKKHHTNVYEPNDEEKQNEQKLKDQIKITSDILYKDIEENKNT	200
RV_3610\	RKKTYNKLSYFNLPSLKSIYNNKIKGNSEEFSFDNELPEQTESFPLNKPQDHEAFYNLKKHHTNVYEPNDEEKQNEQKLKDQIKITSDILYKDIEENKNT	200
RV_3611\	RKKTYNKLSYFNLPSLKSIYNNKIKGNSEEFSFDNELPEQTESFPLNKPQDHEAFYNLKKHHTNVYEPNDEEKQNEQKLKDQIKITSDILYKDIEENKNT	200
RV_3614\	RKKTYNKLSYFNLPSLKSIYNNKIKGNSEEFSFDNELPEQTESFPLNKPQDHEAFYNLKKHHTNVYEPNDEEKQNEQKLKDQIKITSDILYKDIEENKNT	200
RV_3630\	RKKTYNKLSYFNLPSLKSIYNNKIKGNSEEFSFDNELPEQTESFPLNKPQDHEAFYNLKKHHTNVYEPNDEEKQNEQKLKDQIKITSDILYKDIEENKNT	200
RV_3635\	RKKTYNKLSYFNLPSLKSIYNNKIKGNSEEFSFDNELPEQTESFPLNKPQDHEAFYNLKKHHTNVYEPNDEEKQNEQKLKDQIKITSDILYKDIEENKNT	200
RV_3637\	RKKTYNKLSYFNLPSLKSIYNNKIKGNSEEFSFDNELPEQTESFPLNKPQDHEAFYNLKKHHTNVYEPNDEEKQNEQKLKDQIKITSDILYKDIEENKNT	200
RV_3642\	RKKTYNKLSYFNLPSLKSIYNNKIKGNSEEFSFDNELPEQTESFPLNKPQDHEAFYNLKKHHTNVYEPNDEEKQNEQKLKDQIKITSDILYKDIEENKNT	200
RV_3650\	RKKTYNKLSYFNLPSLKSIYNNKIKGNSEEFSFDNELPEQTESFPLNKPQDHEAFYNLKKHHTNVYEPNDEEKQNEQKLKDQIKITSDILYKDIEENKNT	200
RV_3655\	RKKTYNKLSYFNLPSLKSIYNNKIKGNSEEFSFDNELPEQTESFPLNKPQDHEAFYNLKKHHTNVYEPNDEEKQNEQKLKDQIKITSDILYKDIEENKNT	200
RV_3671\	RKKTYNKLSYFNLPSLKSIYNNKIKGNSEEFSFDNELPEQTESFPLNKPQDHEAFYNLKKHHTNVYEPNDEEKQNEQKLKDQIKITSDILYKDIEENKNT	200
RV_3672\	RKKTYNKLSYFNLPSLKSIYNNKIKGNSEEFSFDNELPEQTESFPLNKPQDHEAFYNLKKHHTNVYEPNDEEKQNEQKLKDQIKITSDILYKDIEENKNT	200
RV_3673\	RKKTYNKLSYFNLPSLKSIYNNKIKGNSEEFSFDNELPEQTESFPLNKPQDHEAFYNLKKHHTNVYEPNDEEKQNEQKLKDQIKITSDILYKDIEENKNT	200
RV_3675\	RKKTYNKLSYFNLPSLKSIYNNKIKGNSEEFSFDNELPEQTESFPLNKPQDHEAFYNLKKHHTNVYEPNDEEKQNEQKLKDQIKITSDILYKDIEENKNT	200
RV_3687\	RKKTYNKLSYFNLPSLKSIYNNKIKGNSEEFSFDNELPEQTESFPLNKPQDHEAFYNLKKHHTNVYEPNDEEKQNEQKLKDQIKITSDILYKDIEENKNT	200
RV_3695\	RKKTYNKLSYFNLPSLKSIYNNKIKGNSEEFSFDNELPEQTESFPLNKPQDHEAFYNLKKHHTNVYEPNDEEKQNEQKLKDQIKITSDILYKDIEENKNT	200
RV_3696\	RKKTYNKLSYFNLPSLKSIYNNKIKGNSEEFSFDNELPEQTESFPLNKPQDHEAFYNLKKHHTNVYEPNDEEKQNEQKLKDQIKITSDILYKDIEENKNT	200
RV_3701\	RKKTYNKLSYFNLPSLKSIYNNKIKGNSEEFSFDNELPEQTESFPLNKPQDHEAFYNLKKHHTNVYEPNDEEKQNEQKLKDQIKITSDILYKDIEENKNT	200
RV_3702\	RKKTYNKLSYFNLPSLKSIYNNKIKGNSEEFSFDNELPEQTESFPLNKPQDHEAFYNLKKHHTNVYEPNDEEKQNEQKLKDQIKITSDILYKDIEENKNT	200
RV_3703\	RKKTYNKLSYFNLPSLKSIYNNKIKGNSEEFSFDNELPEQTESFPLNKPQDHEAFYNLKKHHTNVYEPNDEEKQNEQKLKDQIKITSDILYKDIEENKNT	200
RV_3708\	RKKTYNKLSYFNLPSLKSIYNNKIKGNSEEFSFDNELPEQTESFPLNKPQDHEAFYNLKKHHTNVYEPNDEEKQNEQKLKDQIKITSDILYKDIEENKNT	200
RV_3714\	RKKTYNKLSYFNLPSLKSIYNNKIKGNSEEFSFDNELPEQTESFPLNKPQDHEAFYNLKKHHTNVYEPNDEEKQNEQKLKDQIKITSDILYKDIEENKNT	200
RV_3717\	RKKTYNKLSYFNLPSLKSIYNNKIKGNSEEFSFDNELPEQTESFPLNKPQDHEAFYNLKKHHTNVYEPNDEEKQNEQKLKDQIKITSDILYKDIEENKNT	200
RV_3729\	RKKTYNKLSYFNLPSLKSIYNNKIKGNSEEFSFDNELPEQTESFPLNKPQDHEAFYNLKKHHTNVYEPNDEEKQNEQKLKDQIKITSDILYKDIEENKNT	200
RV_3730\	RKKTYNKLSYFNLPSLKSIYNNKIKGNSEEFSFDNELPEQTESFPLNKPQDHEAFYNLKKHHTNVYEPNDEEKQNEQKLKDQIKITSDILYKDIEENKNT	200
RV_3731\	RKKTYNKLSYFNLPSLKSIYNNKIKGNSEEFSFDNELPEQTESFPLNKPQDHEAFYNLKKHHTNVYEPNDEEKQNEQKLKDQIKITSDILYKDIEENKNT	200
RV_3735\	RKKTYNKLSYFNLPSLKSIYNNKIKGNSEEFSFDNELPEQTESFPLNKPQDHEAFYNLKKHHTNVYEPNDEEKQNEQKLKDQIKITSDILYKDIEENKNT	200
RV_3736\	RKKTYNKLSYFNLPSLKSIYNNKIKGNSEEFSFDNELPEQTESFPLNKPQDHEAFYNLKKHHTNVYEPNDEEKQNEQKLKDQIKITSDILYKDIEENKNT	200
RV_3737\	RKKTYNKLSYFNLPSLKSIYNNKIKGNSEEFSFDNELPEQTESFPLNKPQDHEAFYNLKKHHTNVYEPNDEEKQNEQKLKDQIKITSDILYKDIEENKNT	200
RV_3739\	RKKTYNKLSYFNLPSLKSIYNNKIKGNSEEFSFDNELPEQTESFPLNKPQDHEAFYNLKKHHTNVYEPNDEEKQNEQKLKDQIKITSDILYKDIEENKNT	200
RV_3740\	RKKTYNKLSYFNLPSLKSIYNNKIKGNSEEFSFDNELPEQTESFPLNKPQDHEAFYNLKKHHTNVYEPNDEEKQNEQKLKDQIKITSDILYKDIEENKNT	200
RV_3741\	RKKTYNKLSYFNLPSLKSIYNNKIKGNSEEFSFDNELPEQTESFPLNKPQDHEAFYNLKKHHTNVYEPNDEEKQNEQKLKDQIKITSDILYKDIEENKNT	200
RV_3764\	RKKTYNKLSYFNLPSLKSIYNNKIKGNSEEFSFDNELPEQTESFPLNKPQDHEAFYNLKKHHTNVYEPNDEEKQNEQKLKDQIKITSDILYKDIEENKNT	200
RV_3766\	RKKTYNKLSYFNLPSLKSIYNNKIKGNSEEFSFDNELPEQTESFPLNKPQDHEAFYNLKKHHTNVYEPNDEEKQNEQKLKDQIKITSDILYKDIEENKNT	200
RV_3769\	RKKTYNKLSYFNLPSLKSIYNNKIKGNSEEFSFDNELPEQTESFPLNKPQDHEAFYNLKKHHTNVYEPNDEEKQNEQKLKDQIKITSDILYKDIEENKNT	200
SantaLucia_SalvadorI\	RKKTYNKLSYFNLPSLKSIYNNKIKGNSEEFSFDNELPEQTESFPLNKPQDHEAFYNLKKHHTNVYEPNDEEKQNEQKLKDQIKITSDILYKDIEENKNT	200
SD01\	RKKTYNKLSYFNLPSLKSIYNNKIKGNSEEFSFDNELPEQTESFPLNKPQDHEAFYNLKKHHTNVYEPNDEEKQNEQKLKDQIKITSDILYKDIEENKNT	200

SenT001.08\	RKKTYNKLSYFNLPSLKSIYNNKIKGNSEEFSFDNELPEQTESFPLNKPQDHEAFYNLKKHHTNVYEPNDEEKQNEQKLKDQIKITSDILYKDIEENKNT	200
SenT002.07\	RKKTYNKLSYFNLPSLKSIYNNKIKGNSEEFSFDNELPEQTESFPLNKPQDHEAFYNLKKHHTNVYEPNDEEKQNEQKLKDQIKITSDILYKDIEENKNT	200
SenT002.09\	RKKTYNKLSYFNLPSLKSIYNNKIKGNSEEFSFDNELPEQTESFPLNKPQDHEAFYNLKKHHTNVYEPNDEEKQNEQKLKDQIKITSDILYKDIEENKNT	200
SenT015.09\	RKKTYNKLSYFNLPSLKSIYNNKIKGNSEEFSFDNELPEQTESFPLNKPQDHEAFYNLKKHHTNVYEPNDEEKQNEQKLKDQIKITSDILYKDIEENKNT	200
SenT018.09\	RKKTYNKLSYFNLPSLKSIYNNKIKGNSEEFSFDNELPEQTESFPLNKPQDHEAFYNLKKHHTNVYEPNDEEKQNEQKLKDQIKITSDILYKDIEENKNT	200
SenT021.09\	RKKTYNKLSYFNLPSLKSIYNNKIKGNSEEFSFDNELPEQTESFPLNKPQDHEAFYNLKKHHTNVYEPNDEEKQNEQKLKDQIKITSDILYKDIEENKNT	200
SenT022.09\	RKKTYNKLSYFNLPSLKSIYNNKIKGNSEEFSFDNELPEQTESFPLNKPQDHEAFYNLKKHHTNVYEPNDEEKQNEQKLKDQIKITSDILYKDIEENKNT	200
SenT024.08\	RKKTYNKLSYFNLPSLKSIYNNKIKGNSEEFSFDNELPEQTESFPLNKPQDHEAFYNLKKHHTNVYEPNDEEKQNEQKLKDQIKITSDILYKDIEENKNT	200
SenT029.09\	RKKTYNKLSYFNLPSLKSIYNNKIKGNSEEFSFDNELPEQTESFPLNKPQDHEAFYNLKKHHTNVYEPNDEEKQNEQKLKDQIKITSDILYKDIEENKNT	200
SenT032.09\	RKKTYNKLSYFNLPSLKSIYNNKIKGNSEEFSFDNELPEQTESFPLNKPQDHEAFYNLKKHHTNVYEPNDEEKQNEQKLKDQIKITSDILYKDIEENKNT	200
SenT033.08\	RKKTYNKLSYFNLPSLKSIYNNKIKGNSEEFSFDNELPEQTESFPLNKPQDHEAFYNLKKHHTNVYEPNDEEKQNEQKLKDQIKITSDILYKDIEENKNT	200
SenT033.09\	RKKTYNKLSYFNLPSLKSIYNNKIKGNSEEFSFDNELPEQTESFPLNKPQDHEAFYNLKKHHTNVYEPNDEEKQNEQKLKDQIKITSDILYKDIEENKNT	200
SenT042.09\	RKKTYNKLSYFNLPSLKSIYNNKIKGNSEEFSFDNELPEQTESFPLNKPQDHEAFYNLKKHHTNVYEPNDEEKQNEQKLKDQIKITSDILYKDIEENKNT	200
SenT044.10\	RKKTYNKLSYFNLPSLKSIYNNKIKGNSEEFSFDNELPEQTESFPLNKPQDHEAFYNLKKHHTNVYEPNDEEKQNEQKLKDQIKITSDILYKDIEENKNT	200
SenT046.10\	RKKTYNKLSYFNLPSLKSIYNNKIKGNSEEFSFDNELPEQTESFPLNKPQDHEAFYNLKKHHTNVYEPNDEEKQNEQKLKDQIKITSDILYKDIEENKNT	200
SenT047.09\	RKKTYNKLSYFNLPSLKSIYNNKIKGNSEEFSFDNELPEQTESFPLNKPQDHEAFYNLKKHHTNVYEPNDEEKQNEQKLKDQIKITSDILYKDIEENKNT	200
SenT061.09\	RKKTYNKLSYFNLPSLKSIYNNKIKGNSEEFSFDNELPEQTESFPLNKPQDHEAFYNLKKHHTNVYEPNDEEKQNEQKLKDQIKITSDILYKDIEENKNT	200
SenT063.07\	RKKTYNKLSYFNLPSLKSIYNNKIKGNSEEFSFDNELPEQTESFPLNKPQDHEAFYNLKKHHTNVYEPNDEEKQNEQKLKDQIKITSDILYKDIEENKNT	200
SenT066.08\	RKKTYNKLSYFNLPSLKSIYNNKIKGNSEEFSFDNELPEQTESFPLNKPQDHEAFYNLKKHHTNVYEPNDEEKQNEQKLKDQIKITSDILYKDIEENKNT	200
SenT067.09\	RKKTYNKLSYFNLPSLKSIYNNKIKGNSEEFSFDNELPEQTESFPLNKPQDHEAFYNLKKHHTNVYEPNDEEKQNEQKLKDQIKITSDILYKDIEENKNT	200
SenT075.10\	RKKTYNKLSYFNLPSLKSIYNNKIKGNSEEFSFDNELPEQTESFPLNKPQDHEAFYNLKKHHTNVYEPNDEEKQNEQKLKDQIKITSDILYKDIEENKNT	200
SenT077.08\	RKKTYNKLSYFNLPSLKSIYNNKIKGNSEEFSFDNELPEQTESFPLNKPQDHEAFYNLKKHHTNVYEPNDEEKQNEQKLKDQIKITSDILYKDIEENKNT	200
SenT087.08\	RKKTYNKLSYFNLPSLKSIYNNKIKGNSEEFSFDNELPEQTESFPLNKPQDHEAFYNLKKHHTNVYEPNDEEKQNEQKLKDQIKITSDILYKDIEENKNT	200
SenT090.08\	RKKTYNKLSYFNLPSLKSIYNNKIKGNSEEFSFDNELPEQTESFPLNKPQDHEAFYNLKKHHTNVYEPNDEEKQNEQKLKDQIKITSDILYKDIEENKNT	200
SenT090.09\	RKKTYNKLSYFNLPSLKSIYNNKIKGNSEEFSFDNELPEQTESFPLNKPQDHEAFYNLKKHHTNVYEPNDEEKQNEQKLKDQIKITSDILYKDIEENKNT	200
SenT093.09\	RKKTYNKLSYFNLPSLKSIYNNKIKGNSEEFSFDNELPEQTESFPLNKPQDHEAFYNLKKHHTNVYEPNDEEKQNEQKLKDQIKITSDILYKDIEENKNT	200
SenT094.09\	RKKTYNKLSYFNLPSLKSIYNNKIKGNSEEFSFDNELPEQTESFPLNKPQDHEAFYNLKKHHTNVYEPNDEEKQNEQKLKDQIKITSDILYKDIEENKNT	200
SenT097.09\	RKKTYNKLSYFNLPSLKSIYNNKIKGNSEEFSFDNELPEQTESFPLNKPQDHEAFYNLKKHHTNVYEPNDEEKQNEQKLKDQIKITSDILYKDIEENKNT	200
SenT101.09\	RKKTYNKLSYFNLPSLKSIYNNKIKGNSEEFSFDNELPEQTESFPLNKPQDHEAFYNLKKHHTNVYEPNDEEKQNEQKLKDQIKITSDILYKDIEENKNT	200
SenT102.08\	RKKTYNKLSYFNLPSLKSIYNNKIKGNSEEFSFDNELPEQTESFPLNKPQDHEAFYNLKKHHTNVYEPNDEEKQNEQKLKDQIKITSDILYKDIEENKNT	200
SenT106.08\	RKKTYNKLSYFNLPSLKSIYNNKIKGNSEEFSFDNELPEQTESFPLNKPQDHEAFYNLKKHHTNVYEPNDEEKQNEQKLKDQIKITSDILYKDIEENKNT	200
SenT113.09\	RKKTYNKLSYFNLPSLKSIYNNKIKGNSEEFSFDNELPEQTESFPLNKPQDHEAFYNLKKHHTNVYEPNDEEKQNEQKLKDQIKITSDILYKDIEENKNT	200
SenT119.09\	RKKTYNKLSYFNLPSLKSIYNNKIKGNSEEFSFDNELPEQTESFPLNKPQDHEAFYNLKKHHTNVYEPNDEEKQNEQKLKDQIKITSDILYKDIEENKNT	200
SenT123.09\	RKKTYNKLSYFNLPSLKSIYNNKIKGNSEEFSFDNELPEQTESFPLNKPQDHEAFYNLKKHHTNVYEPNDEEKQNEQKLKDQIKITSDILYKDIEENKNT	200
SenT127.09\	RKKTYNKLSYFNLPSLKSIYNNKIKGNSEEFSFDNELPEQTESFPLNKPQDHEAFYNLKKHHTNVYEPNDEEKQNEQKLKDQIKITSDILYKDIEENKNT	200
SenT128.08\	RKKTYNKLSYFNLPSLKSIYNNKIKGNSEEFSFDNELPEQTESFPLNKPQDHEAFYNLKKHHTNVYEPNDEEKQNEQKLKDQIKITSDILYKDIEENKNT	200
SenT135.09\	RKKTYNKLSYFNLPSLKSIYNNKIKGNSEEFSFDNELPEQTESFPLNKPQDHEAFYNLKKHHTNVYEPNDEEKQNEQKLKDQIKITSDILYKDIEENKNT	200
SenT137.08\	RKKTYNKLSYFNLPSLKSIYNNKIKGNSEEFSFDNELPEQTESFPLNKPQDHEAFYNLKKHHTNVYEPNDEEKQNEQKLKDQIKITSDILYKDIEENKNT	200
SenT137.09\	RKKTYNKLSYFNLPSLKSIYNNKIKGNSEEFSFDNELPEQTESFPLNKPQDHEAFYNLKKHHTNVYEPNDEEKQNEQKLKDQIKITSDILYKDIEENKNT	200
SenT139.08\	RKKTYNKLSYFNLPSLKSIYNNKIKGNSEEFSFDNELPEQTESFPLNKPQDHEAFYNLKKHHTNVYEPNDEEKQNEQKLKDQIKITSDILYKDIEENKNT	200
SenT140.08\	RKKTYNKLSYFNLPSLKSIYNNKIKGNSEEFSFDNELPEQTESFPLNKPQDHEAFYNLKKHHTNVYEPNDEEKQNEQKLKDQIKITSDILYKDIEENKNT	200
SenT142.09\	RKKTYNKLSYFNLPSLKSIYNNKIKGNSEEFSFDNELPEQTESFPLNKPQDHEAFYNLKKHHTNVYEPNDEEKQNEQKLKDQIKITSDILYKDIEENKNT	200
SenT144.08\	RKKTYNKLSYFNLPSLKSIYNNKIKGNSEEFSFDNELPEQTESFPLNKPQDHEAFYNLKKHHTNVYEPNDEEKQNEQKLKDQIKITSDILYKDIEENKNT	200
SenT145.08\	RKKTYNKLSYFNLPSLKSIYNNKIKGNSEEFSFDNELPEQTESFPLNKPQDHEAFYNLKKHHTNVYEPNDEEKQNEQKLKDQIKITSDILYKDIEENKNT	200
SenT149.09\	RKKTYNKLSYFNLPSLKSIYNNKIKGNSEEFSFDNELPEQTESFPLNKPQDHEAFYNLKKHHTNVYEPNDEEKQNEQKLKDQIKITSDILYKDIEENKNT	200
SenT150.09\	RKKTYNKLSYFNLPSLKSIYNNKIKGNSEEFSFDNELPEQTESFPLNKPQDHEAFYNLKKHHTNVYEPNDEEKQNEQKLKDQIKITSDILYKDIEENKNT	200
SenT151.09\	RKKTYNKLSYFNLPSLKSIYNNKIKGNSEEFSFDNELPEQTESFPLNKPQDHEAFYNLKKHHTNVYEPNDEEKQNEQKLKDQIKITSDILYKDIEENKNT	200
SenT170.08\	RKKTYNKLSYFNLPSLKSIYNNKIKGNSEEFSFDNELPEQTESFPLNKPQDHEAFYNLKKHHTNVYEPNDEEKQNEQKLKDQIKITSDILYKDIEENKNT	200
SenT175.08\	RKKTYNKLSYFNLPSLKSIYNNKIKGNSEEFSFDNELPEQTESFPLNKPQDHEAFYNLKKHHTNVYEPNDEEKQNEQKLKDQIKITSDILYKDIEENKNT	200
SenT179.08\	RKKTYNKLSYFNLPSLKSIYNNKIKGNSEEFSFDNELPEQTESFPLNKPQDHEAFYNLKKHHTNVYEPNDEEKQNEQKLKDQIKITSDILYKDIEENKNT	200
SenT180.08\	RKKTYNKLSYFNLPSLKSIYNNKIKGNSEEFSFDNELPEQTESFPLNKPQDHEAFYNLKKHHTNVYEPNDEEKQNEQKLKDQIKITSDILYKDIEENKNT	200
SenT190.08\	RKKTYNKLSYFNLPSLKSIYNNKIKGNSEEFSFDNELPEQTESFPLNKPQDHEAFYNLKKHHTNVYEPNDEEKQNEQKLKDQIKITSDILYKDIEENKNT	200
SenT197.08\	RKKTYNKLSYFNLPSLKSIYNNKIKGNSEEFSFDNELPEQTESFPLNKPQDHEAFYNLKKHHTNVYEPNDEEKQNEQKLKDQIKITSDILYKDIEENKNT	200
SenT224.08\	RKKTYNKLSYFNLPSLKSIYNNKIKGNSEEFSFDNELPEQTESFPLNKPQDHEAFYNLKKHHTNVYEPNDEEKQNEQKLKDQIKITSDILYKDIEENKNT	200
SenT227.08\	RKKTYNKLSYFNLPSLKSIYNNKIKGNSEEFSFDNELPEQTESFPLNKPQDHEAFYNLKKHHTNVYEPNDEEKQNEQKLKDQIKITSDILYKDIEENKNT	200
SenT230.08\	RKKTYNKLSYFNLPSLKSIYNNKIKGNSEEFSFDNELPEQTESFPLNKPQDHEAFYNLKKHHTNVYEPNDEEKQNEQKLKDQIKITSDILYKDIEENKNT	200
SenT235.08\	RKKTYNKLSYFNLPSLKSIYNNKIKGNSEEFSFDNELPEQTESFPLNKPQDHEAFYNLKKHHTNVYEPNDEEKQNEQKLKDQIKITSDILYKDIEENKNT	200
SenT238.08\	RKKTYNKLSYFNLPSLKSIYNNKIKGNSEEFSFDNELPEQTESFPLNKPQDHEAFYNLKKHHTNVYEPNDEEKQNEQKLKDQIKITSDILYKDIEENKNT	200
SEV2_H5\	RKKTYNKLSYFNLPSLKSIYNNKIKGNSEEFSFDNELPEQTESFPLNKPQDHEAFYNLKKHHTNVYEPNDEEKQNEQKLKDQIKITSDILYKDIEENKNT	200
SN01\	RKKTYNKLSYFNLPSLKSIYNNKIKGNSEEFSFDNELPEQTESFPLNKPQDHEAFYNLKKHHTNVYEPNDEEKQNEQKLKDQIKITSDILYKDIEENKNT	200
TG01\	RKKTYNKLSYFNLPSLKSIYNNKIKGNSEEFSFDNELPEQTESFPLNKPQDHEAFYNLKKHHTNVYEPNDEEKQNEQKLKDQIKITSDILYKDIEENKNT	200
TRIPS_301\	RKKTYNKLSYFNLPSLKSIYNNKIKGNSEEFSFDNELPEQTESFPLNKPQDHEAFYNLKKHHTNVYEPNDEEKQNEQKLKDQIKITSDILYKDIEENKNT	200
TRIPS_303\	RKKTYNKLSYFNLPSLKSIYNNKIKGNSEEFSFDNELPEQTESFPLNKPQDHEAFYNLKKHHTNVYEPNDEEKQNEQKLKDQIKITSDILYKDIEENKNT	200
TRIPS_331\	RKKTYNKLSYFNLPSLKSIYNNKIKGNSEEFSFDNELPEQTESFPLNKPQDHEAFYNLKKHHTNVYEPNDEEKQNEQKLKDQIKITSDILYKDIEENKNT	200
TRIPS_355\	RKKTYNKLSYFNLPSLKSIYNNKIKGNSEEFSFDNELPEQTESFPLNKPQDHEAFYNLKKHHTNVYEPNDEEKQNEQKLKDQIKITSDILYKDIEENKNT	200
TRIPS_364\	RKKTYNKLSYFNLPSLKSIYNNKIKGNSEEFSFDNELPEQTESFPLNKPQDHEAFYNLKKHHTNVYEPNDEEKQNEQKLKDQIKITSDILYKDIEENKNT	200
TRIPS_373\	RKKTYNKLSYFNLPSLKSIYNNKIKGNSEEFSFDNELPEQTESFPLNKPQDHEAFYNLKKHHTNVYEPNDEEKQNEQKLKDQIKITSDILYKDIEENKNT	200
TRIPS_410\	RKKTYNKLSYFNLPSLKSIYNNKIKGNSEEFSFDNELPEQTESFPLNKPQDHEAFYNLKKHHTNVYEPNDEEKQNEQKLKDQIKITSDILYKDIEENKNT	200
TRIPS_433\	RKKTYNKLSYFNLPSLKSIYNNKIKGNSEEFSFDNELPEQTESFPLNKPQDHEAFYNLKKHHTNVYEPNDEEKQNEQKLKDQIKITSDILYKDIEENKNT	200
TRIPS_437\	RKKTYNKLSYFNLPSLKSIYNNKIKGNSEEFSFDNELPEQTESFPLNKPQDHEAFYNLKKHHTNVYEPNDEEKQNEQKLKDQIKITSDILYKDIEENKNT	200
TRIPS_440\	RKKTYNKLSYFNLPSLKSIYNNKIKGNSEEFSFDNELPEQTESFPLNKPQDHEAFYNLKKHHTNVYEPNDEEKQNEQKLKDQIKITSDILYKDIEENKNT	200
TRIPS_456\	RKKTYNKLSYFNLPSLKSIYNNKIKGNSEEFSFDNELPEQTESFPLNKPQDHEAFYNLKKHHTNVYEPNDEEKQNEQKLKDQIKITSDILYKDIEENKNT	200
TRIPS_461\	RKKTYNKLSYFNLPSLKSIYNNKIKGNSEEFSFDNELPEQTESFPLNKPQDHEAFYNLKKHHTNVYEPNDEEKQNEQKLKDQIKITSDILYKDIEENKNT	200
TRIPS_467\	RKKTYNKLSYFNLPSLKSIYNNKIKGNSEEFSFDNELPEQTESFPLNKPQDHEAFYNLKKHHTNVYEPNDEEKQNEQKLKDQIKITSDILYKDIEENKNT	200
TRIPS_470\	RKKTYNKLSYFNLPSLKSIYNNKIKGNSEEFSFDNELPEQTESFPLNKPQDHEAFYNLKKHHTNVYEPNDEEKQNEQKLKDQIKITSDILYKDIEENKNT	200
TRIPS_474\	RKKTYNKLSYFNLPSLKSIYNNKIKGNSEEFSFDNELPEQTESFPLNKPQDHEAFYNLKKHHTNVYEPNDEEKQNEQKLKDQIKITSDILYKDIEENKNT	200

Sequence	Alignment	Position
TRIPS_480\	RKKTYNKLSYFNLPSLKSIYNNKIKGNSEEFSFDNELPEQTESFPLNKPQDHEAFYNLKKHHTNVYKPNDEEKQNEQKLKDQIKITSDILYKDIEENKNT	200
TRIPS_482\	RKKTYNKLSYFNLPSLKSIYNNKIKGNSEEFSFDNELPEQTESFPLNKPQDHEAFYNLKKHHTNVYEPNDEEKQNEQKLKDQIKITSDILYKDIEENKNT	200
TRIPS_487\	RKKTYNKLSYFNLPSLKSIYNNKIKGNSEEFSFDNELPEQTESFPLNKPQDHEAFYNLKKHHTNVYEPNDEEKQNEQKLKDQIKITSDILYKDIEENKNT	200
TRIPS_490\	RKKTYNKLSYFNLPSLKSIYNNKIKGNSEEFSFDNELPEQTESFPLNKPQDHEAFYNLKKHHTNVYEPNDEEKQNEQKLKDQIKITSDILYKDIEENKNT	200
TRIPS_499\	RKKTYNKLSYFNLPSLKSIYNNKIKGNSEEFSFDNELPEQTESFPLNKPQDHEAFYNLKKHHTNVYEPNDEEKQNEQKLKDQIKITSDILYKDIEENKNT	200
TRIPS_501\	RKKTYNKLSYFNLPSLKSIYNNKIKGNSEEFSFDNELPEQTESFPLNKPQDHEAFYNLKKHHTNVYEPNDEEKQNEQKLKDQIKITSDILYKDIEENKNT	200
TRIPS_504\	RKKTYNKLSYFNLPSLKSIYNNKIKGNSEEFSFDNELPEQTESFPLNKPQDHEAFYNLKKHHTNVYEPNDEEKQNEQKLKDQIKITSDILYKDIEENKNT	200
TRIPS_700\	RKKTYNKLSYFNLPSLKSIYNNKIKGNSEEFSFDNELPEQTESFPLNKPQDHEAFYNLKKHHTNVYEPNDEEKQNEQKLKDQIKITSDILYKDIEENKNT	200
TRIPS_704\	RKKTYNKLSYFNLPSLKSIYNNKIKGNSEEFSFDNELPEQTESFPLNKPQDHEAFYNLKKHHTNVYEPNDEEKQNEQKLKDQIKITSDILYKDIEENKNT	200
TRIPS_708\	RKKTYNKLSYFNLPSLKSIYNNKIKGNSEEFSFDNELPEQTESFPLNKPQDHEAFYNLKKHHTNVYEPNDEEKQNEQKLKDQIKITSDILYKDIEENKNT	200
TRIPS_759\	RKKTYNKLSYFNLPSLKSIYNNKIKGNSEEFSFDNELPEQTESFPLNKPQDHEAFYNLKKHHTNVYEPNDEEKQNEQKLKDQIKITSDILYKDIEENKNT	200

+ Majority

Sequence	Alignment (210 220 230 240 250 260 270 280 290)	Position
Majority	EDVLLIETITINNGTTSNTIENNKDSNKEAENSNTEQNDNNNNDNNNNINNNNNNNDNKEEDMNENNNNSKVTGDSVENINEQTNNNQYP	
1.Pf3D7_10_v3\	EDVLLIETITINNGTTSNTIENNKDSNKEAENSNTEQNDNNNNDNNNNINNNNNNNDNKEEDMNENNNNSKVTGDSVENINEQTNNNQYP	290
1.AA_CAMBONDIA_KH01\	EDVLLIETITINNGTTSNTIENNKDSNKEAENSNTEQNDNNNNDNNNNINNNNNNNDNKEEDMNENNNNSKVTGDSVENINEQTNNNQYP	290
1.AA_LAOS_Dd2-1\	EDVLLIETITINNGTTSNTIENNKDSNKEAENSNTEQNDNNNNDNNNNINNNNNNNDNKEEDMNENNNNSKVTGDSVENINEQTNNNQYP	290
1.AA_LAOS_Dd2-2\	EDVLLIETITINNGTTSNTIENNKDSNKEAENSNTEQNDNNNNDNNNNINNNNNNNDNKEEDMNENNNNSKVTGDSVENINEQTNNNQYP	290
1.AA_THAI_DD2_WTSI\	EDVLLIETITINNGTTSNTIENNKDSNKEAENSNTEQNDNNNNDNNNNINNNNNNNDNKEEDMNENNNNSKVTGDSVENINEQTNNNQYP	290
1.AA_THAI_T9_94\	EDVLLIETITINNGTTSNTIENNKDSNKEAENSNTEQNDNNNNDNNNNINNNNNNNDNKEEDMNENNNNSKVTGDSVENINEQTNNNQYP	290
303.1\	EDVLLIETITINNGTTSNTIENNKDSNKEAENSNTEQNDNNNNDNNNNINNNNNNNDNKEEDMNENNNNSKVTGDSVENINEQTNNNQYP	290
309.1\	EDVLLIETITINNGTTSNTIENNKDSNKEAENSNTEQNDNNNNDNNNNINNNNNNNDNKEEDMNENNNNSKVTGDSVENINEQTNNNQYP	290
318.1\	EDVLLIETITINNGTTSNTIENNKDSNKEAENSNTEQNDNNNNDNNNNINNNNNNNDNKEEDMNENNNNSKVTGDSVENINEQTNNNQYP	290
326.1\	EDVLLIETITINNGTTSNTIENNKDSNKEAENSNTEQNDNNNNDNNNNINNNNNNNDNKEEDMNENNNNSKVTGDSVENINEQTNNNQYP	290
327.1\	EDVLLIETITINNGTTSNTIENNKDSNKEAENSNTEQNDNNNNDNNNNINNNNNNNDNKEEDMNENNNNSKVTGDSVENINEQTNNNQYP	290
365.1\	EDVLLIETITINNGTTSNTIENNKDSNKEAENSNTEQNDNNNNDNNNNINNNNNNNDNKEEDMNENNNNSKVTGDSVENINEQTNNNQYP	290
366.1\	EDVLLIETITINNGTTSNTIENNKDSNKEAENSNTEQNDNNNNDNNNNINNNNNNNDNKEEDMNENNNNSKVTGDSVENINEQTNNNQYP	290
377.1\	EDVLLIETITINNGTTSNTIENNKDSNKEAENSNTEQNDNNNNDNNNNINNNNNNNDNKEEDMNENNNNSKVTGDSVENINEQTNNNQYP	290
383.1\	EDVLLIETITINNGTTSNTIENNKDSNKEAENSNTEQNDNNNNDNNNNINNNNNNNDNKEEDMNENNNNSKVTGDSVENINEQTNNNQYP	290
397.1\	EDVLLIETITINNGTTSNTIENNKDSNKEAENSNTEQNDNNNNDNNNNINNNNNNNDNKEEDMNENNNNSKVTGDSVENINEQTNNNQYP	290
398.1\	EDVLLIETITINNGTTSNTIENNKDSNKEAENSNTEQNDNNNNDNNNNINNNNNNNDNKEEDMNENNNNSKVTGDSVENINEQTNNNQYP	290
3D7_WTSI\	EDVLLIETITINNGTTSNTIENNKDSNKEAENSNTEQNDNNNNDNNNNINNNNNNNDNKEEDMNENNNNSKVTGDSVENINEQTNNNQYP	290
58.1\	EDVLLIETITINNGTTSNTIENNKDSNKEAENSNTEQNDNNNNDNNNNINNNNNNNDNKEEDMNENNNNSKVTGDSVENINEQTNNNQYP	290
707A\	EDVLLIETITINNGTTSNTIENNKDSNKEAENSNTEQNDNNNNDNNNNINNNNNNNDNKEEDMNENNNNSKVTGDSVENINEQTNNNQYP	290
7G8_2\	EDVLLIETITINNGTTSNTIENNKDSNKEAENSNTEQNDNNNNDNNNNINNNNNNNDNKEEDMNENNNNSKVTGDSVENINEQTNNNQYP	290
7G8\	EDVLLIETITINNGTTSNTIENNKDSNKEAENSNTEQNDNNNNDNNNNINNNNNNNDNKEEDMNENNNNSKVTGDSVENINEQTNNNQYP	290
BM_0008\	EDVLLIETITINNGTTSNTIENNKDSNKEAENSNTEQNDNNNNDNNNNINNNNNNNDNKEEDMNENNNNSKVTGDSVENINEQTNNNQYP	290
BM_0009\	EDVLLIETITINNGTTSNTIENNKDSNKEAENSNTEQNDNNNNDNNNNINNNNNNNDNKEEDMNENNNNSKVTGDSVENINEQTNNNQYP	290
CS2\	EDVLLIETITINNGTTSNTIENNKDSNKEAENSNTEQNDNNNNDNNNNINNNNNNNDNKEEDMNENNNNSKVTGDSVENINEQTNNNQYP	290
G224\	EDVLLIETITINNGTTSNTIENNKDSNKEAENSNTEQNDNNNNDNNNNINNNNNNNDNKEEDMNENNNNSKVTGDSVENINEQTNNNQYP	290
GA01\	EDVLLIETITINNGTTSNTIENNKDSNKEAENSNTEQNDNNNNDNNNNINNNNNNNDNKEEDMNENNNNSKVTGDSVENINEQTNNNQYP	290
GB4_WTSI\	EDVLLIETITINNGTTSNTIENNKDSNKEAENSNTEQNDNNNNDNNNNINNNNNNNDNKEEDMNENNNNSKVTGDSVENINEQTNNNQYP	290
GB4\	EDVLLIETITINNGTTSNTIENNKDSNKEAENSNTEQNDNNNNDNNNNINNNNNNNDNKEEDMNENNNNSKVTGDSVENINEQTNNNQYP	290
GN01\	EDVLLIETITINNGTTSNTIENNKDSNKEAENSNTEQNDNNNNDNNNNINNNNNNNDNKEEDMNENNNNSKVTGDSVENINEQTNNNQYP	290
H209\	EDVLLIETITINNGTTSNTIENNKDSNKEAENSNTEQNDNNNNDNNNNINNNNNNNDNKEEDMNENNNNSKVTGDSVENINEQTNNNQYP	290
HB3\	EDVLLIETITINNGTTSNTIENNKDSNKEAENSNTEQNDNNNNDNNNNINNNNNNNDNKEEDMNENNNNSKVTGDSVENINEQTNNNQYP	290
IT_WTSI\	EDVLLIETITINNGTTSNTIENNKDSNKEAENSNTEQNDNNNNDNNNNINNNNNNNDNKEEDMNENNNNSKVTGDSVENINEQTNNNQYP	290
KE01\	EDVLLIETITINNGTTSNTIENNKDSNKEAENSNTEQNDNNNNDNNNNINNNNNNNDNKEEDMNENNNNSKVTGDSVENINEQTNNNQYP	290
M219-D\	EDVLLIETITINNGTTSNTIENNKDSNKEAENSNTEQNDNNNNDNNNNINNNNNNNDNKEEDMNENNNNSKVTGDSVENINEQTNNNQYP	290
M271-D\	EDVLLIETITINNGTTSNTIENNKDSNKEAENSNTEQNDNNNNDNNNNINNNNNNNDNKEEDMNENNNNSKVTGDSVENINEQTNNNQYP	290
M312-D\	EDVLLIETITINNGTTSNTIENNKDSNKEAENSNTEQNDNNNNDNNNNINNNNNNNDNKEEDMNENNNNSKVTGDSVENINEQTNNNQYP	290
ML01\	EDVLLIETITINNGTTSNTIENNKDSNKEAENSNTEQNDNNNNDNNNNINNNNNNNDNKEEDMNENNNNSKVTGDSVENINEQTNNNQYP	290
N011-A\	EDVLLIETITINNGTTSNTIENNKDSNKEAENSNTEQNDNNNNDNNNNINNNNNNNDNKEEDMNENNNNSKVTGDSVENINEQTNNNQYP	290
N023-A\	EDVLLIETITINNGTTSNTIENNKDSNKEAENSNTEQNDNNNNDNNNNINNNNNNNDNKEEDMNENNNNSKVTGDSVENINEQTNNNQYP	290
N071-I\	EDVLLIETITINNGTTSNTIENNKDSNKEAENSNTEQNDNNNNDNNNNINNNNNNNDNKEEDMNENNNNSKVTGDSVENINEQTNNNQYP	290
N164-A\	EDVLLIETITINNGTTSNTIENNKDSNKEAENSNTEQNDNNNNDNNNNINNNNNNNDNKEEDMNENNNNSKVTGDSVENINEQTNNNQYP	290
N497-C\	EDVLLIETITINNGTTSNTIENNKDSNKEAENSNTEQNDNNNNDNNNNINNNNNNNDNKEEDMNENNNNSKVTGDSVENINEQTNNNQYP	290
N579-A\	EDVLLIETITINNGTTSNTIENNKDSNKEAENSNTEQNDNNNNDNNNNINNNNNNNDNKEEDMNENNNNSKVTGDSVENINEQTNNNQYP	290
O079-B\	EDVLLIETITINNGTTSNTIENNKDSNKEAENSNTEQNDNNNNDNNNNINNNNNNNDNKEEDMNENNNNSKVTGDSVENINEQTNNNQYP	290
O141-A\	EDVLLIETITINNGTTSNTIENNKDSNKEAENSNTEQNDNNNNDNNNNINNNNNNNDNKEEDMNENNNNSKVTGDSVENINEQTNNNQYP	290
O222-A\	EDVLLIETITINNGTTSNTIENNKDSNKEAENSNTEQNDNNNNDNNNNINNNNNNNDNKEEDMNENNNNSKVTGDSVENINEQTNNNQYP	290
O306-A\	EDVLLIETITINNGTTSNTIENNKDSNKEAENSNTEQNDNNNNDNNNNINNNNNNNDNKEEDMNENNNNSKVTGDSVENINEQTNNNQYP	290
O314\	EDVLLIETITINNGTTSNTIENNKDSNKEAENSNTEQNDNNNNDNNNNINNNNNNNDNKEEDMNENNNNSKVTGDSVENINEQTNNNQYP	290
P164-C\	EDVLLIETITINNGTTSNTIENNKDSNKEAENSNTEQNDNNNNDNNNNINNNNNNNDNKEEDMNENNNNSKVTGDSVENINEQTNNNQYP	290
P167-B\	EDVLLIETITINNGTTSNTIENNKDSNKEAENSNTEQNDNNNNDNNNNINNNNNNNDNKEEDMNENNNNSKVTGDSVENINEQTNNNQYP	290
P196J3-C\	EDVLLIETITINNGTTSNTIENNKDSNKEAENSNTEQNDNNNNDNNNNINNNNNNNDNKEEDMNENNNNSKVTGDSVENINEQTNNNQYP	290
P237-C\	EDVLLIETITINNGTTSNTIENNKDSNKEAENSNTEQNDNNNNDNNNNINNNNNNNDNKEEDMNENNNNSKVTGDSVENINEQTNNNQYP	290
P241-D\	EDVLLIETITINNGTTSNTIENNKDSNKEAENSNTEQNDNNNNDNNNNINNNNNNNDNKEEDMNENNNNSKVTGDSVENINEQTNNNQYP	290
PS097\	EDVLLIETITINNGTTSNTIENNKDSNKEAENSNTEQNDNNNNDNNNNINNNNNNNDNKEEDMNENNNNSKVTGDSVENINEQTNNNQYP	290
PS103\	EDVLLIETITINNGTTSNTIENNKDSNKEAENSNTEQNDNNNNDNNNNINNNNNNNDNKEEDMNENNNNSKVTGDSVENINEQTNNNQYP	290
PS122_G11\	EDVLLIETITINNGTTSNTIENNKDSNKEAENSNTEQNDNNNNDNNNNINNNNNNNDNKEEDMNENNNNSKVTGDSVENINEQTNNNQYP	290
PS149\	EDVLLIETITINNGTTSNTIENNKDSNKEAENSNTEQNDNNNNDNNNNINNNNNNNDNKEEDMNENNNNSKVTGDSVENINEQTNNNQYP	290

Asian isolates

PS170\	EDVLLIETITINNGTTSNTIENNKDSNKEAENSNTEQNDNNNNDNNNNI NNNNNNNDNKEEDMNENNNNSKVTGDSVENINEQTNNNQYP	290
PS183\	EDVLLIETITINNGTTSNTIENNKDSNKEAENSNTEQNDNNNNDNNNNI NNNNNNNDNKEEDMNENNNNSKVTGDSVENINEQTNNNQYP	290
PS186\	EDVLLIETITINNGTTSNTIENNKDSNKEAENSNTEQNDNNNNDNNNNI NNNNNNNDNKEEDMNENNNNSKVTGDSVENINEQTNNNQYP	290
PS189\	EDVLLIETITINNGTTSNTIENNKDSNKEAENSNTEQNDNNNNDNNNNI NNNNNNNDNKEEDMNENNNNSKVTGDSVENINEQTNNNQYP	290
PS206_E11\	EDVLLIETITINNGTTSNTIENNKDSNKEAENSNTEQNDNNNNDNNNNI NNNNNNNDNKEEDMNENNNNSKVTGDSVENINEQTNNNQYP	290
PS250\	EDVLLIETITINNGTTSNTIENNKDSNKEAENSNTEQNDNNNNDNNNNI NNNNNNNDNKEEDMNENNNNSKVTGDSVENINEQTNNNQYP	290
RV_3600\	EDVLLIETITINNGTTSNTIENNKDSNKEAENSNTEQNDNNNNDNNNNI NNNNNNNDNKEEDMNENNNNSKVTGDSVENINEQTNNNQYP	290
RV_3606\	EDVLLIETITINNGTTSNTIENNKDSNKEAENSNTEQNDNNNNDNNNNI NNNNNNNDNKEEDMNENNNNSKVTGDSVENINEQTNNNQYP	290
RV_3610\	EDVLLIETITINNGTTSNTIENNKDSNKEAENSNTEQNDNNNNDNNNNI NNNNNNNDNKEEDMNENNNNSKVTGDSVENINEQTNNNQYP	290
RV_3611\	EDVLLIETITINNGTTSNTIENNKDSNKEAENSNTEQNDNNNNDNNNNI NNNNNNNDNKEEDMNENNNNSKVTGDSVENINEQTNNNQYP	290
RV_3614\	EDVLLIETITINNGTTSNTIENNKDSNKEAENSNTEQNDNNNNDNNNNI NNNNNNNDNKEEDMNENNNNSKVTGDSVENINEQTNNNQYP	290
RV_3630\	EDVLLIETITINNGTTSNTIENNKDSNKEAENSNTEQNDNNNNDNNNNI NNNNNNNDNKEEDMNENNNNSKVTGDSVENINEQTNNNQYP	290
RV_3635\	EDVLLIETITINNGTTSNTIENNKDSNKEAENSNTEQNDNNNNDNNNNI NNNNNNNDNKEEDMNENNNNSKVTGDSVENINEQTNNNQYP	290
RV_3637\	EDVLLIETITINNGTTSNTIENNKDSNKEAENSNTEQNDNNNNDNNNNI NNNNNNNDNKEEDMNENNNNSKVTGDSVENINEQTNNNQYP	290
RV_3642\	EDVLLIETITINNGTTSNTIENNKDSNKEAENSNTEQNDNNNNDNNNNI NNNNNNNDNKEEDMNENNNNSKVTGDSVENINEQTNNNQYP	290
RV_3650\	EDVLLIETITINNGTTSNTIENNKDSNKEAENSNTEQNDNNNNDNNNNI NNNNNNNDNKEEDMNENNNNSKVTGDSVENINEQTNNNQYP	290
RV_3655\	EDVLLIETITINNGTTSNTIENNKDSNKEAENSNTEQNDNNNNDNNNNI NNNNNNNDNKEEDMNENNNNSKVTGDSVENINEQTNNNQYP	290
RV_3671\	EDVLLIETITINNGTTSNTIENNKDSNKEAENSNTEQNDNNNNDNNNNI NNNNNNNDNKEEDMNENNNNSKVTGDSVENINEQTNNNQYP	290
RV_3672\	EDVLLIETITINNGTTSNTIENNKDSNKEAENSNTEQNDNNNNDNNNNI NNNNNNNDNKEEDMNENNNNSKVTGDSVENINEQTNNNQYP	290
RV_3673\	EDVLLIETITINNGTTSNTIENNKDSNKEAENSNTEQNDNNNNDNNNNI NNNNNNNDNKEEDMNENNNNSKVTGDSVENINEQTNNNQYP	290
RV_3675\	EDVLLIETITINNGTTSNTIENNKDSNKEAENSNTEQNDNNNNDNNNNI NNNNNNNDNKEEDMNENNNNSKVTGDSVENINEQTNNNQYP	290
RV_3687\	EDVLLIETITINNGTTSNTIENNKDSNKEAENSNTEQNDNNNNDNNNNI NNNNNNNDNKEEDMNENNNNSKVTGDSVENINEQTNNNQYP	290
RV_3695\	EDVLLIETITINNGTTSNTIENNKDSNKEAENSNTEQNDNNNNDNNNNI NNNNNNNDNKEEDMNENNNNSKVTGDSVENINEQTNNNQYP	290
RV_3696\	EDVLLIETITINNGTTSNTIENNKDSNKEAENSNTEQNDNNNNDNNNNI NNNNNNNDNKEEDMNENNNNSKVTGDSVENINEQTNNNQYP	290
RV_3701\	EDVLLIETITINNGTTSNTIENNKDSNKEAENSNTEQNDNNNNDNNNNI NNNNNNNDNKEEDMNENNNNSKVTGDSVENINEQTNNNQYP	290
RV_3702\	EDVLLIETITINNGTTSNTIENNKDSNKEAENSNTEQNDNNNNDNNNNI NNNNNNNDNKEEDMNENNNNSKVTGDSVENINEQTNNNQYP	290
RV_3703\	EDVLLIETITINNGTTSNTIENNKDSNKEAENSNTEQNDNNNNDNNNNI NNNNNNNDNKEEDMNENNNNSKVTGDSVENINEQTNNNQYP	290
RV_3708\	EDVLLIETITINNGTTSNTIENNKDSNKEAENSNTEQNDNNNNDNNNNI NNNNNNNDNKEEDMNENNNNSKVTGDSVENINEQTNNNQYP	290
RV_3714\	EDVLLIETITINNGTTSNTIENNKDSNKEAENSNTEQNDNNNNDNNNNI NNNNNNNDNKEEDMNENNNNSKVTGDSVENINEQTNNNQYP	290
RV_3717\	EDVLLIETITINNGTTSNTIENNKDSNKEAENSNTEQNDNNNNDNNNNI NNNNNNNDNKEEDMNENNNNSKVTGDSVENINEQTNNNQYP	290
RV_3729\	EDVLLIETITINNGTTSNTIENNKDSNKEAENSNTEQNDNNNNDNNNNI NNNNNNNDNKEEDMNENNNNSKVTGDSVENINEQTNNNQYP	290
RV_3730\	EDVLLIETITINNGTTSNTIENNKDSNKEAENSNTEQNDNNNNDNNNNI NNNNNNNDNKEEDMNENNNNSKVTGDSVENINEQTNNNQYP	290
RV_3731\	EDVLLIETITINNGTTSNTIENNKDSNKEAENSNTEQNDNNNNDNNNNI NNNNNNNDNKEEDMNENNNNSKVTGDSVENINEQTNNNQYP	290
RV_3735\	EDVLLIETITINNGTTSNTIENNKDSNKEAENSNTEQNDNNNNDNNNNI NNNNNNNDNKEEDMNENNNNSKVTGDSVENINEQTNNNQYP	290
RV_3736\	EDVLLIETITINNGTTSNTIENNKDSNKEAENSNTEQNDNNNNDNNNNI NNNNNNNDNKEEDMNENNNNSKVTGDSVENINEQTNNNQYP	290
RV_3737\	EDVLLIETITINNGTTSNTIENNKDSNKEAENSNTEQNDNNNNDNNNNI NNNNNNNDNKEEDMNENNNNSKVTGDSVENINEQTNNNQYP	290
RV_3739\	EDVLLIETITINNGTTSNTIENNKDSNKEAENSNTEQNDNNNNDNNNNI NNNNNNNDNKEEDMNENNNNSKVTGDSVENINEQTNNNQYP	290
RV_3740\	EDVLLIETITINNGTTSNTIENNKDSNKEAENSNTEQNDNNNNDNNNNI NNNNNNNDNKEEDMNENNNNSKVTGDSVENINEQTNNNQYP	290
RV_3741\	EDVLLIETITINNGTTSNTIENNKDSNKEAENSNTEQNDNNNNDNNNNI NNNNNNNDNKEEDMNENNNNSKVTGDSVENINEQTNNNQYP	290
RV_3764\	EDVLLIETITINNGTTSNTIENNKDSNKEAENSNTEQNDNNNNDNNNNI NNNNNNNDNKEEDMNENNNNSKVTGDSVENINEQTNNNQYP	290
RV_3766\	EDVLLIETITINNGTTSNTIENNKDSNKEAENSNTEQNDNNNNDNNNNI NNNNNNNDNKEEDMNENNNNSKVTGDSVENINEQTNNNQYP	290
RV_3769\	EDVLLIETITINNGTTSNTIENNKDSNKEAENSNTEQNDNNNNDNNNNI NNNNNNNDNKEEDMNENNNNSKVTGDSVENINEQTNNNQYP	290
SantaLucia_SalvadorI\	EDVLLIETITINNGTTSNTIENNKDSNKEAENSNTEQNDNNNNDNNNNI NNNNNNNDNKEEDMNENNNNSKVTGDSVENINEQTNNNQYP	290
SD01\	EDVLLIETITINNGTTSNTIENNKDSNKEAENSNTEQNDNNNNDNNNNI NNNNNNNDNKEEDMNENNNNSKVTGDSVENINEQTNNNQYP	290
SenT001.08\	EDVLLIETITINNGTTSNTIENNKDSNKEAENSNTEQNDNNNNDNNNNI NNNNNNNDNKEEDMNENNNNSKVTGDSVENINEQTNNNQYP	290
SenT002.07\	EDVLLIETITINNGTTSNTIENNKDSNKEAENSNTEQNDNNNNDNNNNI NNNNNNNDNKEEDMNENNNNSKVTGDSVENINEQTNNNQYP	290
SenT002.09\	EDVLLIETITINNGTTSNTIENNKDSNKEAENSNTEQNDNNNNDNNNNI NNNNNNNDNKEEDMNENNNNSKVTGDSVENINEQTNNNQYP	290
SenT015.09\	EDVLLIETITINNGTTSNTIENNKDSNKEAENSNTEQNDNNNNDNNNNI NNNNNNNDNKEEDMNENNNNSKVTGDSVENINEQTNNNQYP	290
SenT018.09\	EDVLLIETITINNGTTSNTIENNKDSNKEAENSNTEQNDNNNNDNNNNI NNNNNNNDNKEEDMNENNNNSKVTGDSVENINEQTNNNQYP	290
SenT021.09\	EDVLLIETITINNGTTSNTIENNKDSNKEAENSNTEQNDNNNNDNNNNI NNNNNNNDNKEEDMNENNNNSKVTGDSVENINEQTNNNQYP	290
SenT022.09\	EDVLLIETITINNGTTSNTIENNKDSNKEAENSNTEQNDNNNNDNNNNI NNNNNNNDNKEEDMNENNNNSKVTGDSVENINEQTNNNQYP	290
SenT024.08\	EDVLLIETITINNGTTSNTIENNKDSNKEAENSNTEQNDNNNNDNNNNI NNNNNNNDNKEEDMNENNNNSKVTGDSVENINEQTNNNQYP	290
SenT029.09\	EDVLLIETITINNGTTSNTIENNKDSNKEAENSNTEQNDNNNNDNNNNI NNNNNNNDNKEEDMNENNNNSKVTGDSVENINEQTNNNQYP	290
SenT032.09\	EDVLLIETITINNGTTSNTIENNKDSNKEAENSNTEQNDNNNNDNNNNI NNNNNNNDNKEEDMNENNNNSKVTGDSVENINEQTNNNQYP	290
SenT033.08\	EDVLLIETITINNGTTSNTIENNKDSNKEAENSNTEQNDNNNNDNNNNI NNNNNNNDNKEEDMNENNNNSKVTGDSVENINEQTNNNQYP	290
SenT033.09\	EDVLLIETITINNGTTSNTIENNKDSNKEAENSNTEQNDNNNNDNNNNI NNNNNNNDNKEEDMNENNNNSKVTGDSVENINEQTNNNQYP	290
SenT042.09\	EDVLLIETITINNGTTSNTIENNKDSNKEAENSNTEQNDNNNNDNNNNI NNNNNNNDNKEEDMNENNNNSKVTGDSVENINEQTNNNQYP	290
SenT044.10\	EDVLLIETITINNGTTSNTIENNKDSNKEAENSNTEQNDNNNNDNNNNI NNNNNNNDNKEEDMNENNNNSKVTGDSVENINEQTNNNQYP	290
SenT046.10\	EDVLLIETITINNGTTSNTIENNKDSNKEAENSNTEQNDNNNNDNNNNI NNNNNNNDNKEEDMNENNNNSKVTGDSVENINEQTNNNQYP	290
SenT047.09\	EDVLLIETITINNGTTSNTIENNKDSNKEAENSNTEQNDNNNNDNNNNI NNNNNNNDNKEEDMNENNNNSKVTGDSVENINEQTNNNQYP	290
SenT061.09\	EDVLLIETITINNGTTSNTIENNKDSNKEAENSNTEQNDNNNNDNNNNI NNNNNNNDNKEEDMNENNNNSKVTGDSVENINEQTNNNQYP	290
SenT063.07\	EDVLLIETITINNGTTSNTIENNKDSNKEAENSNTEQNDNNNNDNNNNI NNNNNNNDNKEEDMNENNNNSKVTGDSVENINEQTNNNQYP	290
SenT066.08\	EDVLLIETITINNGTTSNTIENNKDSNKEAENSNTEQNDNNNNDNNNNI NNNNNNNDNKEEDMNENNNNSKVTGDSVENINEQTNNNQYP	290
SenT067.09\	EDVLLIETITINNGTTSNTIENNKDSNKEAENSNTEQNDNNNNDNNNNI NNNNNNNDNKEEDMNENNNNSKVTGDSVENINEQTNNNQYP	290
SenT075.10\	EDVLLIETITINNGTTSNTIENNKDSNKEAENSNTEQNDNNNNDNNNNI NNNNNNNDNKEEDMNENNNNSKVTGDSVENINEQTNNNQYP	290
SenT077.08\	EDVLLIETITINNGTTSNTIENNKDSNKEAENSNTEQNDNNNNDNNNNI NNNNNNNDNKEEDMNENNNNSKVTGDSVENINEQTNNNQYP	290
SenT087.08\	EDVLLIETITINNGTTSNTIENNKDSNKEAENSNTEQNDNNNNDNNNNI NNNNNNNDNKEEDMNENNNNSKVTGDSVENINEQTNNNQYP	290
SenT090.08\	EDVLLIETITINNGTTSNTIENNKDSNKEAENSNTEQNDNNNNDNNNNI NNNNNNNDNKEEDMNENNNNSKVTGDSVENINEQTNNNQYP	290
SenT090.09\	EDVLLIETITINNGTTSNTIENNKDSNKEAENSNTEQNDNNNNDNNNNI NNNNNNNDNKEEDMNENNNNSKVTGDSVENINEQTNNNQYP	290
SenT093.09\	EDVLLIETITINNGTTSNTIENNKDSNKEAENSNTEQNDNNNNDNNNNI NNNNNNNDNKEEDMNENNNNSKVTGDSVENINEQTNNNQYP	290
SenT094.09\	EDVLLIETITINNGTTSNTIENNKDSNKEAENSNTEQNDNNNNDNNNNI NNNNNNNDNKEEDMNENNNNSKVTGDSVENINEQTNNNQYP	290
SenT097.09\	EDVLLIETITINNGTTSNTIENNKDSNKEAENSNTEQNDNNNNDNNNNI NNNNNNNDNKEEDMNENNNNSKVTGDSVENINEQTNNNQYP	290
SenT101.09\	EDVLLIETITINNGTTSNTIENNKDSNKEAENSNTEQNDNNNNDNNNNI NNNNNNNDNKEEDMNENNNNSKVTGDSVENINEQTNNNQYP	290
SenT102.08\	EDVLLIETITINNGTTSNTIENNKDSNKEAENSNTEQNDNNNNDNNNNI NNNNNNNDNKEEDMNENNNNSKVTGDSVENINEQTNNNQYP	290
SenT106.08\	EDVLLIETITINNGTTSNTIENNKDSNKEAENSNTEQNDNNNNDNNNNI NNNNNNNDNKEEDMNENNNNSKVTGDSVENINEQTNNNQYP	290
SenT113.09\	EDVLLIETITINNGTTSNTIENNKDSNKEAENSNTEQNDNNNNDNNNNI NNNNNNNDNKEEDMNENNNNSKVTGDSVENINEQTNNNQYP	290

SenT119.09\	EDVLLIETITINNGTTSNTIENNKDSNKEAENSNTEQNDNNNNDNNNNINNNNNNNDNKEEDMNENNNNSKVTGDSVENINEQTNNNQYP	290
SenT123.09\	EDVLLIETITINNGTTSNTIENNKDSNKEAENSNTEQNDNNNNDNNNNINNNNNNNDNKEEDMNENNNNSKVTGDSVENINEQTNNNQYP	290
SenT127.09\	EDVLLIETITINNGTTSNTIENNKDSNKEAENSNTEQNDNNNNDNNNNINNNNNNNDNKEEDMNENNNNSKVTGDSVENINEQTNNNQYP	290
SenT128.08\	EDVLLIETITINNGTTSNTIENNKDSNKEAENSNTEQNDNNNNDNNNNINNNNNNNDNKEEDMNENNNNSKVTGDSVENINEQTNNNQYP	290
SenT135.09\	EDVLLIETITINNGTTSNTIENNKDSNKEAENSNTEQNDNNNNDNNNNINNNNNNNDNKEEDMNENNNNSKVTGDSVENINEQTNNNQYP	290
SenT137.08\	EDVLLIETITINNGTTSNTIENNKDSNKEAENSNTEQNDNNNNDNNNNINNNNNNNDNKEEDMNENNNNSKVTGDSVENINEQTNNNQYP	290
SenT137.09\	EDVLLIETITINNGTTSNTIENNKDSNKEAENSNTEQNDNNNNDNNNNINNNNNNNDNKEEDMNENNNNSKVTGDSVENINEQTNNNQYP	290
SenT139.08\	EDVLLIETITINNGTTSNTIENNKDSNKEAENSNTEQNDNNNNDNNNNINNNNNNNDNKEEDMNENNNNSKVTGDSVENINEQTNNNQYP	290
SenT140.08\	EDVLLIETITINNGTTSNTIENNKDSNKEAENSNTEQNDNNNNDNNNNINNNNNNNDNKEEDMNENNNNSKVTGDSVENINEQTNNNQYP	290
SenT142.09\	EDVLLIETITINNGTTSNTIENNKDSNKEAENSNTEQNDNNNNDNNNNINNNNNNNDNKEEDMNENNNNSKVTGDSVENINEQTNNNQYP	290
SenT144.08\	EDVLLIETITINNGTTSNTIENNKDSNKEAENSNTEQNDNNNNDNNNNINNNNNNNDNKEEDMNENNNNSKVTGDSVENINEQTNNNQYP	290
SenT145.08\	EDVLLIETITINNGTTSNTIENNKDSNKEAENSNTEQNDNNNNDNNNNINNNNNNNDNKEEDMNENNNNSKVTGDSVENINEQTNNNQYP	290
SenT149.09\	EDVLLIETITINNGTTSNTIENNKDSNKEAENSNTEQNDNNNNDNNNNINNNNNNNDNKEEDMNENNNNSKVTGDSVENINEQTNNNQYP	290
SenT150.09\	EDVLLIETITINNGTTSNTIENNKDSNKEAENSNTEQNDNNNNDNNNNINNNNNNNDNKEEDMNENNNNSKVTGDSVENINEQTNNNQYP	290
SenT151.09\	EDVLLIETITINNGTTSNTIENNKDSNKEAENSNTEQNDNNNNDNNNNINNNNNNNDNKEEDMNENNNNSKVTGDSVENINEQTNNNQYP	290
SenT170.08\	EDVLLIETITINNGTTSNTIENNKDSNKEAENSNTEQNDNNNNDNNNNINNNNNNNDNKEEDMNENNNNSKVTGDSVENINEQTNNNQYP	290
SenT175.08\	EDVLLIETITINNGTTSNTIENNKDSNKEAENSNTEQNDNNNNDNNNNINNNNNNNDNKEEDMNENNNNSKVTGDSVENINEQTNNNQYP	290
SenT179.08\	EDVLLIETITINNGTTSNTIENNKDSNKEAENSNTEQNDNNNNDNNNNINNNNNNNDNKEEDMNENNNNSKVTGDSVENINEQTNNNQYP	290
SenT180.08\	EDVLLIETITINNGTTSNTIENNKDSNKEAENSNTEQNDNNNNDNNNNINNNNNNNDNKEEDMNENNNNSKVTGDSVENINEQTNNNQYP	290
SenT190.08\	EDVLLIETITINNGTTSNTIENNKDSNKEAENSNTEQNDNNNNDNNNNINNNNNNNDNKEEDMNENNNNSKVTGDSVENINEQTNNNQYP	290
SenT197.08\	EDVLLIETITINNGTTSNTIENNKDSNKEAENSNTEQNDNNNNDNNNNINNNNNNNDNKEEDMNENNNNSKVTGDSVENINEQTNNNQYP	290
SenT224.08\	EDVLLIETITINNGTTSNTIENNKDSNKEAENSNTEQNDNNNNDNNNNINNNNNNNDNKEEDMNENNNNSKVTGDSVENINEQTNNNQYP	290
SenT227.08\	EDVLLIETITINNGTTSNTIENNKDSNKEAENSNTEQNDNNNNDNNNNINNNNNNNDNKEEDMNENNNNSKVTGDSVENINEQTNNNQYP	290
SenT230.08\	EDVLLIETITINNGTTSNTIENNKDSNKEAENSNTEQNDNNNNDNNNNINNNNNNNDNKEEDMNENNNNSKVTGDSVENINEQTNNNQYP	290
SenT235.08\	EDVLLIETITINNGTTSNTIENNKDSNKEAENSNTEQNDNNNNDNNNNINNNNNNNDNKEEDMNENNNNSKVTGDSVENINEQTNNNQYP	290
SenT238.08\	EDVLLIETITINNGTTSNTIENNKDSNKEAENSNTEQNDNNNNDNNNNINNNNNNNDNKEEDMNENNNNSKVTGDSVENINEQTNNNQYP	290
SEV2_H5\	EDVLLIETITINNGTTSNTIENNKDSNKEAENSNTEQNDNNNNDNNNNINNNNNNNDNKEEDMNENNNNSKVTGDSVENINEQTNNNQYP	290
SN01\	EDVLLIETITINNGTTSNTIENNKDSNKEAENSNTEQNDNNNNDNNNNINNNNNNNDNKEEDMNENNNNSKVTGDSVENINEQTNNNQYP	290
TG01\	EDVLLIETITINNGTTSNTIENNKDSNKEAENSNTEQNDNNNNDNNNNINNNNNNNDNKEEDMNENNNNSKVTGDSVENINEQTNNNQYP	290
TRIPS_301\	EDVLLIETITINNGTTSNTIENNKDSNKEAENSNTEQNDNNNNDNNNNINNNNNNNDNKEEDMNENNNNSKVTGDSVENINEQTNNNQYP	290
TRIPS_303\	EDVLLIETITINNGTTSNTIENNKDSNKEAENSNTEQNDNNNNDNNNNINNNNNNNDNKEEDMNENNNNSKVTGDSVENINEQTNNNQYP	290
TRIPS_331\	EDVLLIETITINNGTTSNTIENNKDSNKEAENSNTEQNDNNNNDNNNNINNNNNNNDNKEEDMNENNNNSKVTGDSVENINEQTNNNQYP	290
TRIPS_355\	EDVLLIETITINNGTTSNTIENNKDSNKEAENSNTEQNDNNNNDNNNNINNNNNNNDNKEEDMNENNNNSKVTGDSVENINEQTNNNQYP	290
TRIPS_364\	EDVLLIETITINNGTTSNTIENNKDSNKEAENSNTEQNDNNNNDNNNNINNNNNNNDNKEEDMNENNNNSKVTGDSVENINEQTNNNQYP	290
TRIPS_373\	EDVLLIETITINNGTTSNTIENNKDSNKEAENSNTEQNDNNNNDNNNNINNNNNNNDNKEEDMNENNNNSKVTGDSVENINEQTNNNQYP	290
TRIPS_410\	EDVLLIETITINNGTTSNTIENNKDSNKEAENSNTEQNDNNNNDNNNNINNNNNNNDNKEEDMNENNNNSKVTGDSVENINEQTNNNQYP	290
TRIPS_433\	EDVLLIETITINNGTTSNTIENNKDSNKEAENSNTEQNDNNNNDNNNNINNNNNNNDNKEEDMNENNNNSKVTGDSVENINEQTNNNQYP	290
TRIPS_437\	EDVLLIETITINNGTTSNTIENNKDSNKEAENSNTEQNDNNNNDNNNNINNNNNNNDNKEEDMNENNNNSKVTGDSVENINEQTNNNQYP	290
TRIPS_440\	EDVLLIETITINNGTTSNTIENNKDSNKEAENSNTEQNDNNNNDNNNNINNNNNNNDNKEEDMNENNNNSKVTGDSVENINEQTNNNQYP	290
TRIPS_456\	EDVLLIETITINNGTTSNTIENNKDSNKEAENSNTEQNDNNNNDNNNNINNNNNNNDNKEEDMNENNNNSKVTGDSVENINEQTNNNQYP	290
TRIPS_461\	EDVLLIETITINNGTTSNTIENNKDSNKEAENSNTEQNDNNNNDNNNNINNNNNNNDNKEEDMNENNNNSKVTGDSVENINEQTNNNQYP	290
TRIPS_467\	EDVLLIETITINNGTTSNTIENNKDSNKEAENSNTEQNDNNNNDNNNNINNNNNNNDNKEEDMNENNNNSKVTGDSVENINEQTNNNQYP	290
TRIPS_470\	EDVLLIETITINNGTTSNTIENNKDSNKEAENSNTEQNDNNNNDNNNNINNNNNNNDNKEEDMNENNNNSKVTGDSVENINEQTNNNQYP	290
TRIPS_474\	EDVLLIETITINNGTTSNTIENNKDSNKEAENSNTEQNDNNNNDNNNNINNNNNNNDNKEEDMNENNNNSKVTGDSVENINEQTNNNQYP	290
TRIPS_480\	EDVLLIETITINNGTTSNTIENNKDSNKEAENSNTEQNDNNNNDNNNNINNNNNNNDNKEEDMNENNNNSKVTGDSVENINEQTNNNQYP	290
TRIPS_482\	EDVLLIETITINNGTTSNTIENNKDSNKEAENSNTEQNDNNNNDNNNNINNNNNNNDNKEEDMNENNNNSKVTGDSVENINEQTNNNQYP	290
TRIPS_487\	EDVLLIETITINNGTTSNTIENNKDSNKEAENSNTEQNDNNNNDNNNNINNNNNNNDNKEEDMNENNNNSKVTGDSVENINEQTNNNQYP	290
TRIPS_490\	EDVLLIETITINNGTTSNTIENNKDSNKEAENSNTEQNDNNNNDNNNNINNNNNNNDNKEEDMNENNNNSKVTGDSVENINEQTNNNQYP	290
TRIPS_499\	EDVLLIETITINNGTTSNTIENNKDSNKEAENSNTEQNDNNNNDNNNNINNNNNNNDNKEEDMNENNNNSKVTGDSVENINEQTNNNQYP	290
TRIPS_501\	EDVLLIETITINNGTTSNTIENNKDSNKEAENSNTEQNDNNNNDNNNNINNNNNNNDNKEEDMNENNNNSKVTGDSVENINEQTNNNQYP	290
TRIPS_504\	EDVLLIETITINNGTTSNTIENNKDSNKEAENSNTEQNDNNNNDNNNNINNNNNNNDNKEEDMNENNNNSKVTGDSVENINEQTNNNQYP	290
TRIPS_700\	EDVLLIETITINNGTTSNTIENNKDSNKEAENSNTEQNDNNNNDNNNNINNNNNNNDNKEEDMNENNNNSKVTGDSVENINEQTNNNQYP	290
TRIPS_704\	EDVLLIETITINNGTTSNTIENNKDSNKEAENSNTEQNDNNNNDNNNNINNNNNNNDNKEEDMNENNNNSKVTGDSVENINEQTNNNQYP	290
TRIPS_708\	EDVLLIETITINNGTTSNTIENNKDSNKEAENSNTEQNDNNNNDNNNNINNNNNNNDNKEEDMNENNNNSKVTGDSVENINEQTNNNQYP	290
TRIPS_759\	EDVLLIETITINNGTTSNTIENNKDSNKEAENSNTEQNDNNNNDNNNNINNNNNNNDNKEEDMNENNNNSKVTGDSVENINEQTNNNQYP	290

Decoration 'Decoration #1': Shade (with bright yellow at 10% fill) residues that match the Consensus exactly.

In box are Asian isolates

incomplete sequences were removed from the alignment
